# Compression therapies for venous leg ulcers: The VENous Ulcer Study 6 (VenUS 6), an open, multicentre, randomised clinical trial

**DOI:** 10.1371/journal.pmed.1005154

**Published:** 2026-07-10

**Authors:** Catherine Arundel, Charlie Welch, Luke Strachan, Charlie Peck, Ross Atkinson, Una Adderley, Ian Chetter, Nicky Cullum, Tom Davill, Jane Griffiths, Catherine Hewitt, Charlotte Hirst, Katherine Jones, Maartje Kletter, Julie Mullings, Han Phung, Gareth Roberts, Pedro Saramago, Brigid Smart, Marta Soares, Philip Stather, Nikki Stubbs, Jude Watson, Sabeen Zahra, Jo Dumville

**Affiliations:** 1 York Trials Unit, Department of Health Sciences, Faculty of Science, University of York, York, United Kingdom; 2 Division of Nursing, Midwifery and Social Work, School of Health Sciences, University of Manchester, Manchester, United Kingdom; 3 Community Services, Manchester University NHS Foundation Trust, Manchester, United Kingdom; 4 Faculty of Health Sciences, University of Hull, Hull, United Kingdom; 5 Hull York Medical School, University of Hull, Hull, United Kingdom; 6 Vascular Service, Hull University Teaching Hospitals NHS Trust, Hull, United Kingdom; 7 Warwick Clinical Trials Unit, Warwick Medical School, University of Warwick, Coventry, United Kingdom; 8 Centre for Health Economics, University of York, York, United Kingdom; 9 Department of Vascular Surgery, Norfolk and Norwich University Hospital NHS Foundation Trust, Norwich, United Kingdom; 10 Vascular Surgery, Addenbrookes Hospital NHS Foundation Trust, Cambridge, United Kingdom; 11 Norwich Medical School, University of East Anglia, Norwich, United Kingdom; 12 NCS Woundcare Consulting Limited, Leeds, United Kingdom; Barts and the London School of Medicine & Dentistry Queen Mary University of London, UNITED KINGDOM OF GREAT BRITAIN AND NORTHERN IRELAND

## Abstract

**Background:**

Strong compression is a recommended first line venous leg ulcer treatment. With limited research comparing the clinical effectiveness of compression wraps (CW) and two-layer compression bandage treatments with evidence-based compression (EBC) (four-layer compression bandages and two-layer compression hosiery), this study aimed to evaluate their clinical effectiveness on time to venous leg ulcer healing.

**Methods and findings:**

A pragmatic, three-arm, randomised controlled trial in 33 United Kingdom primary, community and hospital sites between 03.02.2021 and 31.08.2024. Adults with a venous leg ulcer appropriate for compression therapy were randomised 1:1:1 to be offered CW, two-layer bandage, or EBC (two-layer hosiery or four-layer bandage). Participants and clinical staff were not blinded. The primary outcome was time to blind assessed ulcer healing (date of ulcer healing: date of earliest photograph showing healing). Analyses included a noninferiority comparison of two-layer bandage and EBC (handling key intercurrent events under hypothetical and treatment policy strategies), and superiority comparisons of CW with both EBC and two-layer bandage (handling key intercurrent events under a treatment policy strategy). Healing times were analysed using Cox proportional hazards regression adjusted for fixed effects (treatment allocation, baseline ulcer area and duration, participant age, and mobility status), and shared frailties (recruitment site). The trial was pre-registered: ISRCTN67321719.

637 participants were randomised to be offered CW (*n* = 213), two-layer bandage (*n* = 211) or EBC (*n* = 213). Mean age was 70.3 (range 24.6 to 97.0) years, 55% (*n* = 351) were male, and the majority (*n* = 606, 95%) were white. 633 participants contributed time at risk of healing and were included in the analysis.

Using a treatment policy strategy to handle key intercurrent events (modified intention-to-treat analysis), the estimated hazard ratio (HR) for the noninferiority comparison (EBC and two-layer bandage) was 1.01 (95% CI [0.79, 1.28]), meeting the pre-specified noninferiority margin of 1.33. The corresponding hypothetical strategy analysis gave a HR of 1.16 (95% CI [0.86, 1.58]), which did not demonstrate noninferiority.

For the superiority comparisons, healing was slower in the CW group than in the EBC group (HR 0.78, 95% CI [0.61, 1.00]; *p* = 0.046). Results were similar for the two-layer bandage group (HR 0.79, 95% CI [0.61, 1.01]; *p* = 0.056), although this did not reach statistical significance. Both comparisons showed considerable statistical uncertainty, with confidence intervals ranging from a 39% reduction in the hazard of healing to little or no difference between groups.

Nine serious adverse events occurred; one potentially related to treatment (cause of death could not be ascertained). Departures from allocated compression treatment were common, which limits generalisability to settings with different adherence patterns. These departures, lower than expected ulcer healing incidence rates and slight under-recruitment, resulted in the number of healing events being smaller than the number required for 80% power.

**Conclusion:**

CW is unlikely to reduce the time to venous leg ulcer healing compared to two-layer bandage or EBC, although confidence intervals included treatment effects indicating little or no difference between groups. Despite remaining uncertainty, these findings may not support CW as a first line strong compression treatment for venous leg ulcers.

**Trial registration:**

ISRCTN – reference 67321719.

## Author summary

**Why was this study done?:**

- Different types of compression are used to treat venous leg ulcers.
- Some treatments are known to be good at helping these ulcers to heal (four-layer compression bandages and two-layer compression hosiery), however this was not currently known for some treatments (compression wraps and two-layer compression bandage treatments).

**What did the researchers do and find?:**

- VenUS 6 recruited 637 participants, who were equally and randomly assigned to be offered compression wraps, two-layer bandage, or evidence-based compression (four-layer bandage or two-layer hosiery).
- Compared with evidenced based compression (four-layer bandage or two-layer hosiery) and the two-layer bandage, compression wraps increase the time it takes for venous leg ulcers to heal.

**What do these findings mean?:**

- Compression remains an important treatment for people with venous leg ulcers.
- Compression wraps are unlikely to reduce the time to healing when compared to the alternate compression therapies.
- They are therefore unlikely to be an optimal first line treatment to promote venous leg ulcer healing.
- The number of participants who experienced ulcer healing was lower than those anticipated when the study started. This means the study had a lower chance of finding a real difference between the treatments if one did exist.

## Abstract 3

In this open, multicentre, randomised clinical trial, Catherine Arundel and colleagues evaluate the clinical effectiveness of three venous leg ulcer treatments: compression wraps, two-layer compression bandages, and evidence-based compression (four-layer compression bandages and two-layer compression hosiery).

## Introduction

Venous leg ulcers are open wounds which lie wholly or partially within the gaiter region of the lower leg, and which are primarily caused by impaired venous blood flow in the legs, leading to venous hypertension and associated complications that include these complex wounds [1,2]. The prevalence of these common, chronic, wounds increases with age [3], and can seriously impact health-related quality of life [4–8].

Strong compression is defined as an elastic compression system that is intended to apply ≥40 mmHg at the ankle or a nonelastic (e.g., short stretch) system applied in accordance with manufacturers’ recommendations. Compression provides graduated pressure from the ankle (highest pressure) to the knee to reduce venous hypertension. Strong compression is an evidence-based first line treatment for venous leg ulcers that reduces time to healing [9,10].

Commonly used types of strong compression therapy are two- or four-layer bandage systems that contain an elastic layer; two-layer hosiery (stockings); compression wraps (CW) (removable wraps held in place with hook and loop fastenings); and inelastic short stretch bandages (one–three layers). Whilst all types apply graduated pressure, they have different modes of application. Bandage application usually requires skill and training, whilst two-layer hosiery (2LH) and CW allow application by the patient or an untrained carer. Once applied, compression therapies can feel tight, uncomfortable, and bulky and these factors may impact adherence and thus relative effectiveness [11,12].

Previous randomised controlled trials (RCTs) have identified that four-layer bandage (4LB) and 2LH confer similar ulcer healing times [13]. 4LB has also been found to be superior to short stretch bandage [14]. Whilst a comprehensive systematic review and individual patient data meta-analyses (*n* = 797) for all strong compression therapies found two-layer bandage (2LB) to have the highest probability of being clinically effective, the underpinning RCT evidence was of low or very low quality and findings were uncertain meaning that the need for more robust research was noted [13,15]. Whilst the newer 2LB is widely used due to quicker and easier application than 4LB, only three trials (*n* = 299 participants) and limited follow-up have been published [16–18]. CW may be appealing because of their purported ease of self-application but also have a limited evidence base. There are only two small RCTs of CW (*n* = 78 participants) and neither reported time to ulcer healing [19,20].

Given the limited available evidence for 2LB and CW, VenUS 6 sought to assess the clinical effectiveness of these treatments for the healing of venous leg ulcers compared with the evidence-based compression (EBC) therapies (4LB or 2LH).

## Methods

### Study design and participants

VenUS 6 was a parallel group, multicentre, three arm randomised controlled trial, comparing the effects of an EBC treatment strategy (choice arm of the 4LB or 2LH), with 2LB and CW on venous leg ulcer healing. Both superiority comparisons (CW versus EBC and CW versus 2LB), and a noninferiority comparison (2LB versus EBC) were included. Separate publications report the cost-effectiveness and process evaluation analyses conducted in relation to this trial.

There were 33 UK recruiting sites covering primary, community, and hospital care settings.

UK ethical approval was obtained prior to study commencement from the West of Scotland Research Ethics Committee 4 (Reference 20-WS-0121) and the study was prospectively registered: ISRCTN67321719 (https://www.isrctn.com/ISRCTNISRCTN67321719; Date of registration 14.09.2020).

The protocol has previously been published and is included as a Supplementary File. Independent Trial Steering and Data Monitoring Committees were convened for oversight and review of study progress and conduct.

Eligible patients were aged ≥18 years with at least one venous leg ulcer lying wholly or partially within the gaiter region of the leg (i.e., between the mid-calf and the ankle). Patients were required to have had a negative assessment of peripheral arterial disease in the three months prior to eligibility assessment, be able to tolerate strong compression, and not be participating in another venous leg ulcer treatment study.

Patients were ineligible if they were allergic to any trial product, were deemed (at clinical discretion) to be clinically inappropriate to participate, or had treatment to close/remove incompetent superficial veins (e.g., via endovenous ablation, sclerotherapy) scheduled within 28 days of baseline.

Following screening, eligible patients were approached for written informed consent. Baseline questionnaires collecting demographic, ulcer history, ulcer pain, and quality of life information were completed prior to randomisation. The largest eligible ulcer at baseline was deemed the reference ulcer, and the leg on which it was located the reference leg.

### Randomisation and masking

Blocked randomisation (with randomly permuted blocks of varying sizes) was used to allocate participants 1:1:1 to the three treatment strategies. Randomisation was stratified by ulcer duration (≤6 months and >6 months) and ulcer area (≤5 cm^2^ and >5 cm^2^ measured as maximum ulcer length x maximum ulcer width), given these are recognised predictors of ulcer healing [21]. The allocation sequence was generated by the trial statistician prior to recruitment commencing and was implemented using a bespoke centralised secure online randomisation service (York Trials Unit, University of York), accessed via individual log on to ensure allocation concealment.

Participants and research teams could not be blinded to treatment allocation, however blinded assessment of wound photography was conducted to minimise potential bias for the primary outcome measurement [22].

### Procedures

All interventions were required to provide ≥40 mmHg compression at the ankle and were delivered in accordance with routine UK National Health Service (NHS) practice [10]. Concomitant medications or treatments were permitted as deemed necessary by the clinical care team and were recorded. The primary contact dressing was also selected by the clinical care team and was recorded.

Treatments were required to correspond to the following requirements, with further details for each treatment group provided in Table A in S1 File.

Where participants had bilateral ulceration, the treatment applied to the nonreference leg was at clinical discretion.

#### Compression wrap (CW) group.

An adjustable, European Conformity (CE) marked compression sleeve secured with hook and loop fastenings, marketed for venous leg ulceration treatment. Use of a compressive or noncompressive liner and/or foot compression elements was at the discretion of the treating health professional.

#### Evidence based compression (EBC) treatment strategy group.

4LB or 2LH with choice based on clinical judgement and/or patient preference. The decision to combine these two treatments was made based on previous RCT evidence which has identified that 4LB and 2LH confer similar ulcer healing times [13].

#### Two-layer bandage (2LB) group.

A recognised two-layer system consisting of an initial bandage layer covered with a top cohesive compression bandage.

Participants received their allocated treatment as soon as possible following randomisation, with the aim of continued receipt of this treatment until healing (or trial exit). However, participants could switch between compression treatments based on clinical need, or patient preference. These data were collected via treating health professionals as part of routine care.

### Outcomes

Participants were followed up via monthly telephone contact for primary and clinical secondary outcomes, and by postal questionnaires at 1-, 3-, 6-, and 12-months post-randomisation for patient reported secondary outcomes. Most participants were followed up for 12 months, however those recruited in the final 12 months of the study recruitment period were followed up for between 4 and 12 months; an approach used in other VenUS trials [13,23–25]. Various strategies were used to maximise questionnaire response rates including use of reminder letters and telephone calls, an unconditional incentive at the final follow-up in addition to two embedded studies within a trial evaluating effectiveness of a pen versus no pen, and newsletters and/or thank you cards versus no intervention [26].

Participants left the trial at the end of the follow-up period, if they withdrew consent or died.

#### Primary outcome.

Time to healing of the reference ulcer, defined as “complete epithelial cover in the absence of a scab with no dressing required” was measured in days from randomisation, assessed by blinded outcome assessors.

To reduce bias associated with subjective and unblinded outcome assessment such as ulcer healing [22], once healing was reported, digital photographs were taken for up to 4 weeks and were independently assessed, blinded to allocation, by experienced tissue viability nurses. A third reviewer (vascular surgeon) provided additional review if required. The date of the earliest photograph showing healing was taken to be the date of ulcer healing. The blinded assessment of the healing date was the primary healing endpoint with nonblinded assessment used as a secondary outcome.

Participants who were lost-to-follow-up or withdrew prior to reference ulcer healing had their healing times censored at the date of last study contact. Participants who remained unhealed by 12 months post-randomisation had their healing times censored at 12 months. Participants who were unhealed at the end of follow-up (31st August 2024) had their healing times censored at this date.

#### Secondary outcomes.

1) Time to: nurse reported reference ulcer healing, reference ulcer healing (either nurse reported or assessed by blinded outcome assessors), reference leg healing and ulcer recurrence.
2) Routinely assessed clinical events of interest (as secondary outcomes and as adverse events): incidence of ulcer/skin deterioration, ulcer infection, new ulcer occurrence, treatment of incompetent veins (e.g., ablation, sclerotherapy), hospital admissions, amputation, and death
3) Patient reported outcomes: health-related quality of life and ulcer symptom severity using the VEINES-QoL and VEINES-Sym [27], adherence to treatment and ease of use, and ulcer-related pain (0 no pain to 100 worst pain imaginable).

Outcomes relating to generic health-related quality of life and resource use were also collected, along with qualitative data pertaining to views and experiences of the treatments from participants and nurses. These outcomes will be reported in separate publications.

Study data were processed at the York Trials Unit (University of York, UK), using a licensed, automated, electronic system (Teleform), enabling data to be entered (scanned), checked, and validated.

### Statistical analysis

A hazard ratio (HR) of 1.33 was used as the noninferiority margin for the comparison of EBC and 2LB, based on the treatment effect observed in the VenUS I trial [13,23]. Assuming identical healing rates under each treatment strategy, a total of 194 events per group were required to obtain 80% power to reject the null hypothesis H0: log(HR_EBC/2LB_) ≥ log(1.33) in a one-tailed test of size 2.5%. Assuming healing times follow an exponential distribution with median healing time of 2.3 months (implying a rate parameter of log(2)/2.3 and cumulative incidence of healing of ~95.8% at 12 months in the absence of any loss to follow-up), an average follow-up duration of 12 months, and 10% loss to follow-up (i.e., conservatively assuming that 10% of the participants expected to heal during the follow-up period, would be lost to follow-up prior to these healing events being observed), 225 participants per group were required. Under identical assumptions about the distribution of healing times in the EBC group, the average follow-up duration, and proportion lost to follow-up, 225 participants to the CW group provides more than 80% power to reject the null hypothesis H0: log(HR_CW/EBC_) = 0 in a two-tailed test of size 5% if the hazard of healing were truly 33% higher in the CW group compared with the EBC group. We therefore aimed to recruit 225 participants to each of the three groups (675 participants in total). Note that the above sample size calculations made no adjustment for multiple comparisons in line with the perspective advanced in Molloy and colleagues [28].

Statistical analyses were conducted using Stata/MP v18.0 following a pre-specified analysis plan prepared prior to the completion of data collection (See Supporting information) [29,30]. No interim analyses were planned or conducted.

Baseline data, and data on compression use over time were analysed descriptively and graphically.

The primary analyses included both noninferiority and superiority comparisons. In each case, treatment effects are summarised in terms of cause-specific HRs (hence, participants that died prior to reference ulcer healing had their follow-up times censored on the date that they died). In line with recent guidance [31], for the noninferiority comparison (2LB versus EBC), two treatment effect estimands were targeted, whereby three pre-specified pre-healing intercurrent events ((1) failure to receive allocated treatment within 14 days of randomisation, (2) complete cessation of all compression for >7 days, or (3) receipt of treatments to close/remove incompetent veins) were handled under either a hypothetical strategy, or a treatment policy strategy [32]. For the superiority comparisons, these intercurrent events were handled under a treatment policy strategy only. Precise details of the treatment effect estimands targeted as part of the primary analyses are provided in Table B in S1 File.

For the comparison of 2LB and EBC handling intercurrent events under a hypothetical strategy, healing times were censored at the earliest time that any intercurrent event occurred, with time-varying stabilised inverse probability of censoring weights used to mitigate against potential bias due to this artificial censoring under the assumption of no confounding between the intercurrent events and healing conditional on the variables used to estimate the weights [33,34]. For the comparison adopting a treatment policy strategy, a modified intention-to-treat analysis was undertaken, with all available healing time data used. In both cases, the point estimates and 95% confidence intervals for the cause-specific HRs were obtained via Cox proportional hazards regression models, with baseline reference ulcer area and duration, participant age and mobility, and recruitment site included as covariates. For the superiority comparisons, the estimated HRs were obtained using the same model as outlined above. A pre-specified exploratory subgroup analysis was undertaken, to investigate treatment effect heterogeneity by baseline Margolis Index score [35]. Note that all analyses of the healing time outcomes used complete cases and assumed that censoring due to participants being lost to follow-up was noninformative conditional on the covariates included in the relevant analysis models.

The secondary reference ulcer healing time outcomes were analysed in an identical manner to the primary outcome. Time to healing of the reference leg was analysed in a similar manner to the reference ulcer healing superiority comparisons. Time to recurrence was analysed using similar methods but included only participants that had complete healing of the reference leg reported. The cumulative incidence of key clinical events (ulcer deterioration, skin deterioration, ulcer infection, and occurrence of new ulcers) at 6- and 12-months was estimated using the competing risks extension of the Nelson-Aalen estimator of the cumulative hazard, with the estimated probabilities being contrasted in terms of risk ratios [36]. Differences in VEINES-QoL and VEINES-Sym scores at 3-, 6-, and 12-months post-randomisation were estimated using a linear mixed effect model, with fixed effects for treatment group, time point, treatment-by-time interactions, baseline score, age, and reference ulcer area and duration, and random intercepts for recruitment site, with correlation between repeated measurements handled via an unstructured covariance matrix.

### Patient and public involvement

People who have experienced a venous leg ulcer, those who have cared for patients and members of the wider public were involved throughout the VenUS 6 from its inception.

Patient and public contributors were included in our Trial Management Group and Trial Steering Committee membership, and a Patient and Public Advisory Group (PPAG) was also set up to facilitate broader patient input over the course of the trial.

PPAG members contributed to trial design and documentation development and advised on recruitment and retention methods. PPAG members also reviewed the process evaluation and clinical trial results, and provided input to planned dissemination activities, including how results should be disseminated, and which findings would be most important and relevant to patients.

## Results

**Fig 1:**
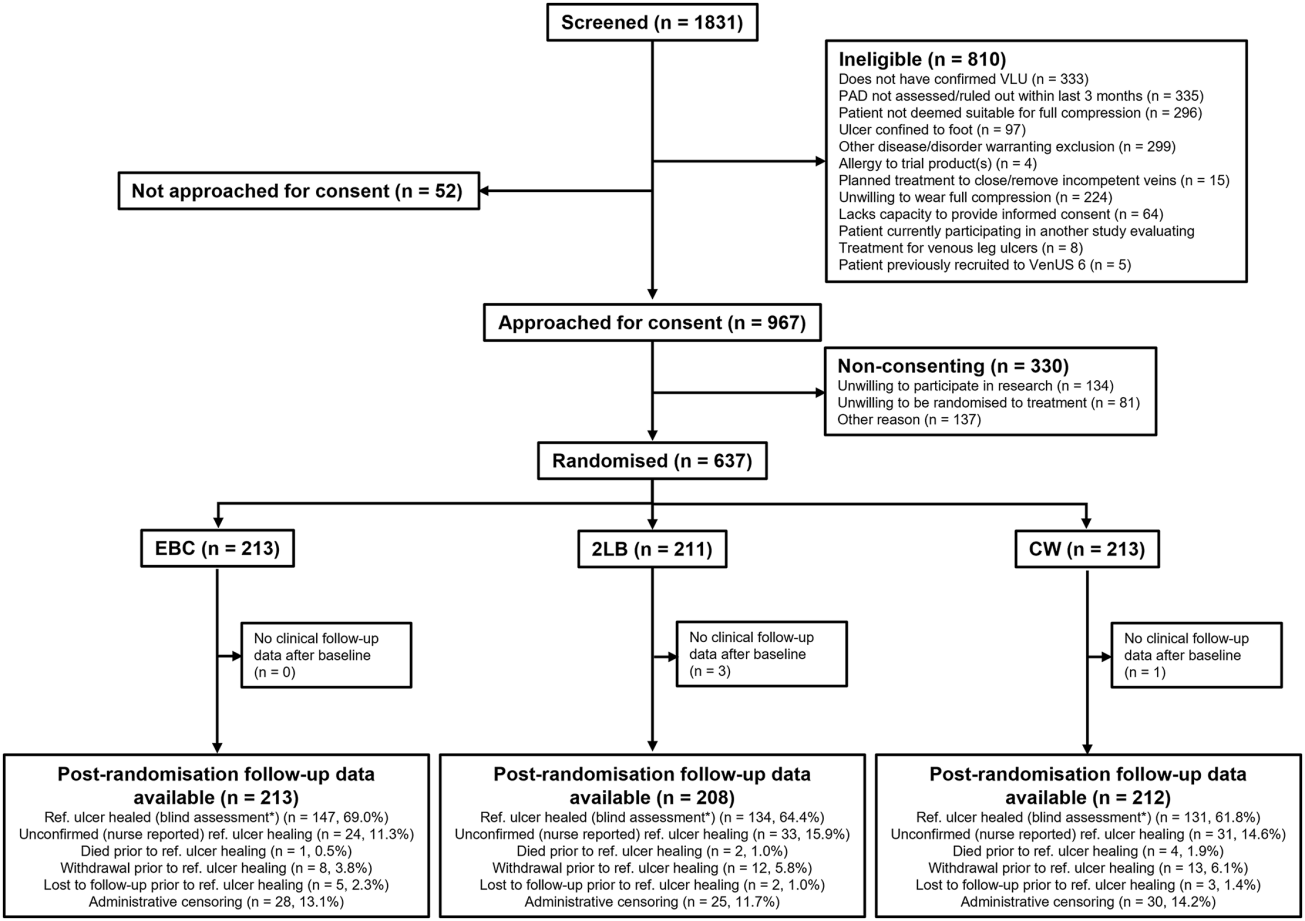
Trial profile. * There were 500 participants with nurse-reported healing of the reference ulcer (EBC = 171, 2LB = 167, CW = 162). Of these, 429 had at least one post-healing photograph of the reference ulcer taken to facilitate blinded assessment (EBC = 152, 2LB = 141, CW = 136), with 412 participants being assessed as healed by the blinded outcome assessors (EBC = 147, 2LB = 134, CW = 131). Legend: EBC, evidence-based compression; 2LB, two-layer bandage; CW, compression wraps; VLU, venous leg ulcer.

Between 03.02.2021 and 29.04.2024, 637 patients were randomly assigned to be offered CW (*n* = 213), 2LB (*n* = 211), or EBC (*n* = 213) (Fig 1). Participants were followed up between 03.03.2021 and 31.08.2024. Key baseline characteristics were similar across groups (Table 1).

**Table 1:** Baseline characteristics (all randomised participants).

	EBC (*N* = 213)	2LB (*N* = 211)	CW (*N* = 213)	Total (*N* = 637)
**Age (years)**				
*N*	213	211	213	637
Mean (Standard Deviation (SD))	71.4 (13.4)	69.6 (14.3)	69.7 (14.1)	70.3 (13.9)
Median (Q1, Q3)	73.9 (64.0, 81.7)	72.0 (61.8, 80.7)	73.0 (62.1, 79.8)	73.0 (62.3, 80.5)
Min, Max	27.6, 97.0	24.6, 96.7	27.6, 93.6	24.6, 97.0
**Sex, *n* (%)**				
Male	112 (52.6)	116 (55.0)	123 (57.7)	351 (55.1)
Female	100 (46.9)	94 (44.5)	88 (41.3)	282 (44.3)
Prefer not to say	1 (0.5)	0 (0.0)	0 (0.0)	1 (0.2)
Missing	0 (0.0)	1 (0.5)	2 (0.9)	3 (0.5)
**Ethnicity, *n* (%)**				
White	202 (94.8)	202 (95.7)	202 (94.8)	606 (95.1)
Mixed	0 (0.0)	0 (0.0)	2 (0.9)	2 (0.3)
Asian	6 (2.8)	2 (0.9)	3 (1.4)	11 (1.7)
Black	2 (0.9)	4 (1.9)	3 (1.4)	9 (1.4)
Chinese	1 (0.5)	0 (0.0)	0 (0.0)	1 (0.2)
Other	2 (0.9)	1 (0.5)	1 (0.5)	4 (0.6)
Missing	0 (0.0)	2 (0.9)	2 (0.9)	4 (0.6)
**Body mass index (kg/m²)**				
*N*	205	205	207	617
Mean (SD)	31.4 (8.5)	32.7 (9.5)	33.9 (10.1)	32.7 (9.4)
Median (Q1, Q3)	30.0 (25.1, 36.3)	30.1 (25.5, 37.4)	32.0 (26.8, 39.1)	30.6 (25.6, 37.6)
Min, Max	17.9, 61.6	14.4, 68.4	16.1, 73.2	14.4, 73.2
**Mobility, *n* (%)**				
Walks freely	123 (57.7)	128 (60.7)	131 (61.5)	382 (60.0)
Walks with difficulty	89 (41.8)	79 (37.4)	77 (36.2)	245 (38.5)
Immobile	1 (0.5)	4 (1.9)	3 (1.4)	8 (1.3)
Missing	0 (0.0)	0 (0.0)	2 (0.9)	2 (0.3)
**Diabetes, *n* (%)**				
Yes	47 (22.1)	49 (23.2)	53 (24.9)	149 (23.4)
No	166 (77.9)	162 (76.8)	158 (74.2)	486 (76.3)
Missing	0 (0.0)	0 (0.0)	2 (0.9)	2 (0.3)
**Ankle mobility, *n* (%)**				
Full range of motion	160 (75.1)	143 (67.8)	153 (71.8)	456 (71.6)
Reduced range of motion	52 (24.4)	65 (30.8)	51 (23.9)	168 (26.4)
Ankle fixed	1 (0.5)	3 (1.4)	7 (3.3)	11 (1.7)
Missing	0 (0.0)	0 (0.0)	2 (0.9)	2 (0.3)
**Ankle circumference (cm)**				
*N*	213	211	211	635
Mean (SD)	25.3 (3.5)	25.5 (3.3)	26.0 (4.4)	25.6 (3.8)
Median (Q1, Q3)	24.8 (22.9, 27.0)	25.0 (23.0, 27.5)	25.0 (23.1, 28.0)	25.0 (23.0, 27.5)
Min, Max	19.0, 41.0	19.0, 38.0	18.0, 56.0	18.0, 56.0
**VEINES-QoL Score (0–100**^**a**^)				
*N*	213	208	211	632
Mean (SD)	53.1 (23.8)	52.1 (24.8)	51.8 (24.4)	52.4 (24.3)
Median (Q1, Q3)	50.3 (35.4, 73.2)	53.6 (31.8, 72.1)	52.1 (32.7, 69.8)	52.5 (33.3, 71.9)
Min, Max	5.8, 100.0	3.3, 98.3	0.9, 95.8	0.9, 100.0
**VEINES-Sym Score (0–100**^**a**^)				
*N*	200	194	202	596
Mean (SD)	58.2 (24.4)	58.2 (25.7)	59.6 (24.2)	58.7 (24.7)
Median (Q1, Q3)	61.5 (39.0, 76.6)	62.4 (39.5, 76.5)	62.2 (41.7, 78.0)	62.0 (39.5, 76.8)
Min, Max	6.7, 100.0	0.0, 100.0	0.0, 100.0	0.0, 100.0
**Number of previous episodes of ulceration on ref. leg**				
*N*	212	207	211	630
Mean (SD)	1.3 (2.9)	1.4 (2.4)	1.4 (2.8)	1.3 (2.7)
Median (Q1, Q3)	0.0 (0.0, 1.0)	0.0 (0.0, 2.0)	0.0 (0.0, 2.0)	0.0 (0.0, 1.0)
Min, Max	0.0, 25.0	0.0, 15.0	0.0, 20.0	0.0, 25.0
**Time since first ever venous leg ulcer (months)**				
*N*	213	210	211	634
Mean (SD)	59.6 (117.1)	67.5 (117.3)	50.7 (90.9)	59.2 (109.2)
Geometric Mean (Geometric SD)	13.1 (6.5)	14.7 (6.8)	13.6 (5.9)	13.8 (6.4)
Median (Q1, Q3)	12.0 (3.0, 60.0)	12.0 (3.0, 79.0)	12.0 (3.1, 60.0)	12.0 (3.0, 60.0)
Min, Max	0.3, 840.0	0.5, 606.0	0.2, 600.0	0.2, 840.0
**Oldest ulcer on ref. leg (months)**				
*N*	212	207	211	630
Mean (SD)	10.8 (24.9)	17.9 (48.7)	11.8 (38.9)	13.5 (38.7)
Geometric Mean (Geometric SD)	4.1 (3.5)	5.2 (4.0)	3.9 (3.5)	4.3 (3.7)
Median (Q1, Q3)	3.0 (2.0, 8.0)	4.0 (2.0, 12.0)	3.5 (1.8, 7.0)	4.0 (2.0, 8.0)
Min, Max	0.3, 228.0	0.1, 372.0	0.2, 504.0	0.1, 504.0
**Reference ulcer duration (months)**				
*N*	213	211	211	635
Mean (SD)	10.1 (24.6)	11.4 (32.3)	8.4 (17.1)	10.0 (25.4)
Geometric Mean (Geometric SD)	3.6 (3.6)	4.0 (3.6)	3.4 (3.5)	3.7 (3.6)
Median (Q1, Q3)	3.0 (1.5, 7.0)	3.1 (1.6, 8.0)	3.2 (1.5, 7.0)	3.1 (1.5, 7.0)
Min, Max	0.2, 228.0	0.1, 372.0	0.1, 120.0	0.1, 372.0
**Reference ulcer area (cm²)**				
*N*	213	211	211	635
Mean (SD)	22.5 (51.3)	24.8 (50.7)	27.3 (55.0)	24.8 (52.3)
Geometric Mean (Geometric SD)	7.5 (4.4)	7.3 (4.9)	8.7 (4.5)	7.8 (4.6)
Median (Q1, Q3)	7.9 (3.0, 18.9)	8.0 (2.2, 20.0)	7.6 (3.2, 25.2)	7.9 (2.8, 20.0)
Min, Max	0.1, 396.0	0.1, 319.0	0.2, 396.0	0.1, 396.0
**Current compression treatment (prior to randomisation), *n* (%)**				
Four-layer compression bandage	16 (7.5)	6 (2.8)	6 (2.8)	28 (4.4)
Elastic two-layer bandage	39 (18.3)	39 (18.5)	39 (18.3)	117 (18.4)
Reduced compression bandaging	9 (4.2)	9 (4.3)	12 (5.6)	30 (4.7)
Short stretch bandage	18 (8.5)	27 (12.8)	27 (12.7)	72 (11.3)
Other compression bandage	0 (0.0)	3 (1.4)	2 (0.9)	5 (0.8)
Two-layer compression hosiery	21 (9.9)	27 (12.8)	24 (11.3)	72 (11.3)
Single layer compression hosiery	12 (5.6)	14 (6.6)	13 (6.1)	39 (6.1)
Other compression hosiery	1 (0.5)	4 (1.9)	5 (2.3)	10 (1.6)
Compression wraps (hook/loop Velcro)	3 (1.4)	2 (0.9)	11 (5.2)	16 (2.5)
Other compression wrap	0 (0.0)	1 (0.5)	1 (0.5)	2 (0.3)
Other compression treatment	2 (0.9)	1 (0.5)	0 (0.0)	3 (0.5)
Not currently receiving compression	90 (42.3)	77 (36.5)	72 (33.8)	239 (37.5)
Missing	2 (0.9)	1 (0.5)	1 (0.5)	4 (0.6)

a Higher scores indicate better disease specific quality of life and less severe symptoms.

Legend: EBC, evidence-based compression; 2LB, two-layer bandage; CW, compression wraps; SD, standard deviation.

**Fig 2:**
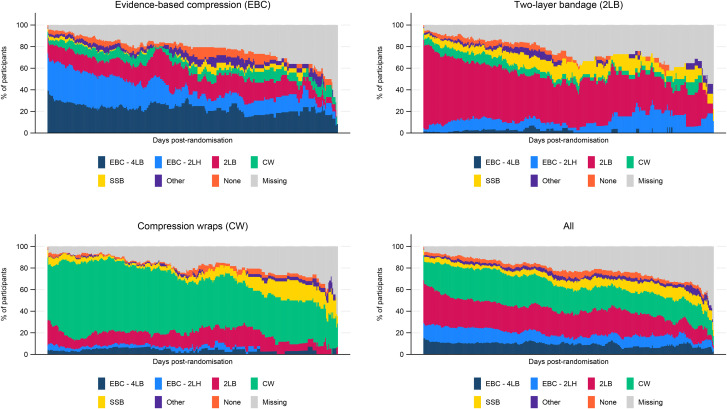
Compression treatment receipt over time by allocation and overall. Legend: EBC, evidence-based compression; 4LB, four-layer bandage; 2LH, two-layer hosiery; 2LB, two-layer bandage; CW, compression wraps; SSB, short stretch bandage.

As is common in clinical practice, many participants switched compression treatments from the one allocated at some point between randomisation and healing, with 455 (71%) participants receiving a compression treatment different from the one they were allocated at some point during follow-up, although patterns of treatment receipt were generally similar across the three groups (see Figs 2 and A in S1 File). Despite the delays in procurement and treatment switches, 498 (95%) of the 522 participants who completed the 1-month postal follow-up reported current or previous use of the allocated treatment.

**Fig 3:**
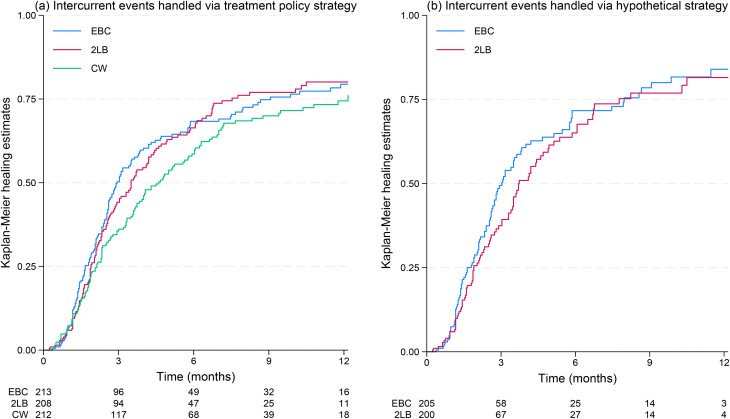
Time to blind assessed reference ulcer healing (Kaplan–Meier healing estimates by allocation)^a^. ^a^Note that the estimand handling intercurrent events under a hypothetical strategy is only estimated for the noninferiority comparison of EBC and 2LB. Legend: EBC, evidence-based compression; 2LB, two-layer bandage; CW, compression wraps.

Of the 637 randomised participants, 633 had available post-randomisation clinical follow-up data, with 412 (64.7% of randomised) having healing confirmed by blinded assessment. Cumulative incidences of healing at 12 months post-randomisation (based on Kaplan–Meier estimates) were 79.4%, 80.1%, and 76.2% in the EBC, 2LB, and CW groups, respectively. Figure 3(a) and 3(b) show the Kaplan–Meier failure (healing) functions for the healing time data used for the noninferiority comparison handling intercurrent events under a hypothetical strategy. For both analysis sets, there was limited evidence of important departures from proportional hazards (see Table C in S1 File). The estimated HRs for the noninferiority comparisons of EBC and 2LB were HR 1.01 (95% CI [0.79, 1.28]) and HR 1.16 (95% CI [0.86, 1.58]) for the analyses handling intercurrent events under the treatment policy and hypothetical strategies, respectively (Table 2). Since the pre-specified noninferiority margin (HR = 1.33) was less than the upper limit of the interval estimate for the estimand handling intercurrent events under a hypothetical strategy, the hypothesis that 2LB is inferior to EBC cannot be rejected (in line with our pre-specified analysis plans), despite the apparent similarity in healing rates across these two groups. Rates of healing were seemingly slower in the CW group compared with both the EBC group (HR = 0.78 (95% CI [0.61, 1.00], *p* = 0.046)) and the 2LB group (HR = 0.79 (95% CI [0.61, 1.01], *p* = 0.056)) (Tables 2; Table D and Fig B in S1 File with the 95% CI intervals ranging from a 39% reduction in to the hazard of healing to there being no difference between groups.

**Table 2:** Blind assessed reference ulcer healing comparisons.

Test	Estimand	Estimate^a^ (95% CI^b^)
Noninferiority	Cause-specific hazard ratio (HR) comparing EBC vs. 2LB (intercurrent events handled under a treatment policy strategy via a modified intention-to-treat analysis)	1.01 (0.79, 1.28)
	Cause-specific HR comparing EBC vs. 2LB (intercurrent events handled under a hypothetical strategy by censoring healing times at the first occurrence of a relevant intercurrent event and applying inverse probability of censoring weights)	1.16 (0.86, 1.58)
Superiority	Cause-specific HR comparing CW vs. EBC (intercurrent events handled under a treatment policy strategy via a modified intention-to-treat analysis)	0.78 (0.61,1.00)
	Cause-specific HR comparing CW vs. 2LB (intercurrent events handled under a treatment policy strategy via a modified intention-to-treat analysis)	0.79 (0.61, 1.01)

^a^ All analyses conditioned on baseline reference ulcer area and duration, participant age and mobility status, and shared frailties for recruitment site.

^b^ 95% confidence intervals obtained via nonparametric bootstrapping (bias-corrected, 1,000 replicates) for the analysis using inverse probability of censoring weights. For all other comparisons, Wald method 95% confidence intervals based on the estimated variance-covariance matrix of the fitted model are reported.

Legend: EBC, evidence-based compression; 2LB, two-layer bandage; CW, compression wraps; VLU, venous leg ulcer.

Post-hoc analyses (suggested by a reviewer) of the primary endpoint data restricted to participants that were known to have received their allocated treatment for at least 50, 75, 90, or 95% of their follow-up period are provided in Fig C in S1 File and show similar findings to the planned primary analyses. The subgroup analysis did not find compelling evidence of treatment effect heterogeneity by baseline Margolis Index score (see Table E in S1 File).

Results for the secondary reference ulcer healing time outcomes show qualitatively similar patterns to those obtained for the primary outcome (Tables F–K and Figs D to G in S1 File, eFigures 4–7).

Similar rates of reference leg healing were observed in the 2LB and EBC groups (HR = 1.01, (95% CI [0.81, 1.26], *p* = 0.903)). The rates of healing were perhaps slightly lower in the CW group compared with the EBC group (HR = 0.87, (95% CI [0.70, 1.09], *p* = 0.232)) and the 2LB group (HR = 0.86, (95% CI [0.67, 1.08], *p* = 0.187)), although the data are also compatible with there being no difference in healing rates across the three groups (Tables L and M and Figs H and I in S1 File). Allocation to EBC was associated with the highest rate of ulcer recurrence following complete healing of the reference leg, with allocation to CW being associated with the lowest rate of recurrence. However, the data are also compatible with there being no associations between allocation and ulcer recurrence (Table N and Fig J in S1 File).

There were no statistically significant differences in cumulative incidence of ulcer infection, ulcer deterioration, skin deterioration, or new/incident ulceration over either 6- or 12-month time horizons (Table 3). However, the estimates are imprecise, with wide 95% confidence intervals that including potentially important differences between the trial arms.

**Table 3:** Incidence of clinical events (all randomised participants with any follow-up data available).

Event	Time horizon	EBC N (cumulative incidence*)	2LB N (cumulative incidence*)	CW N (cumulative incidence*)	CW vs. EBC Relative risk (95% CI)	CW vs. 2LB Relative risk (95% CI)
Ulcer infection	6 months	31 (15.2%)	38 (19.9%)	43 (21.1%)	1.40 (0.93, 2.10)	1.06 (0.73, 1.56)
	12 months	43 (23.5%)	44 (24.4%)	51 (26.0%)	1.11 (0.77, 1.58)	1.06 (0.75,1.51)
Ulcer deterioration	6 months	44 (21.9%)	43 (23.1%)	52 (25.8%)	1.18 (0.83, 1.67)	1.12 (0.78, 1.60)
	12 months	59 (31.5%)	52 (29.8%)	66 (34.5%)	1.10 (0.82,1.47)	1.16 (0.85, 1.59)
Skin deterioration	6 months	53 (26.2%)	43 (23.3%)	42 (21.0%)	0.80 (0.56, 1.14)	0.90 (0.6 2, 1.31)
	12 months	74 (39.8%)	52 (30.3%)	60 (32.5%)	0.82 (0.62, 1.08)	1.07 (0.78, 1.47)
Incident ulceration	6 months	24 (12.1%)	23 (12.5%)	27 (13.7%)	1.13 (0.68,1.87)	1.09 (0.65, 1.84)
	12 months	44 (25.0%)	31 (18.4%)	34 (18.6%)	0.7 (0.50,1.11)	1.02 (0.65, 1.59)

* Cumulative incidence estimated via the Nelson -Aalen estimator of the cumulative sub-hazard of the event of interest (with death as a competing event) without adjustment for any baseline covariates

Legend: EBC, evidence-based compression; 2LB, two-layer bandage; CW, compression wraps; VLU, venous leg ulcer.

Endovenous ablation, adjunct to compression therapy, has previously been reported as resulting in faster venous ulcer healing than compression alone [37]. During the study, only 23 (3.6%) VenUS 6 participants reported receiving this treatment during follow-up. There was limited evidence of differences between groups with regards to receipt of treatment to close/remove incompetent superficial veins, hospital admissions relating to the venous leg ulcer, or all-cause mortality (Table O in S1 File).

These clinical events were also considered to be adverse events. In total, nine serious adverse events were reported: unplanned hospitalisations (CW = 4), patient death (CW = 3, 2LB = 1), and occurrence of deep vein thrombosis (EBC = 1). Eight events were deemed unrelated or unlikely to be related to study treatment. One event (patient death) was deemed possibly related as the cause of death was unknown by the GP and so could not be provided at the time of study closure (2LB = 1).

There were no statistically significant differences in VEINES-QoL and VEINES-Sym scores at 3-, 6-, or 12-months post-randomisation (Tables P and Q in S1 File). Likewise, descriptive summaries showed no clear differences in ulcer related pain at any time point (Table R in S1 File).

## Discussion

VenUS 6 results suggest that offering people with venous leg ulcers treatment with CW may not improve healing times compared with the offer of other strong compression treatments tested. Compared with EBC, CW is estimated to reduce the rate of ulcer healing by 22%. However, confidence intervals suggest comparative reductions in healing rates for CW compared with EBC as high as 39% or as low as 0% meaning uncertainty remains regarding the magnitude of effect. Overall, rates of healing for the EBC and 2LB seem similar but there is also uncertainty for this comparison, with the finding falling just outside standard alpha value of 0.05 (*p* = 0.056) and the data being compatible with clinically relevant treatment effects in either direction.

The use of compression for people with venous leg ulcers remains important. The results of this study support offering EBC or 2LB as the first line therapy choice. This corresponds to results from previous studies which suggested that 4LB and 2LH (EBC) confer similar healing times [13], and 2LB have the highest probability of being clinically effective [15]. The underpinning evidence for 2LB in this previous work was of low or very low quality and findings were uncertain necessitating the need for more robust research [15] as has been provided by this study.

Prior to this research, there were only two small RCTs of CW (*n* = 78 participants) and neither reported time to ulcer healing [19,20]. VenUS 6 has therefore provided evidence in relation to use of CW for venous leg ulcers. As CW seems unlikely to reduce the time to healing when compared to these alternate compression therapies, they may not be an optimal first line treatment to promote venous leg ulcer healing.

All the compression therapies evaluated in VenUS 6 have the same underlying mechanisms of actions, which is to apply strong compression (aiming for 40 mmHg of compression at the ankle). Possible differences in outcome are then potentially related to application of and adherence to the various compression treatments. CWs and 2LH are therapies that can be removed and reapplied by patients and informal carers between clinical appointments. Unlike 2LH, CW also requires the user to tighten the wrap to an appropriate level if reapplied. The level of removal frequency and re-application is one mechanism that may explain the findings observed. Repeated removal or loosening could reduce the overall dose of compression received which may explain why ulcers in this arm of the trial were, on average, slower to heal. These issues will be explored in an accompanying process evaluation to further explain the study findings.

Endovenous ablation, adjunct to compression therapy, results in faster venous ulcer healing than compression alone [37], however a limited number (23, 3.6%) of VenUS 6 participants were reported to have received this treatment during follow-up. The rate of receipt observed in this study is likely to have been reduced because of UK SARS-CoV-2 (COVID-19) restrictions and NHS pressures. Compression therapy, therefore, remains a key treatment for this population.

VenUS 6 is a robust clinical trial comparing the effectiveness of proven compression therapies with CW and 2LB to heal venous leg ulcers. It is the largest treatment trial for venous leg ulcers that has been conducted internationally and builds on the previous VenUS I and IV trials to give the most comprehensive primary data-derived insights to date on the relative effects of key compression therapies to treat venous leg ulcers to date [13,23]. Prior to VenUS 6, trial-derived data on CW and the 2LB were limited with large uncertainty that impacted decision-making [13]. Features such as rigorous generation of randomised groups, use of blinded outcome assessment for the primary outcome and limited attrition of primary outcome data all minimise the risk of bias of study findings. The multicentre nature of the study with over 30 sites, the broad eligibility criteria and pragmatic approach mean the findings are highly relevant to practice. The study itself does not consider the totality of existing evidence, however, a network meta-analysis to assess this has been undertaken which will be published separately, along with cost-effectiveness analyses.

In VenUS 6, flexible follow-up was used to maximise study recruitment, meaning some participants did not complete outcome measurements at the later time points. This then reduced available data for comparisons of the healing time endpoints, and the longer-term secondary outcome analysis. Following established practices used in other UK venous leg ulcer studies, outcomes were collected using postal and telephone methods. Compared to face-to-face data collection this may have impacted on response rates; however, this was the most feasible approach to data collection given the participant numbers required, with a range of strategies included to encourage engagement.

The incidence rates of ulcer healing were lower than the rates assumed in the sample size calculations. This coupled with slight under-recruitment, meant that the overall number of healing events contributing to the analysis was substantially smaller than the number required to attain 80% power. This was further compounded by substantial amounts of treatment switching and delays in receipt of the allocated treatment. Because of this treatment switching, results should be interpreted as estimates of the comparative effectiveness of allocation to the treatment strategies under the patterns of adherence observed in the trial, rather than estimates of the effectiveness of the treatments under ideal settings. Treatment switching was also common in VenUS IV and, like this study, VenUS 6 is a pragmatic trial where changes to treatment reflects normal practice, ensuring the high external validity of the study.

We did not specify definitions for clinical secondary outcomes (e.g., ulcer deterioration) as these assessments are conducted routinely as part of standard clinical care. As a result, there may have been minor nuances in the operational definition between sites; however we anticipate that the core principles of definition and review were the same across all sites.

Study recruitment commenced following the peak of the COVID-19 pandemic restrictions, delays to commencement of recruitment at some sites were experienced. We sought to minimise and mitigate the impacts of this by using agile trial management to support recruiting sites.

In conclusion, compression remains an important treatment for patients with venous leg ulcers. CW is unlikely to reduce the time to healing when compared to other compression treatments. The results of VenUS 6 give confidence in using EBC or 2LB as first line treatment options, despite some remaining uncertainty regarding the relative effects of CW and 2LB in comparison to the EBC strategy.

### Data sharing

Anonymised datasets generated and analysed during the current study, and the code used in the analysis can be accessed via a publicly available open research repository (**DOI: https://doi.org/10.17605/OSF.IO/EM4AP**). Sharing of this anonymised data is covered by original participant consent for VenUS 6 which permits sharing of data to support future research via sharing anonymously.

## Supporting information

S1 File Supplementary tables and figures. **Table A**: Specification of compression treatment modalities. **Table B**: Treatment effect estimands targeted as part of the primary analyses. **Table C**: Primary outcome - tests of proportional hazards assumption for effects of allocation. **Table D**: Primary outcome (modified ITT analysis set) – sample averaged differences in cumulative incidence of healing at 1, 3, 6, and 12 months. **Table E**: Primary outcome (modified ITT analysis set) – baseline Margolis index subgroup analysis (p-value for test of interaction = 0.619). **Table F**: Time to blind assessed or nurse reported ulcer healing – tests of proportional hazards assumption for treatment effects. **Table G**: Time to blind assessed or nurse reported ulcer healing – estimated hazard ratios. **Table H**: Time to blind assessed or nurse reported ulcer healing (modified ITT analysis set) – sample averaged differences in cumulative incidence of healing at 1, 3, 6, and 12 months. **Table I**: Time to nurse reported ulcer healing – tests of proportional hazards assumption for treatment effects. **Table J**: Time to nurse reported ulcer healing – estimated hazard ratios. **Table K**: Time to nurse reported ulcer healing (modified ITT analysis set) – sample averaged differences in cumulative incidence of healing at 1, 3, 6, and 12 months. **Table L**: Time to reference leg healing (nurse reported) – estimated hazard ratios. **Table M**: Time to reference leg healing (nurse reported) - sample averaged differences in cumulative incidence of healing at 1, 3, 6, and 12 months. **Table N**: Time to ulcer recurrence – Estimated hazard ratios. **Table O**: Cumulative incidence of key clinical events by 6 and 12 months by allocation. **Table P**: VEINES QoL - estimated differences in expected score. **Table Q**: VEINES Sym - estimated differences in expected score. **Table R**: Ulcer related pain score (0–100, higher scores = greater pain) - all participants (participants reporting no ulcers imputed with score of zero). **Fig A**: Receipt of allocated compression treatment over time. **Fig B**: Primary outcome (modified ITT analysis set) – sample averaged healing functions by allocation and differences in median healing times. **Fig C**: KM plots of healing times from exploratory analyses using only participants with ≥50%, ≥ 75%, ≥ 90% or ≥95% of their follow-up time using the allocated treatment. **Fig D**: Time to blind assessed or nurse reported ulcer healing – Kaplan–Meier healing estimates by allocation^a^. **Fig E**: Time to blind assessed or nurse reported ulcer healing (modified ITT analysis set) – sample averaged healing functions by allocation and differences in median healing times. **Fig F**: Time to nurse reported ulcer healing – Kaplan–Meier healing estimates by allocation^a^. **Fig G**: Time to nurse reported ulcer healing (modified ITT analysis set) – sample averaged healing functions by allocation and differences in median healing times. **Fig H**: Time to reference leg healing (nurse reported) – Kaplan–Meier healing estimates by allocation. **Fig I**: Time to reference leg healing (nurse reported) - sample averaged healing functions by allocation and differences in median healing times. **Fig J**: Time to ulcer recurrence (nurse reported) among participants with complete healing of reference leg - Kaplan–Meier failure estimates by allocation.
(DOCX)

S2 File CONSORT checklist for abstracts. (DOCX)

S3 File CONSORT checklist. This is an Open Access article distributed under the terms of the Creative Commons Attribution License (https://creativecommons.org/licenses/by/4.0/), which permits unrestricted use, distribution, and reproduction in any medium, provided the original work is properly cited. *We strongly recommend reading this statement in conjunction with the CONSORT 2025 Explanation and Elaboration and/or the CONSORT 2025 Expanded Checklist for important clarifications on all the items. We also recommend reading relevant CONSORT extensions. See www.consort-spirit.org. Checklist as completed obtained via www.consort-spirit.org.
(DOCX)

S4 File CONSERVE CONSORT extension. (DOCX)

S5 File VenUS 6 protocol (v1.6 17.07.2023). (PDF)

S6 File VenUS 6 statistical analysis plan v1.0 24.11.2023. (PDF)
