# Supplementary material for: Compression therapies for venous leg ulcers: The VENous Ulcer Study 6 (VenUS 6), an open, multicentre, randomised clinical trial
Source: PLoS Med. 2026 Jul 10;23(7):e1005154. doi: 10.1371/journal.pmed.1005154 (PMC13354008; doi:10.1371/journal.pmed.1005154)
Supplement: S1 File — Table A: Specification of compression treatment modalities. Table B: Treatment effect estimands targeted as part of the primary analyses. Table C: Primary outcome - tests of proportional hazards assumption for effects of allocation. Table D: Primary outcome (modified ITT analysis set) – sample averaged differences in cumulative incidence of healing at 1, 3, 6, and 12 months. Table E: Primary outcome (modified ITT analysis set) – baseline Margolis index subgroup analysis (p-value for test of interaction = 0.619). Table F: Time to blind assessed or nurse reported ulcer healing – tests of proportional hazards assumption for treatment effects. Table G: Time to blind assessed or nurse reported ulcer healing – estimated hazard ratios. Table H: Time to blind assessed or nurse reported ulcer healing (modified ITT analysis set) – sample averaged differences in cumulative incidence of healing at 1, 3, 6, and 12 months. Table I: Time to nurse reported ulcer healing – tests of proportional hazards assumption for treatment effects. Table J: Time to nurse reported ulcer healing – estimated hazard ratios. Table K: Time to nurse reported ulcer healing (modified ITT analysis set) – sample averaged differences in cumulative incidence of healing at 1, 3, 6, and 12 months. Table L: Time to reference leg healing (nurse reported) – estimated hazard ratios. Table M: Time to reference leg healing (nurse reported) - sample averaged differences in cumulative incidence of healing at 1, 3, 6, and 12 months. Table N: Time to ulcer recurrence – Estimated hazard ratios. Table O: Cumulative incidence of key clinical events by 6 and 12 months by allocation. Table P: VEINES QoL - estimated differences in expected score. Table Q: VEINES Sym - estimated differences in expected score. Table R: Ulcer related pain score (0–100, higher scores = greater pain) - all participants (participants reporting no ulcers imputed with score of zero). Fig A: Receipt of allocated compression treatment over time. Fig B: [file pmed.1005154.s001.docx]

S1 File: Supplementary Tables and Figures

**Tables**

[**Table A: Specification of compression treatment modalities** 3](#_Toc220682392)

[**Tab****le B: Treatment effect estimands targeted as part of the primary analyses** 3](#_Toc220682393)

[**Table C: Primary outcome - tests of proportional hazards assumption for effects of allocation** 6](#_Toc220682394)

[**Table D: Primary outcome (modified ITT analysis set) – sample averaged differences in cumulative incidence of healing at 1, 3, 6 and 12 months** 7](#_Toc220682395)

[**Table E: Primary outcome (modified ITT analysis set) – baseline Margolis index subgroup analysis (p-value for test of interaction = 0·619)** 8](#_Toc220682396)

[**Table F: Time to blind assessed or nurse reported ulcer healing – tests of proportional hazards assumption for treatment effects** 9](#_Toc220682397)

[**Table G: Time to blind assessed or nurse reported ulcer healing – estimated hazard ratios** 9](#_Toc220682398)

[**Table H: Time to blind assessed or nurse reported ulcer healing (modified ITT analysis set) – sample averaged differences in cumulative incidence of healing at 1, 3, 6 and 12 months** 11](#_Toc220682399)

[**Table I: Time to nurse reported ulcer healing – tests of proportional hazards assumption for treatment effects** 12](#_Toc220682400)

[**Table J: Time to nurse reported ulcer healing – estimated hazard ratios** 12](#_Toc220682401)

[**Table K: Time to nurse reported ulcer healing (modified ITT analysis set) – sample averaged differences in cumulative incidence of healing at 1, 3, 6 and 12 months** 13](#_Toc220682402)

[**Table L: Time to reference leg healing (nurse reported) – estimated hazard ratios** 14](#_Toc220682403)

[**Table M: Time to reference leg healing (nurse reported) - sample averaged differences in cumulative incidence of healing at 1, 3, 6 and 12 months** 15](#_Toc220682404)

[**Table N: Time to ulcer recurrence – Estimated hazard ratios** 16](#_Toc220682405)

[**Table O: Cumulative incidence of key clinical events by 6 and 12 months by allocation** 16](#_Toc220682406)

[**Table P: VEINES QoL - estimated differences in expected score** 17](#_Toc220682407)

[**Table Q: VEINES Sym - estimated differences in expected score** 17](#_Toc220682408)

**Table R: Ulcer related pain score (0 - 100, higher scores = greater pain) - all participants (participants reporting no ulcers imputed with score of zero)…………………………………………………..**18

**Figures**

**[Fig A: Receipt of allocated compression treatment over time](#_Toc204615360)** [5](#_Toc204615360)

[**Fig B: Primary outcome (modified ITT analysis set) – sample averaged healing functions by allocation and differences in median healing times** 7](#_Toc204615362)

**Fig C: KM plots of healing times from exploratory analyses using only participants with ≥50%, ≥75%, ≥90% or ≥95% of their follow-up time using the allocated treatment…………………………………**8

[**Fig D: Time to blind assessed or nurse reported ulcer healing – Kaplan-Meier healing estimates by allocation^a^** 9](#_Toc204615363)

[**Fig E: Time to blind assessed or nurse reported ulcer healing (modified ITT analysis set) – sample averaged healing functions by allocation and differences in median healing times** 10](#_Toc204615364)

[**Fig F: Time to nurse reported ulcer healing – Kaplan-Meier healing estimates by allocation^a^** 11](#_Toc204615365)

[**Fig G: Time to nurse reported ulcer healing (modified ITT analysis set) – sample averaged healing functions by allocation and differences in median healing times** 13](#_Toc204615366)

[**Fig H: Time to reference leg healing (nurse reported) – Kaplan-Meier healing estimates by allocation** 14](#_Toc204615367)

[**Fig I: Time to reference leg healing (nurse reported) - sample averaged healing functions by allocation and differences in median healing times** 15](#_Toc204615368)

[**Fig J: Time to ulcer recurrence (nurse reported) among participants with complete healing of reference leg - Kaplan-Meier failure estimates by allocation** 16](#_Toc204615369)

**Table A: Specification of compression treatment modalities**

| **Treatment Modality** | **Specification** |
| --- | --- |
| *Compression Wraps* | - Adjustable compression sleeve secured with hook and loop (Velcro™) fastenings - Designed, and marketed to be worn on the lower leg and foot - Aimed to deliver >40mmHg of compression at the ankle (with a guidance system) - CE Marked - Available on United Kingdom National Health Service (Prescription |
|  | - Compression wraps marketed solely for treatment of lymphedema were not permitted to be used |
| *Two-Layer Bandage* | - Two-layer bandage kit system, consisting of an initial bandage layer covered with a top cohesive compression bandage - Aimed to deliver >40mmHg compression at the ankle - Systems included K-Two (Urgo), Coban 2, Andoflex and Actico2c - Other two-layer bandage kit systems were considered on a case by case basis in consultation with the Chief Investigator and Trial Manager |
| *Evidence Based Compression* | - Any four-layer bandage delivering >40mmHg compression at the ankle - Any recognised two-layer compression hosiery delivering sustained graduated compression of >40mmHg at the ankle including made to measure hosiery kits. |

**Table B: Treatment effect estimands targeted as part of the primary analyses**

| **Non-inferiority comparison of 2LB vs EBC handling specified intercurrent events under a hypothetical strategy** | |
| --- | --- |
| Population | Adult patients with at least one venous leg ulcer lying wholly or partially within the gaiter region of the leg, and with a negative assessment of peripheral arterial disease within three months of being assessed for eligibility |
| Treatment conditions | **Intervention**: allocation to 2LB regardless of subsequent departures from this treatment strategy except for the three intercurrent events described below  **Control**: allocation to EBC regardless of subsequent departures from this treatment strategy except for the three intercurrent events described below |
| Endpoint | Time to blind assessed reference ulcer healing with death or amputation of the leg prior to healing as a competing event |
| Summary measure | Cause-specific hazard ratio for reference ulcer healing (EBC/2LB) |
| Intercurrent events and strategies used to handle them | Death or amputation of the reference leg prior to healing handled via the endpoint definition and target summary measure. Failure to receive the allocated treatment within 14 days of randomisation, complete cessation of all compression treatment (prior to healing) for a period exceeding 7 days, and receipt of treatment(s) to close/remove incompetent superficial veins, handled under a hypothetical strategy. All other intercurrent events handled via a treatment policy strategy |
|  | |
| **Non-inferiority comparison of 2LB vs EBC handling all non-truncating intercurrent events under a treatment policy strategy** | |
| Population | Adult patients with at least one venous leg ulcer lying wholly or partially within the gaiter region of the leg, and with a negative assessment of peripheral arterial disease within three months of being assessed for eligibility |
| Treatment conditions | **Intervention**: allocation to 2LB regardless of subsequent departures from this treatment strategy  **Control**: allocation to EBC regardless of subsequent departures from this treatment strategy |
| Endpoint | Time to blind assessed reference ulcer healing with death or amputation of the leg prior to healing as a competing event |
| Summary measure | Cause-specific hazard ratio for reference ulcer healing (EBC/2LB) |
| Intercurrent events and strategies used to handle them | Death or amputation of the reference leg prior to healing handled via the endpoint definition and target summary measure. All other non-truncating intercurrent events handled via a treatment policy strategy |
|  | |
| **Superiority comparison of CW vs EBC handling all non-truncating intercurrent events under a treatment policy strategy** | |
| Population | Adult patients with at least one venous leg ulcer lying wholly or partially within the gaiter region of the leg, and with a negative assessment of peripheral arterial disease within three months of being assessed for eligibility |
| Treatment conditions | **Intervention**: allocation to CW regardless of subsequent departures from this treatment strategy  **Control**: allocation to EBC regardless of subsequent departures from this treatment strategy |
| Endpoint | Time to blind assessed reference ulcer healing with death or amputation of the leg prior to healing as a competing event |
| Summary measure | Cause-specific hazard ratio for reference ulcer healing (CW/EBC) |
| Intercurrent events and strategies used to handle them | Death or amputation of the reference leg prior to healing handled via the endpoint definition and target summary measure. All other non-truncating intercurrent events handled via a treatment policy strategy |
|  | |
| **Superiority comparison of CW vs 2LB handling all non-truncating intercurrent events under a treatment policy strategy** | |
| Population | Adult patients with at least one venous leg ulcer lying wholly or partially within the gaiter region of the leg, and with a negative assessment of peripheral arterial disease within three months of being assessed for eligibility |
| Treatment conditions | **Intervention**: allocation to CW regardless of subsequent departures from this treatment strategy  **Control**: allocation to 2LB regardless of subsequent departures from this treatment strategy |
| Endpoint | Time to blind assessed reference ulcer healing with death or amputation of the leg prior to healing as a competing event |
| Summary measure | Cause-specific hazard ratio for reference ulcer healing (CW/2LB) |
| Intercurrent events and strategies used to handle them | Death or amputation of the reference leg prior to healing handled via the endpoint definition and target summary measure. All other non-truncating intercurrent events handled via a treatment policy strategy |

*Legend: EBC – evidence-based compression; 2LB – two-layer bandage; CW – compression wraps*

**Fig A: Receipt of allocated compression treatment over time**


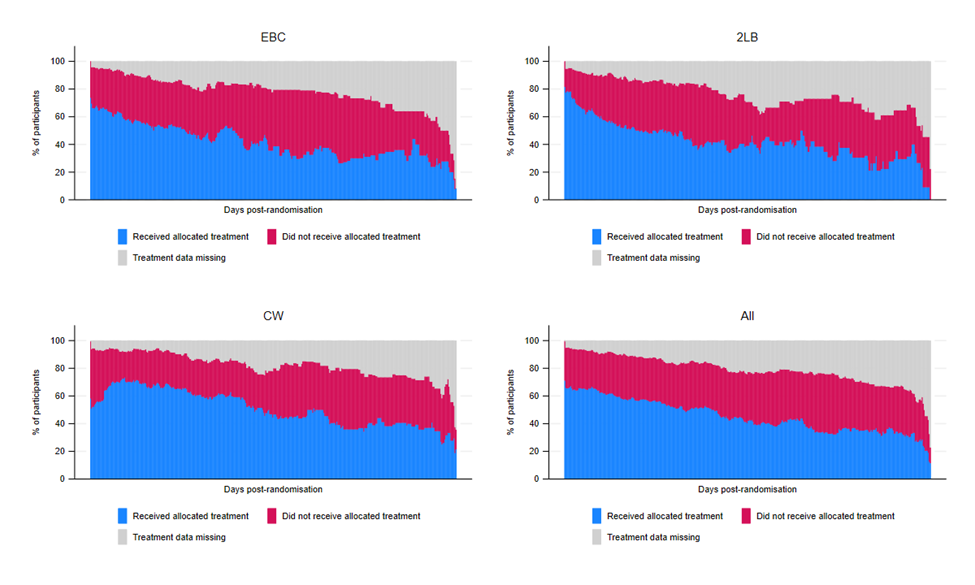


*Legend: EBC – evidence-based compression; 2LB – two-layer bandage; CW – compression wraps*

**Table C: Primary outcome - tests of proportional hazards assumption for effects of allocation**

| **Effect** | **p-value for test of proportional hazards** | | |
| --- | --- | --- | --- |
|  | **Time** | **Log(time)** | **Rank** |
| 2LB vs EBC (intercurrent events handled via treatment policy strategy) | 0·328 | 0·304 | 0·185 |
| 2LB vs EBC (intercurrent events handled via hypothetical strategy) | 0·514 | 0·620 | 0·396 |
| CW vs EBC (intercurrent events handled via treatment policy strategy) | 0·141 | 0·320 | 0·193 |
| CW vs 2LB (intercurrent events handled via treatment policy strategy) | 0·625 | 0·980 | 0·989 |

*Legend: EBC – evidence-based compression; 2LB – two-layer bandage; CW – compression wraps*

**Fig B: Primary outcome (modified ITT analysis set) – sample averaged healing functions by allocation and differences in median healing times**


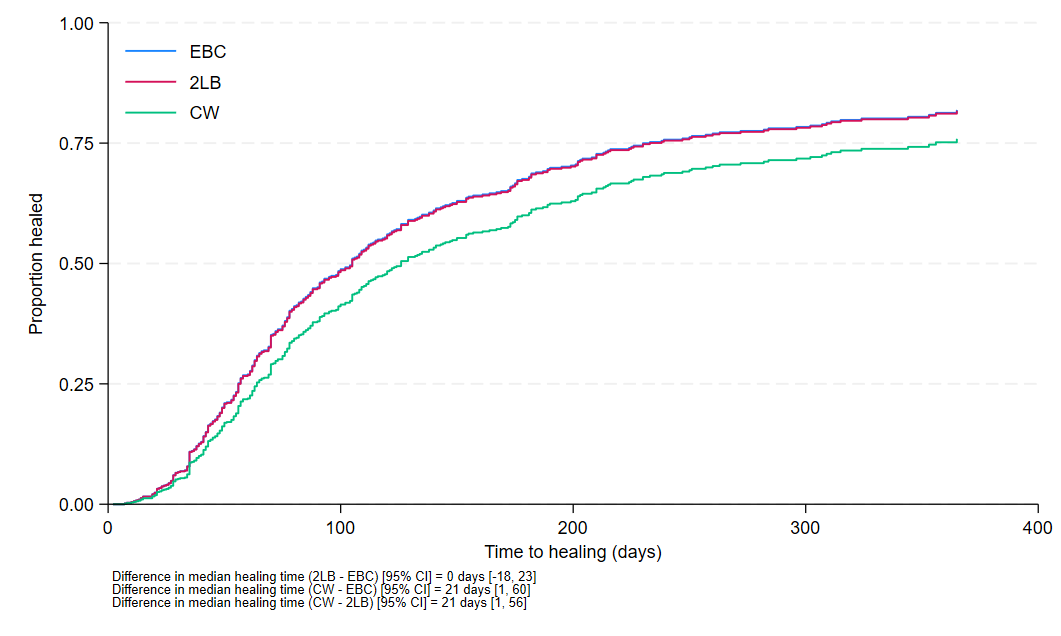


*Legend: EBC – evidence-based compression; 2LB – two-layer bandage; CW – compression wraps*

**Table D: Primary outcome (modified ITT analysis set) – sample averaged differences in cumulative incidence of healing at 1, 3, 6 and 12 months**

| **Time horizon** | **Contrast** | **Estimate [95% CI]** |
| --- | --- | --- |
| 1 month | Pr(Healed \| 2LB) - Pr(Healed \| EBC) | -0·0 5% [-1·82,1·75] |
|  | Pr(Healed \| CW) - Pr(Healed \| EBC) | -1·4 3% [-3·31, 0·24] |
|  | Pr(Healed \| CW) - Pr(Healed \| 2LB) | -1·3 8% [-3·04, 0·25] |
| 3 months | Pr(Healed \| 2LB) - Pr(Healed \| EBC) | -0·2 2% [-7·90, 7·17] |
|  | Pr(Healed \| CW) - Pr(Healed \| EBC) | -7·1 6% [-15·44, 0·74] |
|  | Pr(Healed \| CW) - Pr(Healed \| 2LB) | -6·9 3% [-14·61, 0·81] |
| 6 months | Pr(Healed \| 2LB) - Pr(Healed \| EBC) | -0·2 2% [-7·14, 6·56] |
|  | Pr(Healed \| CW) - Pr(Healed \| EBC) | -7·5 1% [-16·29,-0·08] |
|  | Pr(Healed \| CW) - Pr(Healed \| 2LB) | -7·2 9% [-15·27, -0·06] |
| 12 months | Pr(Healed \| 2LB) - Pr(Healed \| EBC) | -0·1 7% [-5·26, 4·80] |
|  | Pr(Healed \| CW) - Pr(Healed \| EBC) | -6·0 0% [-13·17, -0·82] |
|  | Pr(Healed \| CW) - Pr(Healed \| 2LB) | -5·8 4% [-12·34, -0·71] |

*Legend: EBC – evidence-based compression; 2LB – two-layer bandage; CW – compression wraps; ITT – intention to treat*

**Fig C: *KM plots of healing times from exploratory analyses using only participants with ≥50%, ≥75%, ≥90% or ≥95% of their follow-up time using the allocated treatment***


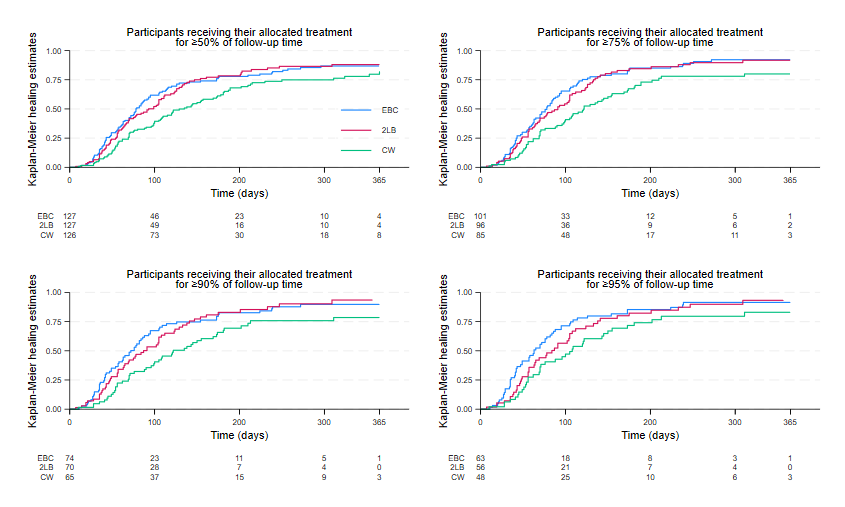


*Legend: EBC – evidence-based compression; 2LB – two-layer bandage; CW – compression wraps*

**Table E: Primary outcome (modified ITT analysis set) – baseline Margolis index subgroup analysis (p-value for test of interaction = 0·619)**

|  | **HR(2LB/EBC)** Estimate [95% CI] | **HR(CW/EBC)** Estimate (95% CI) | **HR(CW/2LB)** Estimate (95% CI) |
| --- | --- | --- | --- |
| Margolis Index = 0 | 1·11 [0·75, 1·63] | 0·80 [0·53, 1·20] | 0·72 [0·47, 1·11] |
| Margolis Index = 1 | 0·87 [0·62, 1·21] | 0·79 [0·57, 1·10] | 0·91 [0·65, 1·27] |
| Margolis Index = 2 | 1·32 [0·63, 2·79] | 0·68 [0·30, 1·52] | 0·51 [0·25, 1·05] |

*Legend: EBC – evidence-based compression; 2LB – two-layer bandage; CW – compression wraps; ITT – intention to treat*

**Fig D: Time to blind assessed or nurse reported ulcer healing – Kaplan-Meier healing estimates by allocation^a^**


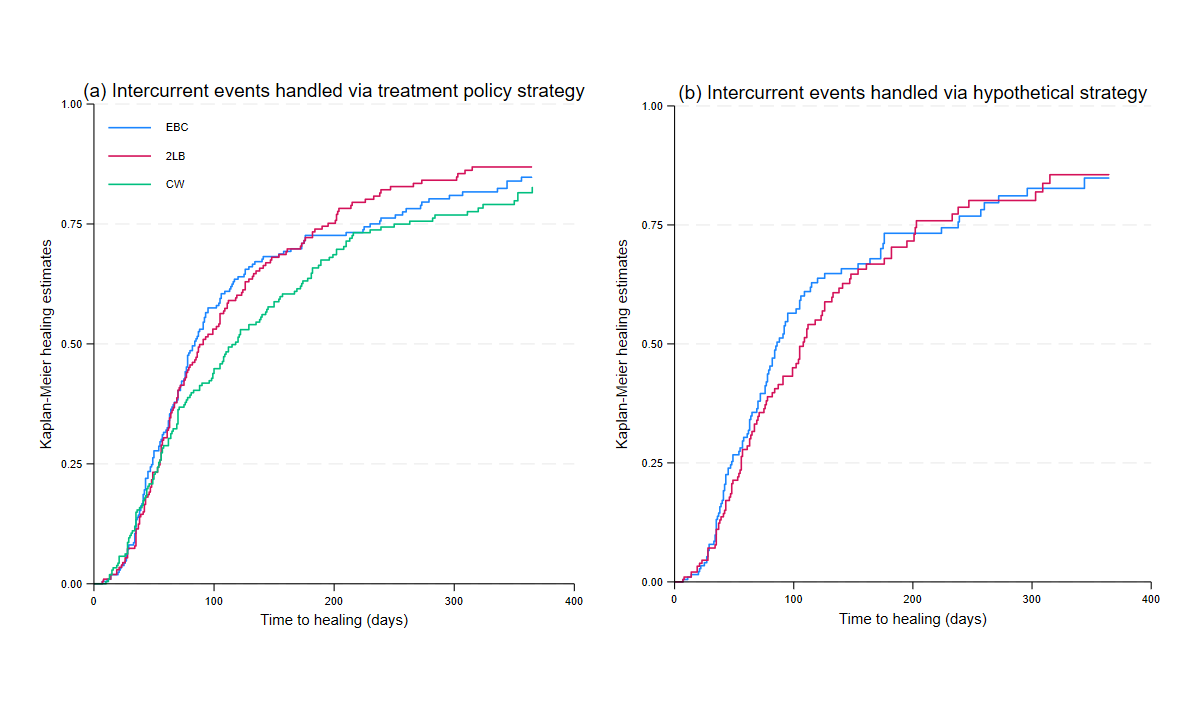


^a^Note that the estimand handling intercurrent events under a hypothetical strategy is only estimated for the non-inferiority comparison of EBC and 2LB

*Legend: EBC – evidence-based compression; 2LB – two-layer bandage; CW – compression wraps;*

**Table F: Time to blind assessed or nurse reported ulcer healing – tests of proportional hazards assumption for treatment effects**

| **Effect** | **p-value for test of proportional hazards** | | |
| --- | --- | --- | --- |
|  | Time | Log(time) | Rank |
| 2LB vs EBC (intercurrent events handled via treatment policy strategy) | 0·318 | 0·266 | 0·203 |
| 2LB vs EBC (intercurrent events handled via hypothetical strategy) | 0·350 | 0·478 | 0·347 |
| CW vs EBC (intercurrent events handled via treatment policy strategy) | 0·298 | 0·653 | 0·555 |
| CW vs 2LB (intercurrent events handled via treatment policy strategy) | 0·962 | 0·515 | 0·504 |

*Legend: EBC – evidence-based compression; 2LB – two-layer bandage; CW – compression wraps*

**Table G: Time to blind assessed or nurse reported ulcer healing – estimated hazard ratios**

| **Comparison** | **Estimand** | **Estimate [95% CI^a^]** | **p-value^b^** |
| --- | --- | --- | --- |
| Non-inferiority | Cause-specific hazard ratio comparing EBC vs 2LB (intercurrent events handled under a treatment policy strategy via a modified intention-to-treat analysis) | 0·94 [0·75,1·17] | - |
|  | Cause-specific hazard ratio comparing EBC vs 2LB (intercurrent events handled under a hypothetical strategy by censoring healing times at the first occurrence of a relevant intercurrent event and applying inverse probability of censoring weights) | 1·08 [0·82,1·42] | - |
| Superiority | Cause-specific hazard ratio comparing CW vs EBC (intercurrent events handled under a treatment policy strategy via a modified intention-to-treat analysis) | 0·86 [0·69,1·08] | 0·205 |
|  | Cause-specific hazard ratio comparing CW vs 2LB (intercurrent events handled under a treatment policy strategy via a modified intention-to-treat analysis) | 0·81 [0·65,1·02] | 0·068 |

^a^95% confidence intervals obtained via non-parametric bootstrapping (bias-corrected, 1000 replicates) for the analysis with inverse probability of censoring weights. For all other comparisons, Wald method 95% confidence intervals based on the estimated variance-covariance matrix of the fitted model are reported.

^b^For two-sided test of HR = 1

*Legend: EBC – evidence-based compression; 2LB – two-layer bandage; CW – compression wraps; ITT – intention to treat*

**Fig E: Time to blind assessed or nurse reported ulcer healing (modified ITT analysis set) – sample averaged healing functions by allocation and differences in median healing times**


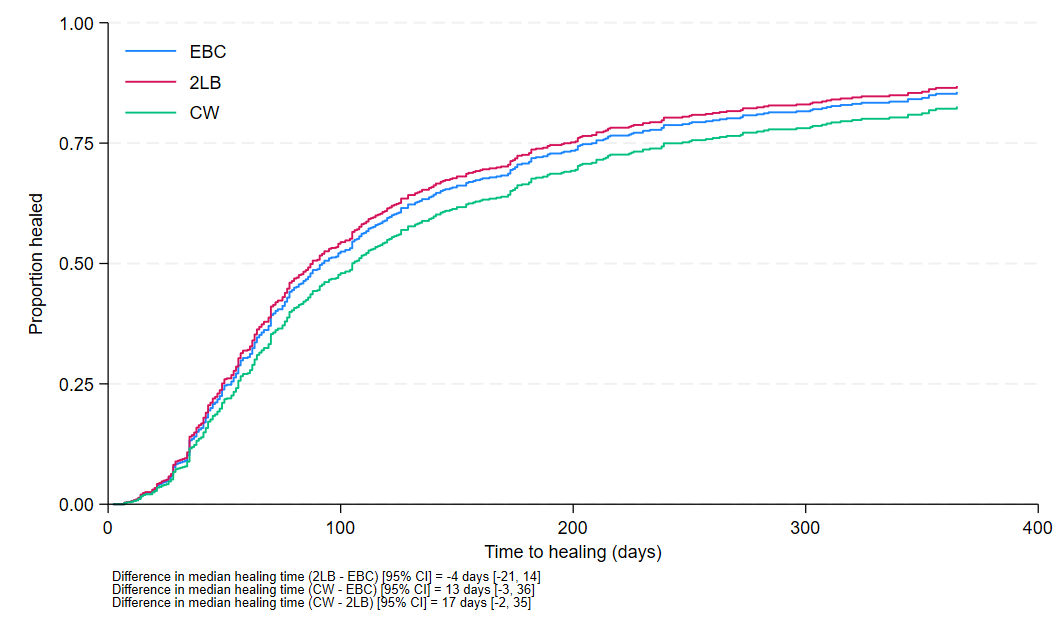


*Legend: EBC – evidence-based compression; 2LB – two-layer bandage; CW – compression wraps; ITT – intention to treat*

**Table H: Time to blind assessed or nurse reported ulcer healing (modified ITT analysis set) – sample averaged differences in cumulative incidence of healing at 1, 3, 6 and 12 months**

| **Time horizon** | **Contrast** | **Estimate [95% CI]** |
| --- | --- | --- |
| 1 month | Pr(Healed \| 2LB) - Pr(Healed \| EBC) | 0·52% [-1·35,2·54] |
|  | Pr(Healed \| CW) - Pr(Healed \| EBC) | -1·10% [-3·07,0·77] |
|  | Pr(Healed \| CW) - Pr(Healed \| 2LB) | -1·62% [-3·73,0·34] |
| 3 months | Pr(Healed \| 2LB) - Pr(Healed \| EBC) | 1·9 6% [-4·55,9·01] |
|  | Pr(Healed \| CW) - Pr(Healed \| EBC) | -4·4 0% [-11·62,2·96] |
|  | Pr(Healed \| CW) - Pr(Healed \| 2LB) | -6·3 6% [-13·70,1·35] |
| 6 months | Pr(Healed \| 2LB) - Pr(Healed \| EBC) | 1·7 8% [-3·93,8·02] |
|  | Pr(Healed \| CW) - Pr(Healed \| EBC) | -4·2 5% [-11·31,1·83] |
|  | Pr(Healed \| CW) - Pr(Healed \| 2LB) | -6·0 3% [-12·70,1·18] |
| 12 months | Pr(Healed \| 2LB) - Pr(Healed \| EBC) | 1·2 1% [-2·42,5·20] |
|  | Pr(Healed \| CW) - Pr(Healed \| EBC) | -3·0 4% [-7·97,0·96] |
|  | Pr(Healed \| CW) - Pr(Healed \| 2LB) | -4·2 4% [-9·12,0·20] |

*Legend: EBC – evidence-based compression; 2LB – two-layer bandage; CW – compression wraps; ITT – intention to treat*

**Fig F: Time to nurse reported ulcer healing – Kaplan-Meier healing estimates by allocation^a^**


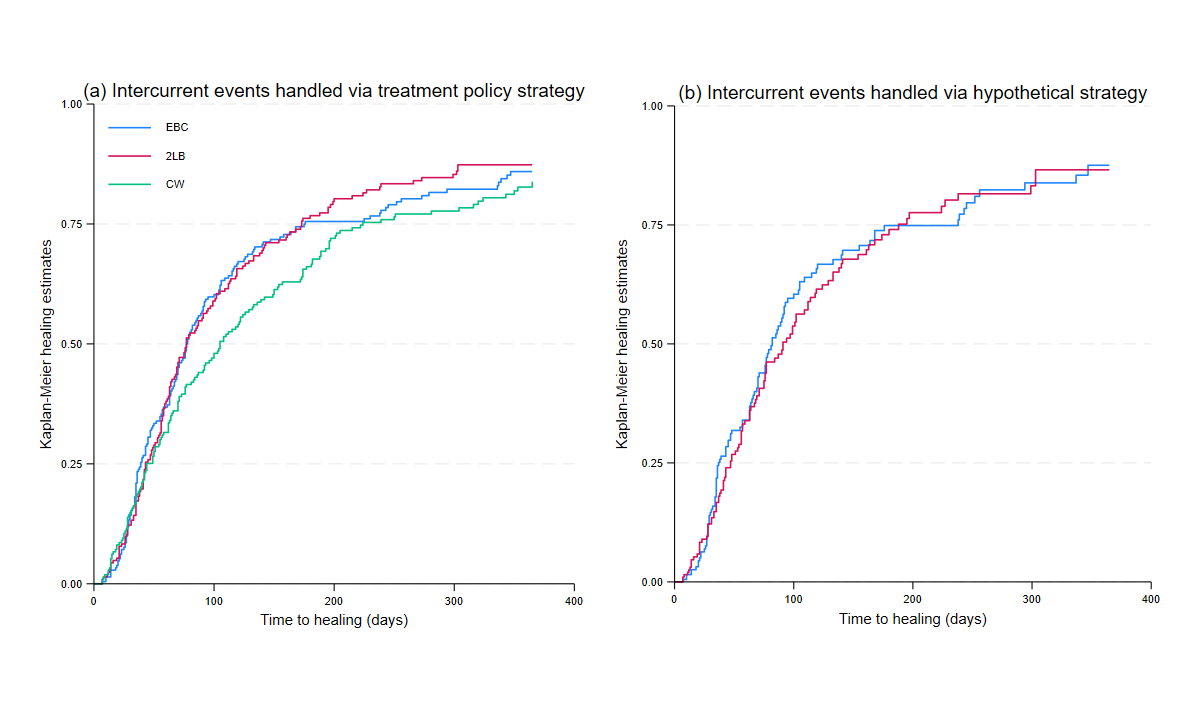


^a^Note that the estimand handling intercurrent events under a hypothetical strategy is only estimated for the non-inferiority comparison of EBC and 2LB

*Legend: EBC – evidence-based compression; 2LB – two-layer bandage; CW – compression wraps*

**Table I: Time to nurse reported ulcer healing – tests of proportional hazards assumption for treatment effects**

| **Effect** | **p-value for test of proportional hazards** | | |
| --- | --- | --- | --- |
|  | Time | Log(time) | Rank |
| 2LB vs EBC (intercurrent events handled via treatment policy strategy) | 0·535 | 0·496 | 0·362 |
| 2LB vs EBC (intercurrent events handled via hypothetical strategy) | 0·750 | 0·889 | 0·638 |
| CW vs EBC (intercurrent events handled via treatment policy strategy) | 0·280 | 0·803 | 0·553 |
| CW vs 2LB (intercurrent events handled via treatment policy strategy) | 0·643 | 0·672 | 0·758 |

*Legend: EBC – evidence-based compression; 2LB – two-layer bandage; CW – compression wraps*

**Table J: Time to nurse reported ulcer healing – estimated hazard ratios**

| **Comparison** | **Estimand** | **Estimate [95% CI^a^]** | **p-value^b^** |
| --- | --- | --- | --- |
| Non-inferiority | Cause-specific hazard ratio comparing EBC vs 2LB (intercurrent events handled under a treatment policy strategy via a modified intention-to-treat analysis) | 0·94 [0·76,1·17] | - |
|  | Cause-specific hazard ratio comparing EBC vs 2LB (intercurrent events handled under a hypothetical strategy by censoring healing times at the first occurrence of a relevant intercurrent event and applying inverse probability of censoring weights) | 1·02 [0·77,1·41] | - |
| Superiority | Cause-specific hazard ratio comparing CW vs EBC (intercurrent events handled under a treatment policy strategy via a modified intention-to-treat analysis) | 0·85 [0·68,1·06] | 0·156 |
|  | Cause-specific hazard ratio comparing CW vs 2LB (intercurrent events handled under a treatment policy strategy via a modified intention-to-treat analysis) | 0·80 [0·64,1·00] | 0·048 |

^a^95% confidence intervals obtained via non-parametric bootstrapping (bias-corrected, 1000 replicates) for the analysis with inverse probability of censoring weights. For all other comparisons, Wald method 95% confidence intervals based on the estimated variance-covariance matrix of the fitted model are reported.

^b^For two-sided test of HR = 1

*Legend: EBC – evidence-based compression; 2LB – two-layer bandage; CW – compression wraps*

**Fig G: Time to nurse reported ulcer healing (modified ITT analysis set) – sample averaged healing functions by allocation and differences in median healing times**


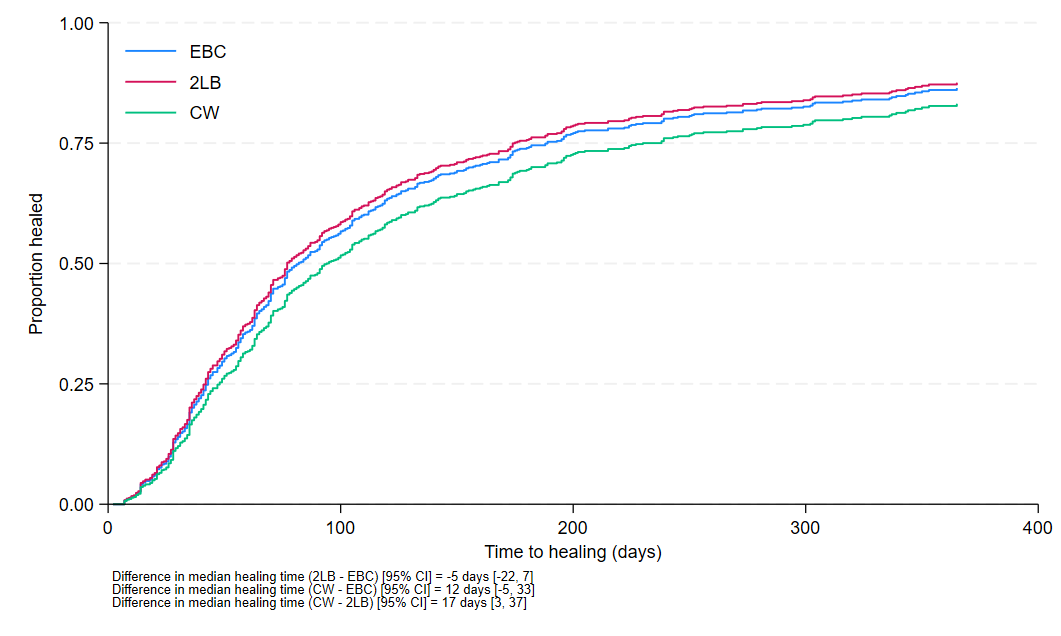


*Legend: EBC – evidence-based compression; 2LB – two-layer bandage; CW – compression wraps; ITT – intention to treat*

**Table K: Time to nurse reported ulcer healing (modified ITT analysis set) – sample averaged differences in cumulative incidence of healing at 1, 3, 6 and 12 months**

| **Time horizon** | **Contrast** | **Estimate [95% CI]** |
| --- | --- | --- |
| 1 month | Pr(Healed \| 2LB) - Pr(Healed \| EBC) | 0·79% [-2·15,3·69] |
|  | Pr(Healed \| CW) - Pr(Healed \| EBC) | -1·89% [-5·09,0·96] |
|  | Pr(Healed \| CW) - Pr(Healed \| 2LB) | -2·69% [-5·42,0·40] |
| 3 months | Pr(Healed \| 2LB) - Pr(Healed \| EBC) | 1·9 1% [-5·00,8·81] |
|  | Pr(Healed \| CW) - Pr(Healed \| EBC) | -4·9 4% [-13·00,2·20] |
|  | Pr(Healed \| CW) - Pr(Healed \| 2LB) | -6·8 5% [-13·15,0·71] |
| 6 months | Pr(Healed \| 2LB) - Pr(Healed \| EBC) | 1·6 5% [-4·17,7·47] |
|  | Pr(Healed \| CW) - Pr(Healed \| EBC) | -4·5 3% [-11·75,1·78] |
|  | Pr(Healed \| CW) - Pr(Healed \| 2LB) | -6·1 7% [-12·00,0·20] |
| 12 months | Pr(Healed \| 2LB) - Pr(Healed \| EBC) | 1·1 2% [-2·67,4·95] |
|  | Pr(Healed \| CW) - Pr(Healed \| EBC) | -3·2 5% [-8·46,0·71] |
|  | Pr(Healed \| CW) - Pr(Healed \| 2LB) | -4·3 8% [-8·71,-0·40] |

*Legend: EBC – evidence-based compression; 2LB – two-layer bandage; CW – compression wraps; ITT – intention to treat*

**Fig H: Time to reference leg healing (nurse reported) – Kaplan-Meier healing estimates by allocation**


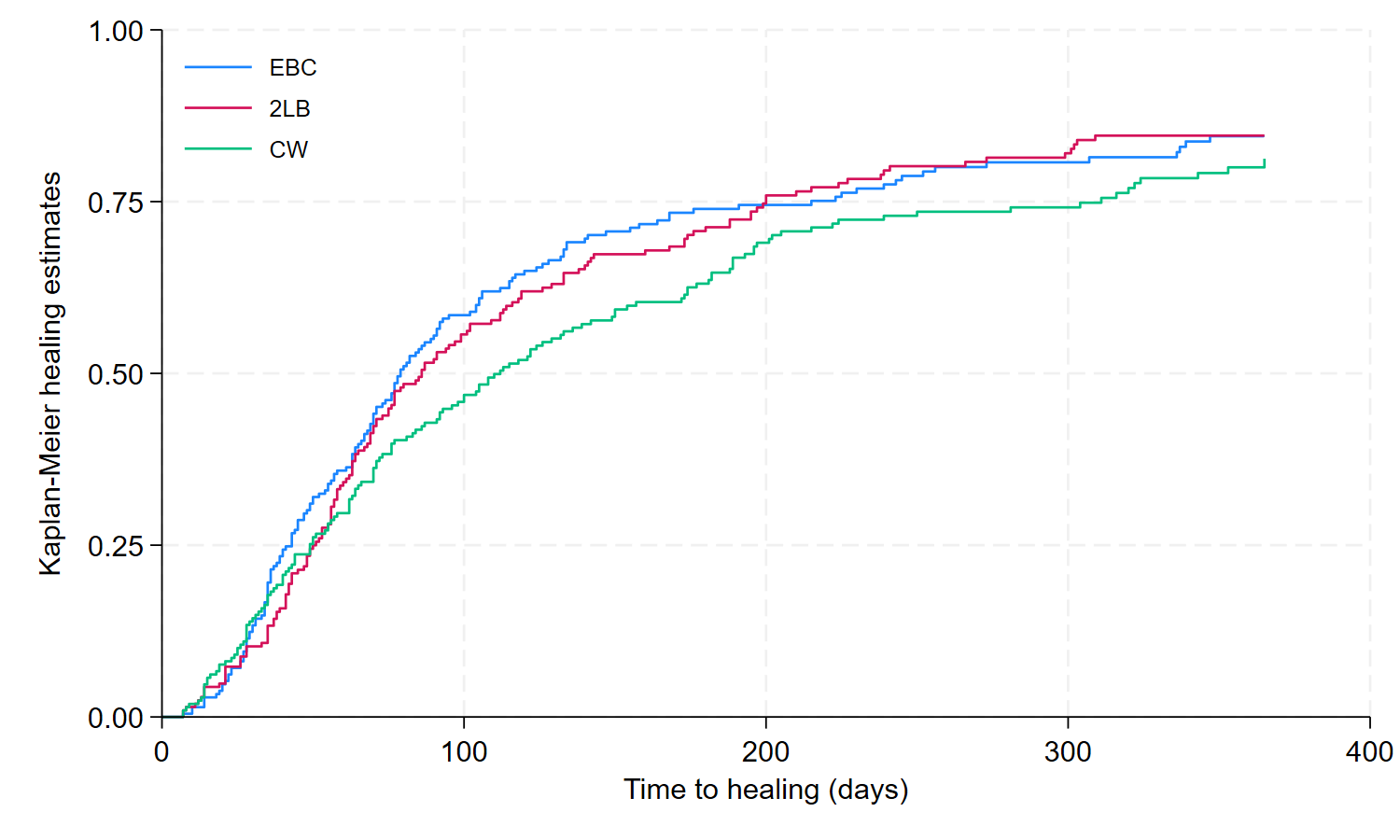


*Legend: EBC – evidence-based compression; 2LB – two-layer bandage; CW – compression wraps*

**Table L: Time to reference leg healing (nurse reported) – estimated hazard ratios**

| **Contrast** | **HR [95% CI]** | **p-value^a^** |
| --- | --- | --- |
| 2LB vs EBC | 1·01 [0·81,1·26] | 0·903 |
| CW vs EBC | 0·87 [0·70,1·09] | 0·232 |
| CW vs 2LB | 0·86 [0·69,1·08] | 0·187 |

^a^For two-sided test of HR = 1

*Legend: EBC – evidence-based compression; 2LB – two-layer bandage; CW – compression wraps*

**Fig I: Time to reference leg healing (nurse reported) - sample averaged healing functions by allocation and differences in median healing times**


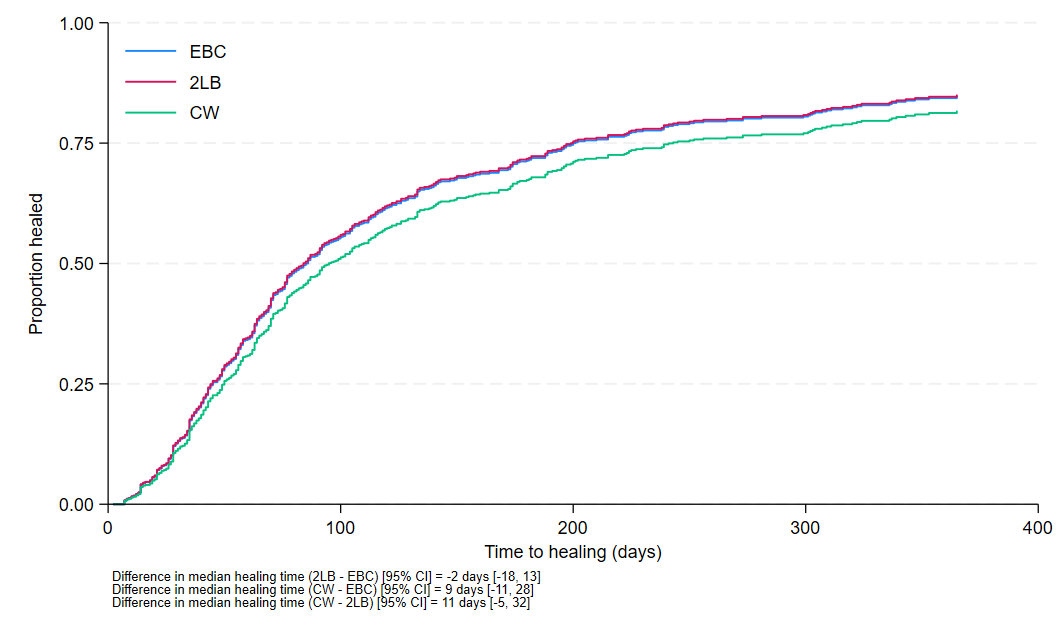


*Legend: EBC – evidence-based compression; 2LB – two-layer bandage; CW – compression wraps*

**Table M: Time to reference leg healing (nurse reported) - sample averaged differences in cumulative incidence of healing at 1, 3, 6 and 12 months**

| **Time horizon** | **Contrast** | **Estimate [95% CI]** |
| --- | --- | --- |
| 1 month | Pr(Healed \| 2LB) - Pr(Healed \| EBC) | 0·16% [-2·72,2·88] |
|  | Pr(Healed \| CW) - Pr(Healed \| EBC) | -1·54% [-4·24, 1·60] |
|  | Pr(Healed \| CW) - Pr(Healed \| 2LB) | -1·71% [-4·72 ,1·04] |
| 3 months | Pr(Healed \| 2LB) - Pr(Healed \| EBC) | 0·42% [-6·47, 6·89] |
|  | Pr(Healed \| CW) - Pr(Healed \| EBC) | -4·21% [-11·17, 3·99] |
|  | Pr(Healed \| CW) - Pr(Healed \| 2LB) | -4·63% [-12·10 ,2·65] |
| 6 months | Pr(Healed \| 2LB) - Pr(Healed \| EBC) | 0·39% [-5·71, 6·18] |
|  | Pr(Healed \| CW) - Pr(Healed \| EBC) | -4·01% [-10·39, 3·48] |
|  | Pr(Healed \| CW) - Pr(Healed \| 2LB) | -4·40% [-11·15, 2·20] |
| 12 months | Pr(Healed \| 2LB) - Pr(Healed \| EBC) | 0·28% [-3·97, 4·33] |
|  | Pr(Healed \| CW) - Pr(Healed \| EBC) | -3·04% [-7·70, 2·17] |
|  | Pr(Healed \| CW) - Pr(Healed \| 2LB) | -3·32% [-8·32, 1·15] |

*Legend: EBC – evidence-based compression; 2LB – two-layer bandage; CW – compression wraps*

**Fig J: Time to ulcer recurrence (nurse reported) among participants with complete healing of reference leg - Kaplan-Meier failure estimates by allocation**


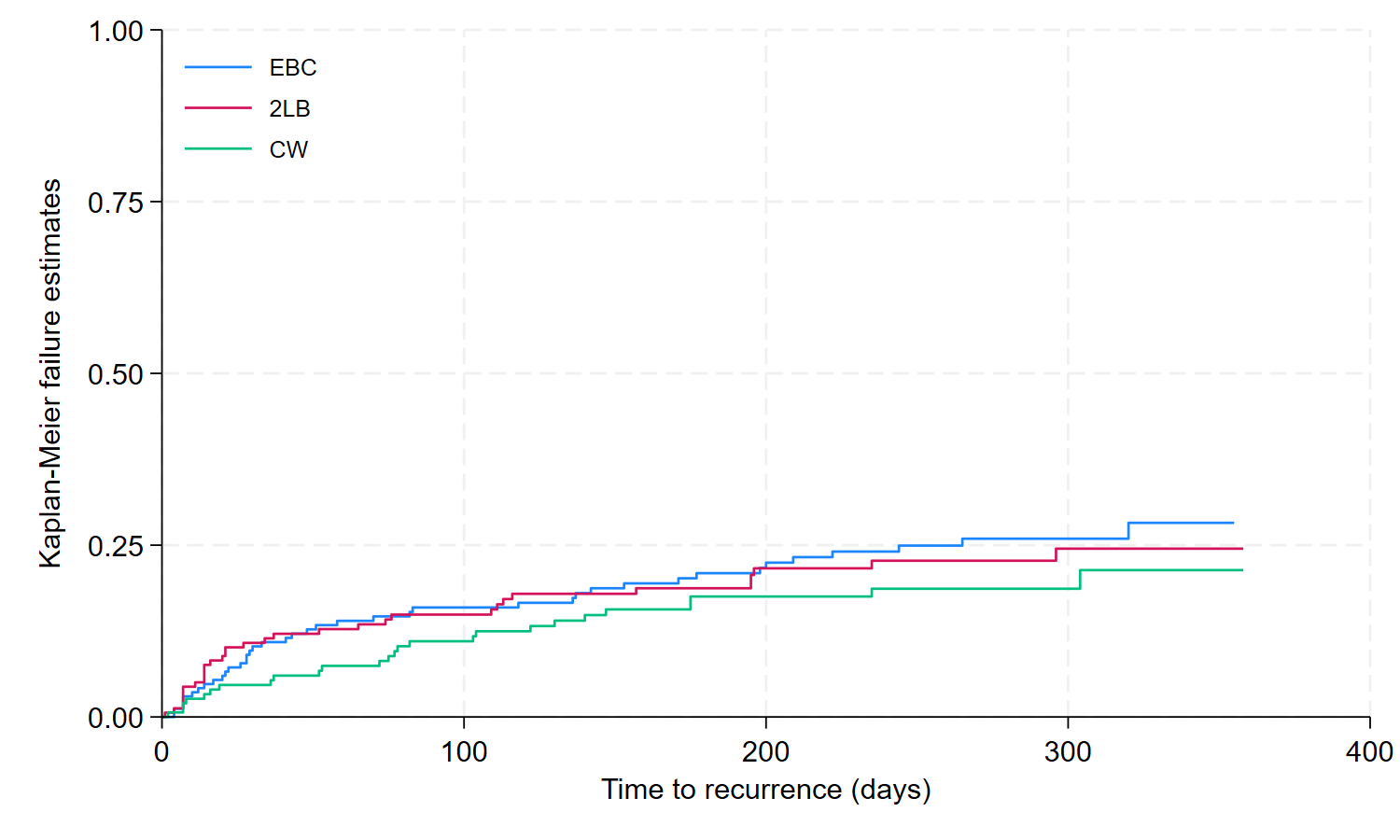


*Legend: EBC – evidence-based compression; 2LB – two-layer bandage; CW – compression wraps*

**Table N: Time to ulcer recurrence – Estimated hazard ratios**

| **Contrast** | **HR [95% CI)** | **p-value^a^** |
| --- | --- | --- |
| 2LB vs EBC | 0·89 [0·56, 1·42] | 0·619 |
| CW vs EBC | 0·81 [0·49, 1·34] | 0·414 |
| CW vs 2LB | 0·91 [0·54, 1·55] | 0·736 |

^a^For two-sided test of HR = 1

*Legend: EBC – evidence-based compression; 2LB – two-layer bandage; CW – compression wraps*

**Table O: Cumulative incidence of key clinical events by 6 and 12 months by allocation**

| **Event** | **Time Horizon** | **EBC**  **N (cumulative incidence**^a^**^)^** | **2LB**  **N (cumulative incidence ^a^)** | **CW**  **N (cumulative incidence ^a^)** |
| --- | --- | --- | --- | --- |
| Treatment to close remove incompetent, superficial veins | 6 months | 5 (2.49%)   - Endovenous ablation n=4 - Sclerotherapy n=1 | 4 (2.19%)   - Endovenous ablation n=2 - Avulsions n=1 - Clarivein n=1 | 10 (5.01%)   - Endovenous ablation n=5 - Sclerotherapy n=1 - Radiofrequency ablation n=1 - Endovenous ablation and Sclerotherapy n=1 - Endovenous ablation and Radiofrequency ablation n=2 |
|  | 12 months | 9 (4.95%)   - Endovenous ablation n=2 - Endovenous ablation and Sclerotherapy n=1 - Endovenous ablation and Avulsions n=1 | 5 (2.87%)   - Sclerotherapy n=1 | 15 (8.19%)   - Endovenous ablation n=5 |
| Hospital admission (related to venous leg ulcer) | 6 months | 3 (1.54%) | 3 (1.60%) | 4 (1.97%) |
|  | 12 months | 5 (2.82%) | 4 (2.69%) | 5 (2.57%) |
| All-cause mortality | 6 months | 2 (0.99%) | 3 (1.58%) | 5 (2.48%) |
|  | 12 months | 2 (0.99%) | 6 (3.41%) | 9 (4.81%) |

^a^For non-fatal events, cumulative incidence estimated using Nelson-Aalen cumulative hazard estimates (accounting for mortality). For all cause-mortality, cumulative incidence estimated using Kaplan-Meier survival estimates

*Legend: EBC – evidence-based compression; 2LB – two-layer bandage; CW – compression wraps*

**Table P: VEINES QoL - estimated differences in expected score**

|  | **Difference [95% CI^a^]** | **p-value^b^** |
| --- | --- | --- |
| **Month 3** |  |  |
| 2LB - EBC | 2·80 [-1·25,6·85] | 0·176 |
| CW - EBC | 0·87 [-3·19, 4·93] | 0·676 |
| CW - 2LB | -1·93 [-6·04, 2·17] | 0·355 |
| **Month 6** |  |  |
| 2LB - EBC | 2·96 [-1·56, 7·48] | 0·199 |
| CW - EBC | 0·24 [-4·28, 4·77] | 0·916 |
| CW - 2LB | -2·72 [-7·27, 1·84] | 0·242 |
| **Month 12** |  |  |
| 2LB - EBC | 2·05 [-3·55, 7·65] | 0·474 |
| CW - EBC | -0·43 [-5·98, 5·12] | 0·880 |
| CW - 2LB | -2·47 [-8·08, 3·13] | 0·387 |

^a^Based on t-distribution with degrees of freedom calculated using the method of Kenward and Roger

^b^For two-tailed test of difference = 0

*Legend: EBC – evidence-based compression; 2LB – two-layer bandage; CW – compression wraps*

**Table Q: VEINES Sym - estimated differences in expected score**

|  | **Difference [95% CI^a^)** | **p-value^b^** |
| --- | --- | --- |
| **Month 3** |  |  |
| 2LB - EBC | 0·50 [-3·95,4·96] | 0·825 |
| CW - EBC | -2·81 [-7·35, 1·73] | 0·225 |
| CW - 2LB | -3·31 [-7·86, 1·24] | 0·154 |
| **Month 6** |  |  |
| 2LB - EBC | 0·59 [-4·07, 5·25] | 0·804 |
| CW - EBC | 0·24 [-4·49, 4·97] | 0·919 |
| CW - 2LB | -0·35 [-5·11, 4·41] | 0·886 |
| **Month 12** |  |  |
| 2LB - EBC | 0·51 [-5·42, 6·45] | 0·865 |
| CW - EBC | -4·47 [-10·32, 1·37] | 0·134 |
| CW - 2LB | -4·99 [-10·93, 0·96] | 0·100 |

^a^Based on t-distribution with degrees of freedom calculated using the method of Kenward and Roger

^b^For two-tailed test of difference = 0

*Legend: EBC – evidence-based compression; 2LB – two-layer bandage; CW – compression wraps*

**Table R: Ulcer related pain score (0 - 100, higher scores = greater pain) - all participants (participants reporting no ulcers imputed with score of zero)**

|  | **EBC**  **(n = 213)** | **2LB**  **(n = 211)** | **CW**  **(n = 213)** | **Total**  **(n = 637)** |
| --- | --- | --- | --- | --- |
| **Ulcer Related Pain: Month 1** |  |  |  |  |
| N | 178 | 169 | 162 | 509 |
| Mean (SD) | 23·6 (27·3) | 20·4 (24·5) | 22·0 (25·6) | 22·0 (25·8) |
| Median (Q1, Q3) | 10·0 (0·0 ,45·0) | 10·0 (0·0, 40·0) | 10·0 (0·0, 45·0) | 10·0 (0·0, 40·0) |
| Min., Max. | 0·0 , 97·0 | 0·0 , 88·0 | 0·0 , 95·0 | 0·0 , 97·0 |
| **Ulcer Related Pain: Month 3** |  |  |  |  |
| N | 169 | 161 | 161 | 491 |
| Mean (SD) | 15·6 (24·1) | 14·5 (23·2) | 14·0 (22·9) | 14·7 (23·4) |
| Median (Q1, Q3) | 0·0 (0·0 , 25·0) | 1·0 (0·0 , 20·0) | 2·0 (0·0 , 20·0) | 1·0 (0·0 , 20·0) |
| Min., Max. | 0·0 , 100·0 | 0·0 , 100·0 | 0·0 , 85·0 | 0·0 , 100·0 |
| **Ulcer Related Pain: Month 6** |  |  |  |  |
| N | 155 | 144 | 148 | 447 |
| Mean (SD) | 11·3 (22·2) | 9·5 (21·3) | 11·7 (22·5) | 10·9 (22·0) |
| Median (Q1, Q3) | 0·0 (0·0 , 10·0) | 0·0 (0·0 , 5·0) | 0·0 (0·0 , 9·0 ) | 0·0 (0·0 , 7·0) |
| Min., Max. | 0·0 , 100·0 | 0·0 , 100·0 | 0·0 , 90·0 | 0·0 , 100·0 |
| **Ulcer Related Pain: Month 12** |  |  |  |  |
| N | 104 | 96 | 99 | 299 |
| Mean (SD) | 13·4 (25·8) | 9·4 (21·2) | 7·0 (15·9) | 10·0 (21·5) |
| Median (Q1, Q3) | 0·0 (0·0 , 7·5) | 0·0 (0·0 , 4·0) | 0·0 (0·0 , 5·0) | 0·0 (0·0 , 5·0) |
| Min., Max. | 0·0 , 100·0 | 0·0 , 100·0 | 0·0 , 75·0 | 0·0 , 100·0 |

*Legend: EBC – evidence-based compression; 2LB – two-layer bandage; CW – compression wraps*
