# Supplementary material for: Compression therapies for venous leg ulcers: The VENous Ulcer Study 6 (VenUS 6), an open, multicentre, randomised clinical trial
Source: PLoS Med. 2026 Jul 10;23(7):e1005154. doi: 10.1371/journal.pmed.1005154 (PMC13354008; doi:10.1371/journal.pmed.1005154)
Supplement: S2 File — (DOCX) [file pmed.1005154.s002.docx]

| Item | | Descriptor | Page or Line |
| --- | --- | --- | --- |
| Title | | Identification of the study as randomised | Page 1 |
| Authors^[*](https://www.sciencedirect.com/science/article/pii/S0140673607618352?via%3Dihub#tbl1fn1)^ | | Contact details for the corresponding author | Page 1 |
| Trial design | | Description of the trial design (eg, parallel, cluster, non-inferiority) | Page 2; Line 42 |
| Methods | |  |  |
|  | Participants | Eligibility criteria for participants and the settings where the data were collected | Page 2; Lines 45-46 |
|  | Interventions | Interventions intended for each group | Page 2; Lines 46-48 |
|  | Objective | Specific objective or hypothesis | Page 2; Lines 39-41 |
|  | Outcome | Clearly defined primary outcome for this report | Page 2; Lines 48-49 |
|  | Randomisation | How participants were allocated to interventions | Page 2; Line 46 |
|  | Blinding (masking) | Whether or not participants, care givers, and those assessing the outcomes were blinded to group assignment | Page 2; Line 44 |
| Results | |  |  |
|  | Numbers randomised | Number of participants randomised to each group | Page 2; Line 51 - 52 |
|  | Recruitment | Trial status | Page 2; Lines 42-44 |
|  | Numbers analysed | Number of participants analysed in each group | Page 2; Line 51 - 52 |
|  | Outcome | For the primary outcome, a result for each group and the estimated effect size and its precision | Page 2; Lines 55-57 |
|  | Harms | Important adverse events or side-effects | Page 2; Line 58-60 |
| Conclusions | | General interpretation of the results | Page 2; Lines 61-64 |
| Trial registration | | Registration number and name of trial register | Page 2; Line 65 |
| Funding | | Source of funding | Page 2; Line 66-67 |

CONSORT Checklist for Abstracts
