# Supplementary material for: Compression therapies for venous leg ulcers: The VENous Ulcer Study 6 (VenUS 6), an open, multicentre, randomised clinical trial
Source: PLoS Med. 2026 Jul 10;23(7):e1005154. doi: 10.1371/journal.pmed.1005154 (PMC13354008; doi:10.1371/journal.pmed.1005154)
Supplement: S4 File — (DOCX) [file pmed.1005154.s004.docx]

**CONSERVE-CONSORT Extension**

| **Item** | **Item Title** | **Description** | **Page No.** | |
| --- | --- | --- | --- | --- |
| I. | Extenuating Circumstances | Describe the circumstances and how they constitute  extenuating circumstances. | Discussion Section – Paragraph 4 and 10 | |
| II. | Important Modifications | Describe how the modifications are important  modifications. | Not applicable, no modifications made | |
|  |  | Describe the impacts and mitigating strategies,  including their rationale and implications for the  trial.  (see  below) | Discussion Section – Paragraph 4 and 10 | |
|  |  | Provide a modification timeline | Not applicable, no modifications made | |
| III | Responsible Parties | State who planned, reviewed and approved the  modifications. | Not applicable, no modifications made | |
| IV | Interim data | If modifications were informed by trial data, describe  how the interim data were used, including whether  they were examined by study group, and whether the  individuals reviewing the data were blinded to the  treatment allocation. | Not applicable, no modifications made | |
| **CONSORT Number and Item** | For each row, if important modifications occurred  check “direct impact” and/or “mitigating strategy” and  describe the changes in the trial manuscript or  supplement. Check “no change” for items that are  unaffected in the extenuating circumstance. | |  | |
|  | **No Change** | **Impact*** | **Mitigating**  **Strategy**** | **Page No.** |
| **1 Title and abstract** |  |  |  |  |
| 2 Introduction | **x** |  |  |  |
| 3 Methods: Trial Design | **x** |  |  |  |
| 4 Methods: Participants | **x** |  |  |  |
| 5 Methods: Interventions | **x** |  |  |  |
| 6 Methods: Outcomes | **x** |  |  |  |
| 7 Methods: Sample Size | **x** |  |  |  |
| 8-10 Methods: Randomisation | **x** |  |  |  |
| 11 Methods: Blinding | **x** |  |  |  |
| 12 Methods: Statistical methods | **x** |  |  |  |
| 13 Results: Participant flow | **x** |  |  |  |
| 14 Results: Recruitment | **x** |  |  |  |
| 15 Results: Baseline data | **x** |  |  |  |
| 16 Results: Numbers analysed | **x** |  |  |  |
| 17 Results: Outcomes and  estimation | **x** |  |  |  |
| 18 Results: Ancillary analyses | **x** |  |  |  |
| 19 Results: Harms | **x** |  |  |  |
| 20 Discussion: Limitations | **x** |  |  |  |
| 21 Discussion: Generalisability | **x** |  |  |  |
| 22 Other information Registration | **x** |  |  |  |
| 23 Other information: Protocol | **x** |  |  |  |
| 24 Other information: Funding | **x** |  |  |  |

*Aspects of the trial that are directly affected or changed by the extenuating circumstance and are not

under the control of investigators, sponsor or funder.

**Aspects of the trial that are modified by the study investigators, sponsor or funder to respond to the

extenuating circumstance or manage the direct impacts on the trial.

The CONSERVE-CONSORT Checklist is licensed by the CONSERVE Group under the Creative Commons Attribution-NonCommercial-NoDerivs 4.0 International license.
