# Supplementary material for: Compression therapies for venous leg ulcers: The VENous Ulcer Study 6 (VenUS 6), an open, multicentre, randomised clinical trial
Source: PLoS Med. 2026 Jul 10;23(7):e1005154. doi: 10.1371/journal.pmed.1005154 (PMC13354008; doi:10.1371/journal.pmed.1005154)
Supplement: S5 File — (PDF) [file pmed.1005154.s005.pdf]

THE UNIVERSITY OF MANCHESTER

VenUS 6: A randomised controlled trial of compression therapies for the treatment of venous leg ulcers.

Study protocol

Jo Dumville

Sponsor Reference: **B00947**
REC Reference: 20/WS/0120
IRAS Reference: **280987**
Date and Version number: **Version 1.6 17.07.2023**

Chief Investigator: Professor Jo Dumville
University of Manchester
jo.dumville@manchester.ac.uk

Sponsor: Manchester University NHS Foundation Trust

Funder: NIHR Health Technology Assessment Programme (NIHR128625)

Trials Unit: York Trials Unit, Department of Health Sciences, University of York, York YO10 5DD

Funding Acknowledgement: This project is funded by the NIHR Health Technology Assessment Programme (NIHR128625).

Department of Health and Social Care disclaimer: The views expressed are those of the authors and not necessarily those of the NHS, the NIHR or the Department of Health and Social Care

SIGNATURE PAGE

The undersigned confirm that the following protocol has been agreed and accepted and that the Chief Investigator agrees to conduct the study in compliance with the approved protocol and will adhere to the principles outlined in the Declaration of Helsinki, the Sponsor's SOPs, and other regulatory requirement.
I agree to ensure that the confidential information contained in this document will not be used for any other purpose other than the evaluation or conduct of the investigation without the prior written consent of the Sponsor
I also confirm that I will make the findings of the study publically available through publication or other dissemination tools without any unnecessary delay and that an honest accurate and transparent account of the study will be given; and that any discrepancies from the study as planned in this protocol will be explained.

For and on behalf of the Study Sponsor:
Signature: L Webster .... Date: 16/11/2023
Name (please print): LYNNE WEBSTER ....
Position: Director of Research Governance & Quality ....

Chief Investigator:
Signature: Jo Dumville .... Date: ..13/..11/...2023
Name: (please print): Jo Dumville ....

This project will be conducted in accordance with the study protocol and the ethical principles outlined by Good Clinical Practice (GCP) and the Declaration of Helsinki in its most current version.

Contents

SIGNATURE PAGE 1

1. Background and Study Rationale 5

1.1 Venous leg ulcers 5

1.2. Current evidence on compression treatments for venous leg ulcers 5

Compression wraps 5

Four-layer bandage 6

Two-layer compression hosiery 6

Two-layer bandage 6

Combining all RCT evidence on full compression treatments 6

2. Study objectives 6

3. Study design 7

3.1 Overview 7

3.2 Setting 8

3.3 Studies Within A Trial 9

Recruitment SWAT 9

Retention SWAT 9

4. Study Population 11

4.1 Inclusion criteria 11

4.2 Exclusion criteria 12

5. Recruitment, randomisation and withdrawal processes 12

5.1 Identification of potential participants and recruitment 12

5.2 Definition of reference ulcer and reference leg 13

5.3 Randomisation 13

5.4 Participant withdrawal and trial exit 14

6. Trial Treatments (Appendix 1 contains further details of treatment specifications) 14

6.1 Compression wraps 14

6.2 Two-layer compression bandage 15

6.3 Evidence-based compression (compression hosiery or the four-layer bandage) 15

6.4 Primary contact layer 15

7. Data collection (excluding adverse event data, see section 8) 16

7.1 Staff-collected data 16

Healing of the reference ulcer 16

Treatments-received (including changes to trial treatment) 16

Healing of the reference leg *17*
Recurrence *17*
7.2 Participant self-reported data 17
Health related quality of life and utility data (participant self-report at baseline, 3, 6, and 12 months) *18*
Resource use participant self-report at 3, 6, and 12 months. *18*
Wound-related pain participant self-report at baseline, 1, 3, 6, and 12 months. *18*
Treatment adherence and ease of use (month 1, 3, 6 and 12 months) *18*
7.3 Additional data collection 19
Participant death (following trial exit) *19*
7.4 Source data definition, transfer and storage 20
7.5 Level of blinding 21
7.6 Final Study Data Set 21
8. Adverse Events 21
8.1 Definition of adverse events 21
8.2 Serious Adverse Events 22
8.3 Reporting Procedures for Adverse and Serious Adverse Events 22
9. Statistical and Economic Considerations 23
9.1. Sample size for recruitment 23
9.2 Internal pilot 23
9.3 Full-trial analyses 24
9.4 Evidence synthesis for primary outcome 25
9.5 Cost-effectiveness analyses 26
9.6 SWAT analyses 27
Recruitment SWAT *27*
Retention SWAT *27*
10. Ethical Arrangements 28
10.1 Ethical Approval 28
10.2 Risks and Benefits 29
10.3 Informing Participants of Potential Risks and Benefits 29
10.4 End of Trial 29
10.5 Retention of Trial Documentation 29
11. Trial Finance and Insurance 30
11.1 Trial Funding 30
11.2 Trial Insurance 30
12. Project Management 30
12.1 Trial Sponsor 30

12.2 Trial Management 30
12.3 Trial Management Group 31
12.4 Trial Steering and Data Monitoring Committees 31
12.5 Patient and Public Involvement (PPI) 31
Patient Advisory Group 31
13. Dissemination and projected outputs 31
VENUS 6 Trial Project Management Plan ..... **Error! Bookmark not defined.**
REFERENCE LIST 25
Appendix 1 ..... **Error! Bookmark not defined.**

1. Background and Study Rationale

1.1 Venous leg ulcers

Venous leg ulcers are common, recurring open wounds on the lower leg. In the UK, venous leg ulcer care is mainly delivered in the community, often in patients' homes or clinics, by nurses or other health professionals. Previous influential randomised controlled trial (RCT) evidence shows that high (or full) compression treatments (aiming to deliver around 40mmHg at the ankle) reduce ulcer healing time.[1] Full compression is the first line treatment for venous leg ulcers although the EVRA trial has recently shown that early endovenous ablation surgery results in faster healing and is cost-effective. [2,3] Whilst early use of endovenous ablation is likely to increase over time, most people with a venous leg ulcer will continue to spend many weeks being treated in the community with compression whilst waiting for referral and then surgery. Furthermore, some people with ulcers cannot or will not have surgery. Maximising ulcer-free days and cost-effectiveness with optimal compression use therefore remains important to patients and the NHS.

The main full compression treatments relevant here are:

- choice of four-layer bandage or two-layer compression hosiery (**evidence-based compression**);
- the two-layer bandage;
- adjustable hook-and-loop fastened compression systems (termed “compression wraps” here).

Two-layer hosiery and compression wraps can be given to people for self-application in some cases whereas trained, skilled staff must apply bandages.

Data from NIHR Health Technology Assessment (HTA) funded RCTs suggest that the four-layer bandage and two-layer hosiery confer similar healing times but two-layer hosiery is cost effective [4,5]. Current best practice compression is two-layer hosiery for eligible, willing and able people (i.e. those with non-oedematous legs and often more mobile and dexterous patients) and four-layer bandages for those unsuited to hosiery. We call this ‘choice’ approach ‘**evidence-based compression’ (EBC).** There has been much less research on two-layer bandages: there are few data from trials and existing evidence is highly uncertain. There is almost no evidence available on the clinical- and cost-effectiveness of compression wraps.

Despite the lack of supportive research evidence, national prescribing data suggest that the two-layer bandage is the most commonly-used compression treatment and that use of the four-layer bandage has declined accordingly. Two-layer hosiery use has increased (conferring estimated annual cost savings of over £2 million in 2017 calculated by applicants using prescribing data) but overall usage of hosiery is much lower than bandage use. Prescribing data suggest that compression wrap use is increasing but remains much lower than bandage or hosiery use.

1.2. Current evidence on compression treatments for venous leg ulcers

<u>*Compression wraps*</u>

There is only one published RCT evaluating compression wraps [6] involving only 24 participants with bilateral ulcers: one leg being randomised to a form of compression wrap and the other leg to the four-layer bandage. The duration of follow-up was 12 weeks and the primary outcome was change in ulcer size rather than full healing. The authors concluded there was a significant

benefit associated with compression wraps in terms of reduced ulcer area. However, the trial was of very low quality because of risk of bias, imprecision and incorrect data analysis. The results of this study as presented cannot be meaningfully interpreted.

Four-layer bandage

VenUS I (NIHR-HTA funded) compared the four-layer and short-stretch bandages in 387 randomised participants.[7,8] The four-layer bandage significantly reduced time to healing compared with the short stretch bandage: adjusted hazard ratio 1.33; 95% CI 1.05 to 1.67. This study finding was strengthened in a subsequent systematic review and meta-analysis of individual patient data of these two treatments (n = 797) [9] and we regard this as high certainty evidence using GRADE. Whilst the four-layer bandage is considered a gold standard full compression treatment for people with venous leg ulcers in the UK, its use is decreasing in favour of other treatment options associated with much greater uncertainty of effect.

Two-layer compression hosiery

VenUS IV (NIHR-HTA funded), compared the clinical- and cost-effectiveness of the four-layer bandage and two-layer compression hosiery kits in 457 participants over a maximum of 12 months follow-up [4,5]. There was no difference in the time to healing of venous leg ulcers: (adjusted hazard ratio 0.99 95% CI 0.79 to 1.25) but the hosiery was cost effective – reducing mean per patient annual costs by £302 (bias corrected 95% CI −£716.3 to £96.5): this has a GRADE assessment of moderate certainty evidence (downgraded for imprecision).

Two-layer bandage

The two-layer bandage is a widely used alternative to the four-layer bandage. It aims to deliver the same amount of compression using fewer layers of bandage so the treatment is, in theory, less bulky and easier to apply. However we do not believe this treatment has been fully evaluated: three trials (total n = 299) [10-12] have compared two-layer and four-layer bandages and reported proportions of wounds healed over durations of follow up ranging from 4 to 12 weeks. We pooled these data (for healing at any time point) and found great uncertainty about the relative effects of the two-layer and four-layer bandages, consistent with potentially real and clinically important treatment effects in either direction or no difference (risk ratio = 1.17; 95% CI 0.87 to 1.57). We determine this to be low certainty evidence using GRADE.

Combining all RCT evidence on full compression treatments

We have updated our previous network meta-analysis to combine all RCT data comparing four-layer bandages, two-layer bandages, two-layer hosiery and the short-stretch bandage [3]. We could not include wraps as there are no suitable data available. The updated analysis determines two-layer bandages likely to be the most effective full compression treatment but this estimate is highly uncertain: hazard ratio for healing with two-layer bandage compared with the four layer bandage = 1.21 (95% CI 0.82 to 1.74).

2. Study objectives

VenUS 6 is a parallel group, randomised controlled trial with internal pilot and process evaluation. The study will address the following primary research objectives:

- to obtain robust estimates of recruitment rates and confirm trial feasibility in a 6 month, internal pilot phase;
- to compare compression wraps with EBC in terms of the time to healing of venous leg ulcers;

- to determine whether two-layer bandages are non-inferior to EBC for time to healing of venous leg ulcers;
- to determine which is the cost-effective, full compression treatment for venous leg ulcers;

The trial will also compare the effects of compression wraps with EBC and two-layer bandages in terms of:

- ulcer recurrence
- reported adverse events
- wound-related pain
- health related quality of life
- adherence to treatment

3. Study design

3.1 Overview

VenUS 6 is a multi-centred, pragmatic, parallel group, randomised, controlled, three arm trial. We will randomise people with one or more venous leg ulcers to be offered:

Arm 1: Compression wraps (Adjustable hook-and-loop-fastened compression)
Arm 2: EBC treatment (choice of four-layer bandage or two-layer compression hosiery)
Arm 3: Two-layer bandage.

The study has a 45-month recruitment period in total (comprising an initial 32 month planned recruitment period plus a 13 month extension), including an initial 6 month internal pilot. Follow-up will be variable with participants followed for minimum of 4 months and a maximum of 12 months. A flow diagram demonstrating the patient/participant pathway through the study is provided in Figure 1.

We cannot blind participants nor health care practitioners to treatment allocation thus the primary outcome (time to healing) will be verified by independent, blinded observers using standardised photographs (see section 7.1). The independent, blinded observers will be experienced tissue viability nurses who have not been involved in any patient care in relation to the VenUS 6 Trial. They will undergo full training in line with other trials that have used this approach. Photographs will be anonymised to ensure participant confidentiality.

Secondary trial outcomes are ulcer recurrence, adverse events, health related quality of life and health utility, resource use, wound-related pain, treatment adherence and ease of use.

This is a pragmatic trial: following randomisation, as well as being offered their allocated compression treatment, participants will receive standard care, including wound dressing changes, as per routine clinical practice. Data on treatment use and clinical outcomes will be collected during this period by nurses. During the trial, participants will also be asked to complete outcome assessments (sent in the post) at 1, 3, 6, and 12 months post-randomisation (see section 7.2).

Semi-structured interviews will explore the views and experiences of participants and nurses on the different compression therapies. Interviews will be conducted between 1 and 9 months follow-up, subject to receiving consent.

3.2 Setting

The study will enlist up to 35 sites (NHS Trusts and primary care centres) with the aim of recruiting two participants per site per month, over the two and a half year recruitment period. It is anticipated that 25 sites will be recruiting at any one time.

Figure 1: Summary of patient/participant pathway

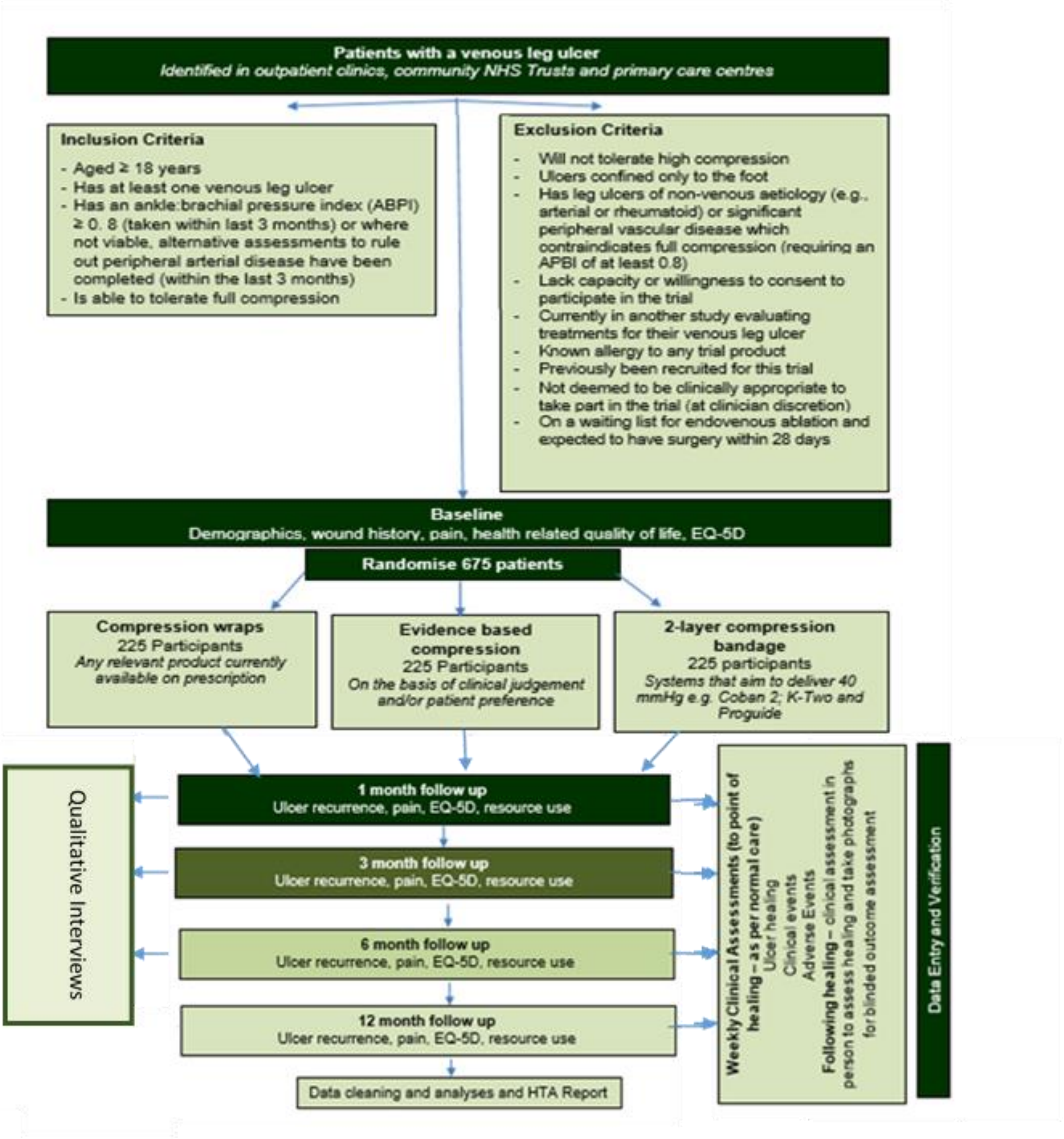

3.3 Studies Within A Trial

We will undertake four studies within a trial (SWATs) to assess the effectiveness for methods to improve recruitment and retention. Strategies have been registered, or will be registered with the Medical Research Council SWAT repository prior to activity commencing.

Recruitment SWAT
We will evaluate the effects of presentation of the study design to participants on recruitment rate. Participants will be randomised (at the site level) to receive an infographic (visual document explaining the study) plus the standard patient information sheet (PIS) or just the PIS.

A recent review showed that tailoring or shortening the patient information sheet given to participants makes little or no difference to recruitment. [13] None of the studies included in the review, however, tested the use of information graphics ("infographics") to enhance recruitment. The evidence around the effectiveness of infographics in a health context is limited but persuasive. Infographics have been shown to improve patient knowledge; both in relation to personally relevant information such as discharge instructions, and statistical information such as the association of age with cancer risk. [14,15] While a further study with patients, students and doctors found that infographics did not increase knowledge when compared to plain language summaries, the infographics did improve reader experience and user-friendliness.[16] These findings suggest that there may be the potential for infographics to increase potential participant experience and understanding of health research leading to increased recruitment.

This SWAT will be a cluster randomised trial; randomisation will be carried out at the site level to reduce cross contamination. The allocation ratio will be 1 to 1. Generation of the allocation sequence will be undertaken independently by a researcher not involved with the recruitment of participants. As is usual with embedded trials, the sample size is constrained by the number of patients approached about the study, hence a formal power calculation to determine sample size has not been conducted.

The primary outcome of this embedded trial will be the proportion of participants in each group who are randomised into the host trial. Secondary outcomes will include the proportion of patients in each group who are screened but do not go on to be randomised, and the cost-effectiveness of the intervention.

Retention SWAT 1
We will use a 2 by 2 factorial design to simultaneously evaluate the effect of two retention strategies: a participant newsletter and a thank you card sent in advance of follow up questionnaires. Participants will be randomised to receive 1) newsletter and thank you card; 2) newsletter only; 3) thank you card only; 4) neither the newsletter nor the thank you card. These will be sent at Month 4 and Month 9 following randomisation to the VenUS 6 trial.

Many recruitment and retention strategies routinely include some element of thanks within them. Although we often do this, there is little to no evidence to suggest this might work. Recent evidence suggests that those saying thank you often undervalue its effect. [17] Therefore, there is a need to test the impact of thanks in the context of trial recruitment and retention.

All participants recruited into the VenUS 6 trial, and who remain as fully participating (i.e. have not fully withdrawn, withdrawn from postal follow up or have died) will be eligible for the SWAT. There are no additional inclusion or exclusion criteria. Generation of the allocation sequence will be undertaken independently by a researcher not involved with the follow up of participants.

As is usual with embedded trials, the sample size is constrained by the number of patients actively participating within the host trial, hence a formal power calculation to determine sample size has not been conducted.

The primary outcome of this embedded trial will be the proportion of participants who return their questionnaire in each group; time to response, whether a reminder notice is required, completeness of response, and cost of the intervention per participant retained will serve as secondary outcomes.

Retention SWAT 2

We will evaluate the effect of including a pen on retention rates. Participants will be randomised to receive 1) a pen; 2) no pen. These will be sent at Month 3 following randomisation to the VenUS 6 trial.

Effective low-cost strategies to improve retention in trials are needed. Growing evidence supports providing pens along with postal questionnaires to encourage continued trial participation. Previous studies found that including a pen could increase response rates [29] and may elicit responses from participants who had previously failed to return questionnaires [30]. Similarly, more recent evidence indicates a favourable effect on completion rates, along with a reduction in the time taken to return questionnaires [28]. Further, the inclusion of a pen might reduce the number of reminders needed to prompt questioner completion [27]. These findings suggest that pens could act as a sufficient non-monetary incentive to increase retention in trials.

All participants recruited into the VenUS 6 trial, and who remain as fully participating (i.e. have not fully withdrawn, withdrawn from postal follow up or have died) will be eligible for the SWAT. There are no additional inclusion or exclusion criteria. Allocations will be stratified by main trial allocation. Generation of the allocation sequence will be undertaken independently by a researcher not involved with the follow up of participants.

As is usual with embedded trials, the sample size is constrained by the number of patients actively participating within the host trial, hence a formal power calculation to determine sample size has not been conducted.

The primary outcome of this embedded trial will be the proportion of participants who return their questionnaire in each group; time to response, whether a reminder notice is required, completeness of response, and cost of the intervention per participant retained will serve as secondary outcomes.

SWAT 4: Exploring the feasibility of study participants self-reporting wound healing

We will explore whether study participants with venous leg ulcers are willing and able to self-report wound healing as a research outcome and whether some people are more willing to self-report

than others. This information will be used to inform the design of future research, it will not impact on how clinical data are collected for VenUS 6.

Currently, healing data in RCTs of venous leg ulcers are collected and reported by healthcare practitioners. However, as venous leg ulcers can take several months to heal, data collection can be costly and burdensome to research teams [31]. It is possible that implementing participant self-reporting of healing outcomes can reduce the costs of monitoring venous leg ulcers until complete wound closure occurs. The feasibility of this approach has not been investigated before in this population.

All VenUS 6 participants will be eligible for this nested study if they are still to receive a 3-month questionnaire (i.e., have not past this point in their follow-up, have not fully withdrawn, withdrawn from postal follow-up, or have died). There are no additional inclusion and exclusion criteria.

Participants have provided informed consent to receive questionnaire data as part of the main trial, and therefore will also receive this brief survey, asking participants about whether they are able and willing to assess their wound and how they would prefer to report changes in its status, as part of the main VenUS 6 3-month questionnaire pack. Consent will be assumed by virtue of survey completion and return to the coordinating centre.

4. Study Population

We will include adult patients with at least one venous leg ulcer who fulfil all of the following Inclusion Criteria and none of the Exclusion Criteria.

All individuals will be considered for inclusion in this study regardless of age, disability, gender reassignment, marriage and civil partnership, pregnancy and maternity, race, religion and belief, sex, and sexual orientation except where the study inclusion and exclusion criteria EXPLICITLY state otherwise.

4.1 Inclusion criteria

- has at least one venous leg ulcer[1]
- An ankle–brachial pressure index (ABPI) of ≥ 0.8, taken within the previous 3 months or, where an ABPI measure is not viable, use of locally-approved alternative assessments to rule out peripheral arterial disease i.e. pulse palpation and doppler ausculation, toe pressure assessment or arterial imaging, also taken within the last 3 months.
- is able to tolerate full compression
- is aged ≥ 18 years

[1]For the purpose of this study a venous leg ulcer will be considered as any break in the skin on the leg which has either (a) is venous in appearance (i.e., moist, shallow, irregularly shaped ulcer in the gaiter area) and accompanied by signs of chronic venous disease e.g., ankle flare, lipodermatosclerosis, varicose veins, hyperpigmentation, atrophie blanche; or (b) occurs in a person with a history of venous leg ulceration. A participant will be considered to have a

purely venous leg ulcer where clinically no other aetiology is suspected. Clinical history must be considered and the study participant must have an ABPI of equal to or greater than 0.8.

The venous leg ulcer must lie wholly or partially within the gaiter region of the leg; venous leg ulcers which lie partially within the gaiter region and also extend onto the foot will be permitted, however, venous leg ulcers which are confined to the foot only will not be eligible for inclusion within this trial.

4.2 Exclusion criteria

- is not willing to wear full compression
- has leg ulcers of non-venous aetiology (e.g., arterial or rheumatoid) or significant peripheral vascular disease which contraindicates full compression (requiring an APBI of at least 0.8)
- has ulcers confined to the foot
- lacks capacity or willingness to provide consent to participate in the trial
- is currently participating in another study evaluating treatments for their venous leg ulcer
- has known allergy to any trial product
- has been previously recruited for this trial
- is deemed to be not clinically appropriate to take part in the trial (at clinician discretion)
- Planned treatment to close/remove incompetent superficial veins (e.g. via endovenous ablation, sclerotherapy) within 28 days

5. Recruitment, randomisation and withdrawal processes

5.1 Identification of potential participants and recruitment

Potential participants on clinical caseloads at trial sites will be screened against the eligibility criteria; those eligible will be approached about the trial and given study details, including an information sheet. The initial in-person approach will be by a member of the clinical care team.

It will be clearly stated that the potential participant is free to withdraw from the study at any time for any reason without prejudice to future care, and with no obligation to give the reason for withdrawal. New information arising during the study which may affect a participant's willingness to take part in VenUS 6, e.g. findings from another study, will be reviewed for possible addition to the patient information sheet and consent form. We are not aware of any studies being conducted which are likely to generate results which will impact in this way.

The potential participant will be allowed as much time as they wish to consider the information, and given the opportunity to question the Principal Investigator, the research team, their GP or other independent parties to decide whether they will participate in the study. Potential participants will then be shown the consent form again and will be given the opportunity to ask questions about the study.

Informed consent will be obtained by a suitably qualified and experienced member of the research or clinical care team, as agreed by the participating site R&D group and/or Sponsor if required, and who has been authorised to do so by the Chief or Principal Investigator, as detailed on the study Delegation of Authority and Signature Log for the study site.The participant must personally sign and date the latest approved version of the informed consent form before any study specific, baseline procedures are performed. Part of the consent process

will also be to ask for consent to potentially follow-up participants beyond the end of the trial for a maximum of five years. This is to allow future longer-term research to be considered. The consent form will also ask participants if they would like to take part an interview exploring views and experiences of compression therapies. This is optional and not participating in the interviews will not affect main trial participation.

The original signed form will be retained at the study site within the Investigator Site File (ISF); while copies will be given to the participant, retained in the participant's medical notes, and provided to the study coordinating centre.

Once informed consent has been obtained, baseline data will be collected (see Section 7.4 for data storage details). This will include:

Participant details

- Contact details for the participant;
- Details of participant's GP;
- Diabetes status; weight, height and general mobility and ankle mobility.

Ulcer history and assessment:

- Duration and area of the reference ulcer;
- Number of previous ulcer episodes;
- ABPI reading using an established technique (or record of an assessment carried out within the last 3 months);
- Ankle circumference.

Self-completed participant data (see section 7.2 for full details of measures used)

- VEINS-Qol;
- EQ-5D-5L;
- Ulcer-related pain;
- Resource use.

Baseline Photo

- A photo, or photos depending on size, taken of the reference ulcer

5.2 Definition of reference ulcer and reference leg

Where a participant has multiple venous ulcers, the eligible ulcer with the largest surface area (cm^2) will be termed the **reference ulcer** and assessed for time to healing. The leg on which the reference ulcer is located will be termed the **reference leg.** Where participants have bilateral leg ulcers we will assume that the leg with the reference ulcer is the leg with the worst prognosis.

5.3 Randomisation

Following collection of baseline data, the research team will contact the UKCRC-accredited University of York Trials Unit (YTU) via the internet to access a secure randomisation service, which will ensure complete allocation concealment. The randomisation service will record information and check eligibility to avoid inappropriate entry of patients into the trial. Randomisation will be stratified by ulcer duration (≤ 6 months and > 6 months) and ulcer area (≤ $5cm^2$ and > $5cm^2$) using permuted blocks; these variables are known predictors of healing.

Once randomised, participants will begin their trial treatment as soon as it is available in line with what would happen in routine practice. As a result, receipt of other treatments, due to lack of

availability of the allocated treatment immediately following randomisation will not be treated as non-adherence but will be reported descriptively.

5.4 Participant withdrawal and trial exit

Each participant has the right to withdraw from the study at any time without prejudice. In addition, the investigator may advise that a participant be discontinued from the study at any time if the investigator considers it necessary for any reason; however withdrawal decisions remain with the participant at all times.

The reason for withdrawal will be recorded within study documentation. If the participant is withdrawn due to an adverse event, the Investigator will complete follow-up visits or telephone calls until the adverse event has resolved or stabilised.

Participants who request to withdraw will be asked which study elements they would like to withdraw from. This could be: (1) clinical data collection, (2) data collection via postal questionnaires or (3) all trial activities. If deemed appropriate participant will be asked if the research team can try to obtain primary outcome data through contact with healthcare professionals only. Where full withdrawal is requested this will be termed trial exit. Trial exit means that participants leave the trial and no further data are collected from participants. This can happen at the end of the trial follow-up period or upon full withdrawal

We will ask if those withdrawing are happy for their personal details to continue to be stored as agreed in their original consent: if this is refused all identifiable data will be destroyed.

Where participants lose capacity to consent during their time in the study, they will be withdrawn from further follow up; however data collected until this point will be retained for use. No further data would be collected or any other research procedures conducted in relation to the participant.

6. Trial Treatments

6.1 Compression wraps

Relevant compression wraps are defined by their use of hook and loop (Velcro™) to secure a compression sleeve around the foot and leg. These sleeves are pulled tight with the aim of delivering strong compression (≥40mmHg) at the ankle, the therapeutic level of compression required.

Compression wraps which fulfil the following specification can be used in the trial:

- An adjustable compression sleeve secured with hook and loop fastenings
- Designed to be worn on the lower leg and foot in people with venous leg ulceration (note: wraps must be marketed specifically for this purpose)
- Aim to deliver strong (≥40mmHg) compression at the ankle with a system to guide this to support self-application
- Treatment is CE marked and available on FP-10

These may be used with a compressive or non-compressive liner and with use of foot compression wraps. The use of these elements of the treatment is at the discretion of the treating health professional as long as the above criteria are met.

Compression wraps currently used for the treatment of lymphedema will not be included in this trial unless they are specifically marketed, with corresponding training and instruction for use in people with venous leg ulcers.

The compression wraps will be used according to manufacturers' instructions. Training for the use of the products will be supported by the manufacturers as per standard practice. We will ensure all sites have received this training.

6.2 Two-layer compression bandage

Two-layer bandage systems are composed of an initial bandage layer covered with a top cohesive compression bandage. Together these two-layers are designed with the aim of delivering strong (≥40 mmHg) compression at the ankle. We will use any recognised two-layer bandage kit system that aims to deliver strong (≥40 mmHg) compression at the ankle. Kit systems deemed to be appropriate for use are K-Two (Urgo), Coban 2, Andoflex and Actico2c. Other two layer banadage kit systems will be considered on a case by case basis by the trial team prior to use.

All nurses and other relevant health professionals are likely to have received training in the use of two-layer bandaging. However, refresher training will be organised with the relevant manufacturer if required.

6.3 Evidence-based compression (compression hosiery or the four-layer bandage)

Given the results of VenUS IV [4,5] we have opted for a 'choice' comparator arm where allocated participants are offered two-layer compression hosiery, if they are deemed suitable, or otherwise the four-layer bandage. The treatment decision will be based on clinical judgement and patient preference. This is what would happen in practice and fits with the pragmatic nature of the proposed trial.

We will use any recognised four layer system that aims to deliver strong (≥40 mmHg) compression at the ankle. We will allow sites to use their normal four layer system and standard application procedures will apply.

Compression hosiery will consist of a two layer compression system aiming to deliver sustained, graduated compression of ≥40mmHg at the ankle. The first layer is an understocking or liner providing light compression over which a second overstocking (i.e. UK class 2 or 3 depending on the understocking) is easily applied.

The size of the two layer hosiery kit used will depend upon the participant's ankle circumference (cm), calf circumference (cm) and/or foot length (cm); nurses will consult product-specific measurement tables to ensure the participants received the correctly sized kit. Made to measure two layer hosiery can be obtained for participants who require this. Two layer hosiery will be given with advice to apply and wear according to manufacturer's instructions.

6.4 Primary contact layer

To ensure the pragmatic nature of the study, nurses will be able to use any primary contact dressings under the bandaging or dressing. Dressings used will be recorded by type.

7. Data collection (excluding adverse event data, see section 8)

7.1 Staff-collected data

Staff will phone the participants on a monthly basis to monitor the healing of the reference ulcer, the reference leg (if the ulcer has healed), participant trial status and any clinical events experienced.

Healing of the reference ulcer

The primary outcome of this trial is time to healing of the reference ulcer, defined as: complete epithelial cover in the absence of a scab (eschar) with no dressing required.

Treating nurses will be asked to report the date when they consider the reference ulcer and the reference leg has healed. Additionally, data on the reference ulcer will be collected throughout the study in the form of digital images taken by the treating nurse or participant. A digital image will be taken at baseline and again when the treating nurse records the reference ulcer as healed. After this point images will be taken once a week over the next 4 weeks.

Wherever possible the healing visits will be completed face to face, by the research nurse, with the participant at home, or in a clinical care setting if preferred.

If face to face contact cannot be facilitated for a visit (e.g. due to patient availability, local or national government guidelines), NHS approved video call technology should be used to undertake assessment, with a screen shot taken of the ulcer.

If video call technology cannot be used then a telephone call will be undertaken to assess healing, with the participant asked to take and return a photograph of their ulcer. Where participant are asked to take a photograph themselves, study specific instructions will be provided and they will be encouraged to ask a friend or relative to assist with this should the ulcer location mean it is difficult for them to take the photograph independently.

These images will be assessed by two clinical experts blinded to allocation to confirm the date of healing, with disagreements being resolved through discussion and the involvement of a third reviewer if required. The blinded assessment of healing date will be used as the primary healing endpoint. Non-blinded assessment of healing will be used as a secondary outcome.

Treatments-received (including changes to trial treatment)

We will log every wound-related nurse visit to the participant, using a case report form, until either the participant's reference leg is ulcer free and no more nurse visits are required to treat this leg or until the participant exits the trial. We will record the date of visit, the type of compression being received and the type of primary contact dressing being used. These resource use data will be used in the economic analyses.

We will record if a participant changes from their allocated compression treatment, the date of this change, what they changed to and the reason for this change; including whether the change from trial treatment was requested by the patient or based on a clinical decision.

Healing of the reference leg

Healing of the reference leg will be monitored through monthly telephone calls to the participant by the local research team. Calls are to be continued following the healing of the reference leg to assess for recurrence, as detailed below. Although the clinical analysis defined the primary outcome based on healing of the reference ulcer, the clinically relevant outcome is healing of all ulcers (ulcer free patient). We will assume that the reference leg is independent of the non-reference leg (even if this has ulcers), and that healing of the reference leg is the main outcome for evaluation in the economic analysis.

Recurrence

Following healing of the reference leg we will undertake a monthly telephone assessment for ulcer recurrence until the participant exits the trial. The maximum period for trial follow-up will be 12 months following randomisation, although due to variable follow-up some participants will be followed for less time (minimum 4 months).

Clinical Events

Throughout the study we will record clinical events including ulcer/skin deterioration, ulcer infection, amputation, admission/discharge, planned treatment to close/remove incompetent superficial veins (e.g. via endovenous ablation, sclerotherapy) within 28 days, infection, new ulcer occurrence or death. The local research team will contact the participant on a monthly basis by telephone to collect this information. The maximum period for trial follow-up will be 12 months following randomisation, although due to variable follow-up some participants will be followed for less time (minimum 4 months).

7.2 Participant self-reported data

Trial participants will be sent a postal questionnaire, by York Trials Unit, at regular intervals during follow up. Participants will be provided with a freepost envelope to facilitate return of the completed questionnaire to York Trials Unit for processing.

Where no response is provided to a questionnaire, a reminder letter will be sent to the participant after two weeks to encourage completion and return of the questionnaire; this has been shown to increase the likelihood of response [18]. Where no response is provided after a further two weeks, participants may be contacted by telephone during which, if agreeable, the CRF will be completed.

A systematic review investigating ways of increasing questionnaire response rates [18] reported that response to postal questionnaires was doubled (odds ratio = 2.02; 95% confidence intervals 1.79 to 2.27) when a financial reward was included with the questionnaire, versus no incentive. The response rate increased further when the incentive was not conditional on response, versus upon return of questionnaire. Based on these data we will include £10 with the final questionnaire sent to participants at 12 months. In their 6 month letter participants will be told of this unconditional token of our appreciation for the time they have taken to complete documentation (at 3 months for variable follow-up).

Healing of the reference ulcer

Treating nurses will be asked to report the date when they consider the reference ulcer and the reference leg has healed. A baseline digital image will be taken and once the treating nurse records the reference ulcer as healed, once a week over the next 4 weeks. To ensure all photos

are similar, are good quality and anonymised a robust photography protocol, and associated training, will be established.
Where it is not possible for the nurse to take post healing images, for example due to participants having moved locality during the study or national guidelines prevent face to face assessment, we will ask participants to take and return a photograph of the wound themselves. Study specific instructions will be provided to the participant, and they will be encouraged to ask a friend or relative to assist with this should the wound location mean it is difficult for them to take the photograph independently.

Health related quality of life and utility data (participant self-report at baseline, 3, 6, and 12 months)
Health related quality of life will be assessed, at baseline, 3, 6 and 12 months using the VEINES-QoL which has been found to be more responsive to ulcer healing than the SF12. [19]

The EQ-5D-5L will also be used, at Baseline, 1, 3, 6 and 12 months to assess health related quality of life. The EQ-5D-5L measures impairment level to five dimensions: mobility, ability to undertake usual activities, pain and discomfort, anxiety and depression. The EQ-5D-5L will allow for the implementation of the cost-effectiveness analysis (e.g. estimation of quality-adjusted life years) in conjunction with the resource use questionnaire.

Resource use participant self-report at 3, 6, and 12 months
Participants will be asked to provide details of any care received from the NHS within the past 3 months, recording the number of consultations the participants had with health professionals at different locations. Participants will be asked to record details according to whether the consultation was related to their leg ulcer or a different reason.

Wound-related pain participant self-report at baseline, 1, 3, 6, and 12 months
Participants will be asked to rate the intensity of any leg ulcer-related pain that they have experienced in the previous 24 hours using the 21-point Box Scale (BS-21). The BS-21 pain scale will be divided into units of five, and ranged from a value of 0 (no pain) to 100 (the worst pain imaginable).

Treatment adherence and ease of use (month 1, 3, 6 and 12 months)
Participants will be asked to complete compression treatment questionnaires as part of their self-report postal questionnaires to assess views on the compression treatments, volume of treatment use and reasons for reduced dose (partial adherence).

A summary of all data collection is presented in Table 1 (as used in previous VenUS trials).

	**Enrolment**	**Allocation**	**Clinical Assessments**		**Postal Questionnaires**				**GP check**
TIMEPOINT	Pre-randomisation/ Baseline	Randomisation	Participant Visit Log (weekly)	*Participant Events Form (Monthly and when required)	1 mon	3 mon	6 mon	12 mon	
ENROLMENT									

Eligibility screen	✓								
Informed consent	✓								
Baseline data	✓								
Allocation		✓							
STAFF COLLECTED									
Wound Healing			✓	✓					
Changes to treatment			✓						
Other clinical events			✓*	✓					
Adverse events			✓	✓					
Confirmation of death				✓					✓
PARTICIPANT REPORTED									
HRQOL,	✓				✓	✓	✓	✓	
Resource use						✓	✓	✓	
Wound-related pain; Participant adherence; Ease of treatment use					✓	✓	✓	✓	

Table 1: Summary of trial-based data collection

7.3 Additional data collection

Participant death (following trial exit)

For the assessment of incremental costs and Quality Adjusted Life Years (QALYs) it is important to know if participants are alive at the end of the trial. Where people are ulcer-free this on-going monitoring is particularly important as the nurses will stop treating the participant. Through the monthly telephone calls to participants, local research nurses will identify and notify York Trials Unit if any trial participants have died during this period. To ensure we have this information at the end of the trial, if a nurse is unsure about the status of a healed participant, we will contact the participant's GP, offering a per participant payment to the practice for the provision of this information. This process will be consented to at randomisation and will not occur in any participants who do not consent to this element of data collection.

Understanding participant use of compression treatments ***(process evaluation)***

Data collection to explore duration of compression use and calculation of resource use are discussed above. Here we focus on the methods planned to assess compression delivery, use and adherence.

To more fully understand the findings of the trial and expand knowledge about how people view and use compression especially systems such as wraps that allow self-application and self-removal, we will conduct semi-structured interviews of approximately 20 participants randomised to different compression treatments.

As we are collecting more data on other compression treatments in a current research project (VenUS V), for the VenUS 6 interviews we may over recruit participants using compression wraps.

Participants will be asked to complete compression treatment questionnaires as part of their self-report postal questionnaires to assess views on the compression treatments, volume of treatment use and reasons for reduced dose (partial adherence).
Using this data from the one and three month self-report questionnaires and subject to participant consent, we will select a purposive, maximum variation sample according to gender, age, co-morbidities, treatment and ulcer history. The final sample size will be determined by concurrent data analysis and saturation of emerging themes. Participant interviews will take place between 1 and 9 months.

The interviews will use topic guides developed from reviews of the literature on quality of life of people living with venous leg ulcers and consultations with our PPI representatives. The topic guide will explore compression adherence, ease of application, reasons for treatment change, and experiences of wearing different compression treatments. It will be iterative to allow any new themes that arise during interviews to be explored with subsequent participants. Interviews will last around an hour and will take place over telephone or preferred contact method of the participant.

We will also conduct up to 10 individual interviews with nursing staff who deliver compression treatments to gather their views and experiences on treatment delivery of compression, especially compression wraps. Because we are collecting relevant staff data in another research study we have reduced the data to be collected here.To supplement this we will also conduct group interviews with site teams to understand local processes for ordering and delivery of compression therapies.

The interviews will be audio-recorded and fully transcribed. Interview data will be entered into NVivo 9.0 and analysed using a framework analysis [21].

7.4 Source data definition, transfer and storage

Source documents are original documents, data, and records from which participants' study-specific data are obtained. These include, but are not limited to, nurse records (from which medical history and previous and concurrent medication may be summarised into the study-specific documentation), clinical and office charts, diaries, correspondence, completed scales and quality of life questionnaires. Study documentation entries will be considered source data if the form is the site of the original recording (e.g., there is no other written or electronic record of data). In this study the study documentation will be used as the source document as outlined in the Source Data Verification form.

All documents will be stored safely in confidential conditions. Any paper forms containing participant identifiable information (e.g. patient details form and consent form) will be held in a location separate to the questionnaire data. Identifiable information held by York Trials Unit will be stored securely in a locked filing cabinet, in an office only accessible via registered swipe card access held by the York Trials Unit research team (as per YTU Standard Operating Procedure YT03).

Personal data held electronically, will be stored on the study specific participant management system which will record identifiable information and participant activity to enable study coordination. The study specific participant management system will be developed by the York

Trials Unit Data Management team for the purposes of this study. The system will be housed on York Trials Unit, University of York servers, which are secure and is subject to rigorous testing and continued backup. Sites will have access to the system, via individual password, to facilitate randomisation and permissions for access will also be detailed within the study delegation log. The study team based at York Trials Unit will have access to the system, via individual password, to facilitate study conduct including study coordination and the management of administering and receiving postal questionnaires. Permissions for access will also be detailed within the study delegation log

Photographs collected to record participant ulcer healing at the three post ulcer healing assessment visits will be anonymised prior to electronic transfer by sites, via the NHS Approved Digital Encryption guidance, to the University of York, where they will be stored in an encrypted and password protected drive. All data will be stored in accordance with data protection principles. On all study-specific documents, other than the signed consent and participant contact details form, the participant will be referred to by the study participant number/code, not by name.

7.5 Level of blinding

By the nature of the timing of the study treatments used within this study, blinding of the participants and clinicians is not possible and procedure for un-blinding is not necessary.

7.6 Final Study Data Set

Following completion of the VenUS 6 trial, and associated analyses, the data set will be held at York Trials Unit. This data set will be accessible subject to a completed York Trials Unit Data Request form, and Chief Investigator confirmation. The data set will be held in Stata format (as a .dta file), on the York Trials Unit, University of York servers, which are secure and are subject to rigorous testing and continued backup.

8. Adverse Events

8.1 Definition of adverse events

For the purposes of the VenUS 6 trial, adverse events are defined as any untoward medical occurrence (i.e. any unfavourable and unintended sign, symptom or disease), experienced by a clinical trial participant and which is temporally associated with study treatment (interventions or control) and is related to the ulcer or to the study intervention or control treatments.

Adverse events, which might be expected with these wounds include:

- Skin maceration
- Ulcer deterioration
- Bandage-/hosiery-related pain/discomfort
- Medical event relating to leg
- Occurrence of new ulcer
- Skin damage
- Ulcer-related pain

For the purposes of the VenUS 6 trial the following events do **not** need to be reported as an Adverse event:

- Wound-related infection
- Dryness
- Excoriation
- Skin deterioration

8.2 Serious Adverse Events

To ensure no confusion or misunderstanding of the difference between the terms "serious" and "severe", which are not synonymous, the following note of clarification is provided:

The term "severe" is often used to describe the intensity (severity) of a specific event (as in mild, moderate, or severe myocardial infarction); the event itself, however, may be of relatively minor medical significance (such as severe headache). This is not the same as "serious," which is based on patient/event outcome or action criteria usually associated with events that pose a threat to a participant's life or functioning. Seriousness (not severity) serves as a guide for defining reporting obligations.

Serious adverse events (SAE) are defined as any untoward medical occurrence that:

1) Results in death
2) Is life threatening

NOTE: The term "life-threatening" in the definition of "serious" refers to an event in which the participant was at risk of death at the time of the event; it does not refer to an event that hypothetically might have caused death if it were more severe.

3) Requires unplanned inpatient hospitalisation or prolongation of existing inpatients' hospitalisation
4) Results in persistent or significant disability or incapacity
5) Is a congenital anomaly or birth defect
6) Any other important medical condition that, although not included in the above, may require medical or surgical intervention to prevent one of the outcomes listed.

For the purposes of the VenUS 6 trial hospitalisation for treatment for wound infection, to prevent/resolve skin deterioration, or medical events relating to the reference leg, will **not** be considered a SAE but will be reported using the VenUS 6 Adverse Event Form.

8.3 Reporting Procedures for Adverse and Serious Adverse Events

Adverse events should be entered onto the Adverse Event reporting form and reported to York Trials Unit within 5 days of discovery or notification of the event.

SAE should be entered onto the Serious Adverse Event reporting form and reported to York Trials Unit within 24 hours of discovery or notification of the event. Once received, causality and expectedness of SAE will be confirmed by the Chief Investigator or another clinical member of the Trial Management Group (if the CI is unavailable). Summaries of adverse and serious adverse events will be provided to the Trial Steering Committee and Data Monitoring Committee at their scheduled meetings.

SAEs that are deemed to be unexpected and related to the trial will be notified to the Research Ethics Committee (REC) and Sponsor within 15 days. Alongside this, all such events will also be reported to the Trial Steering Committee and Data Monitoring Committee for their consideration..

All events will be followed up until the event resolves or a decision is made for no further follow up. Participants experiencing SAEs which are deemed to be related to the trial treatments (intervention or control) and which remain ongoing at the time of participant trial exit will be followed up for one further month beyond trial exit.

Where repeated adverse events (serious or non-serious) of a similar type are observed, these will be discussed with the Data Monitoring Committee (DMC) and will be onward reported to the REC and Sponsor should concerns be raised in relation to the type of event and/or frequency observed.

9. Statistical and Economic Considerations

9.1. Sample size for recruitment

The sample size calculation for this study is based on parameters from the HTA funded VenUS I [7,8] and VenUS IV trials [4,5]. The hazard ratio in VenUS I was 1.33 (95% CI 1.05 to 1.67) and the proportion of people with a healed reference ulcer (blinded assessment) in VenUS IV was 70%. A hazard ratio of 1.33 will be used as the non-inferiority margin for the comparison of 2LB and EBC, with the sample size for the superiority comparison also being based on this difference. 675 participants (225 in each group) are required to obtain 80% power (non-inferiority comparison) and 90% power (superiority comparison) in two-sided tests of size 5%, assuming a median time to healing of 2.3 months, an average follow up period of 12 months and 10% attrition. The superiority comparison assumes that the 2LB is found to be non-inferior to EBC and that these groups are combined and compared with wraps (i.e. 2:1 allocation ratio). If 2LB is found to be inferior (i.e. there is insufficient evidence to reject the null hypothesis for the non-inferiority comparison), then we will still retain 80% power for the superiority comparison of EBC or 2LB with wraps (ceteris paribus).

9.2 Internal pilot

The first six months of recruitment will constitute an internal pilot phase and will be evaluated on the following predefined criteria to ascertain our ability to recruit and randomise. Assumptions will be assessed against pre-defined 'traffic light' stop go criteria (Table 2).

	GO	DISCUSSION REQUIRED	STOP
**Site set-up**	8 or more sites set up	4 or more sites set up	Fewer than 4 sites set up
**Participant randomisation**	43 or more participants randomised	25 or more participants recruited	Fewer than 25 participants recruited
**Centre recruitment**	≥80% of centres recruiting to target of 2 participants a month	≥60% of centres recruiting to target of 2 participants a month	Fewer than 50% of centres recruiting to target of 2 participants a month
**Treatment adherence**	At least 70% of participants allocated to the wrap arm are wearing allocated treatment for 7 days or more	At least 40% of participants allocated to the wrap arm are wearing allocated treatment for 7 days or more	Less than 40% of participants allocated to the wrap arm are wearing allocated treatment for 7 days or more
**Feasibility of follow up**	80% or greater response rate to 3-month questionnaire	70% or greater response rate to 3-month questionnaire	Less than 70% response rate to 3-month questionnaire

Table 2: Proposed stop/go criteria. Mean participant recruitment target is 1.2 participants per site per month. Sites will be given a target of two participants to recruit a month.

Recruitment assumptions and intervention rate will be assessed initially at 3 months, and again at 6 months. We aim to support the quantitative data for the stop/go criteria with further information gleaned from staff which might help understand possible issues and how to mitigate these. If there is an issue with recruitment relating to one arm of the trial but not the others we will discuss with the Trial Steering Committee who will bring a recommendation to the HTA board in terms of further options.

9.3 Full-trial analyses

The trial analyses will follow a detailed pre-specified statistical analysis plan (SAP), that will be approved and published before the end of patient recruitment. The primary outcome will be time to healing of the reference ulcer as defined in Section 7.1. Participants who are lost to follow up prior to healing of the reference ulcer, will have healing times censored at the time of their last study contact. Participants who reach the end of study follow up prior to healing of the reference ulcer, will have healing times censored at this time point. For the primary analyses, participants who experience competing events (death or amputation of the reference leg) prior to healing of the reference ulcer, will have healing times censored at the time the competing event occurred (the possible influence of competing events will be investigated in sensitivity analyses using competing risk regression methods).

The primary analyses will include both non-inferiority and superiority comparisons. For the non-inferiority comparson (2LB vs EBC), two treatment effect estimands will be targeted[1]. An unconstrained treatment policy estimand whereby all intercurrent events (e.g. treatment switches/cessations and receipt of surgery) will be accepted as part of completely pragmatic treatment policies (essentially an intention-to-treat analysis); and a constrained estimand whereby certain constraints are placed on the intercurrent events that are accepted as a part of the treatment policies.

Intercurrent events (such as treatment switches) will generally result in the care received by patients allocated to different treatment groups being more similar than intended, potentially resulting in attenuation of differences between treatment groups. Hence estimands that do not address the influence of intercurrent events are potentially anti-conservative in the context of investigating non-inferiority. This provides the rationale for the constrained treatment policy estimand; the purpose of this estimand is to remove the influence of intercurrent events that could either be mitigated in practice (e,g, treatment switches that could be avoided by appropriate use of analgesia), or that might not be applicable to all patients (e.g. surgical treatments that some patients cannot/will not receive), while accepting the influence of intercurrent events that cannot be easily avoided as an unalterable part of the treatment strategies being compared (e.g. treatment switches due to allergy/adverse events). For the superiority comparisons, just the unconstrained treatment policy estimand will be targeted. Precise description of the unconstrained and constrained treatment policy estimands, and the estimators/methods that will be used to estimate them, will be given in the SAP.

---

[1] An estimand is a precise descrioption of the treatment effect to be estimated with regards to five key attributes; (1) Population of interest, (2) Treatment strategies being compared, (3) Outcome of interest, (4) Treatment effect summary measure, (5) Handling of intercurrent events [REF]).

For the non-inferiority comparison, the unconstrained treatment policy estimand will be estimated by modelling reference ulcer healing time using a Cox proportional hazards model with a three level variable for group allocation (EBC vs 2LB vs CW), conditioning on the following baseline covariates; reference ulcer area, reference ulcer duration, participant age, participant mobility status and recruitment site (via shared frailties for participants recruited at the same site). For the constrained treatment policy estimand, patients will have their reference ulcer healing times censored at the first of any relevant departures from the constrained treatment policies. These healing times will then be analysed using a similar model as used to estimate the unconstrained treatment policy estimand. For both analyses, the point estimates and 95% confidence intervals of the Hazard Ratios (HRs) for all between group contrasts will be reported, together with estimated differences in median healing times (with 95% confidence intervals based on non-parametric bootstraps).For each estimand, the estimated upper 95% confidence limits of the HRs for the EBC vs 2LB contrasts will be compared with the non-inferiority margin of 1.33. If either of these upper limits is greater than 1.33, then the null hypothesis that 2LB is inferior to EBC will not be rejected. If both estimates are less than 1.33, then the null hypothesis that 2LB is inferior to EBC will be rejected.

If the null hypothesis that 2LB is inferior to EBC is rejected, then a further model will be fitted with the 2LB and EBC groups combined and compared with CW (i.e. a two level variable for group allocation – EBC+2LB vs CW), with the same fixed effects and shared frailties as previously. The point estimate and 95% Wald method confidence interval of the HR for the 2LB+EBC vs CW contrast will be reported, together with the estimated difference in median healing time (with 95% confidence intervals based on non-parametric bootstraps).

Secondary time to event outcomes include time to healing of the reference leg and time to ulcer recurrence. Time to healing of the reference leg will be calculated and analysed in a similar manner to the primary outcome, although only the unconstrained treatment policy estimand will be targeted and there will be no combined 2LB and EBC vs CW comparison.

Time to recurrence is defined as time from an ulcer free reference leg to date of recurrence of a new ulcer on the reference leg. Participants whose reference leg heals and remains healed until they reach the end of follow up or are withdrawn will have their time to recurrence censored at the relevant time, as will participants who experience a competing event (mortality and amputation of the reference leg). This outcome will be analysed using a similar model as used for the analyses of the primary outcome, although there will be no combined 2LB and EBC vs CW comparison.

Other secondary outcomes include quality of life measures (VEINES and EQ-5D-5L), ulcer related pain, (at 3, 6 and 12 months) and adverse events. Descriptive statistics will be presented. Where appropriate continuous measures will be analysed using appropriate generalised linear regression models and categorical variables analysed using logistic regression or ordinal regression models. Summaries of the compression treatments received and adherence to the randomised treatment will be reported by allocation.

9.4 Evidence synthesis for primary outcome

Mixed-treatment comparison meta-analysis (also known as network meta-analysis) will be used to enable the simultaneous comparison of multiple treatments in a single synthesis model, this will be supported by a systematic search to identify any new studies which need to be included.

The healing data from the trial, together with data from other recent studies, if available, will be used to update our previous network meta-analysis. [4] Formal network meta-analysis assumptions of similarity, homogeneity, transitivity and consistency will be reassessed. In addition to summary data, the availability of patient level data for several treatment contrasts will enable the exploration of both between- and within-study heterogeneity and testing of different assumptions over the distribution of time to ulcer healing.

9.5 Cost-effectiveness analyses

We do not think that a within trial cost-effectiveness analysis is required for this study. We have previously used a modelling approach to extend the findings of the VenUS IV trial to explore the cost-effectiveness of all relevant compression systems in the treatment of venous leg ulcers.[4] It is more useful to decision makers and less burdensome to use the collected trial data to update this work.

Under a UK NHS and the Personal Social Services perspective [20], a decision analytic modelling approach will be taken. The decision model will aim at estimating the relevant costs and health benefits of all relevant compression systems, to fully inform judgements on the long-term cost-effectiveness of these interventions in the treatment of venous leg ulcers. The modelling exercise will aim to represent possible patient disease (or health) pathways and, by doing so, can incorporate multiple sources of evidences, extrapolating from limited data, and explore uncertainty over parameter values. In this specific case, the main advantage of using a decision analytic model is in allowing the compression systems here to be compared to the wider set of relevant high compression treatments by including external information to the trial. The planned decision analytic model will extend previous work and include data from the network meta-analysis outlined above. Based on the selected model, further literature searching will be conducted to identify evidence on the following categories of model parameters: health related quality of life/utility, costs and resource use, ulcer recurrence and mortality, to be used alongside current trial data.

Costs and health benefits (health utility measured using EQ-5D-5L) will be derived using regression approaches, allowing for key covariates and uncertainty in estimates. Alternative scenarios regarding the extrapolation of the primary outcome over the lifetime of the model and the evidence informing it will be explored. Alternative scenarios exploring the extrapolation of secondary outcomes such as wound recurrence will also be explored. Uncertainty in the evidence base used to populate the decision analytic model will be characterised using appropriate distributions and any uncertainty in the adoption decision demonstrated using probabilistic sensitivity analysis. The value of further data collection using value of information analysis will be established.

9.6 Process evaluation analyses

A thematic framework for analysis will be developed based on the topic guide [23]. Transcripts will be indexed (coded) line by line using the thematic framework, but remaining open to new themes that emerge [24]. The data will be entered so that coded extracts can be attributed to individual participants. Finally participants' views will be compared and contrasted, and the data mapped schematically. Throughout data analysis emerging themes will be presented to the PPI representatives and TMG for discussion. Rival explanations will be explored, and a consensus reached where there are discrepancies in interpretation [22]. Data from the qualitative interviews will be analysed separately and independently from outcome data analyses.

Following analysis we will integrate and interpret the quantitative and qualitative data alongside each other using a mixed methods matrix [24] to allow comparison and interpretation of the datasets.

9.7 SWAT analyses

Recruitment SWAT

The primary analysis will be the difference in recruitment rates between those receiving the infographic in addition to the PIL and those not receiving the infographic. This will be analysed using logistic regression adjusting for important covariates as fixed effects and site as a random effect.

The difference in the proportion of those responding to a recruitment invitation who received the infographic in addition to the PIL but who do not go on to be randomised, and those not receiving the infographic but who do not go on to be randomised will also be analysed using a similar model to the primary outcome.

The difference in cost per recruited participant between those offered the infographic and those not offered the infographic will be calculated. In addition to the direct costs of the infographic, it may also be necessary to include the cost of staff time spent administering the recruitment packs.

Retention SWAT 1

Primary analysis: The difference in retention rates at 6 months will be analysed using a logistic regression model including each intervention (thank you card and newsletter), treatment allocation and other important covariates as fixed effects and site as a random effect. Adjusted odds ratios and corresponding 95% CIs will be obtained from this model. The presence of an interaction between the two interventions will also be tested using an interaction term.

Secondary analysis: The difference in the proportion of participants requiring a reminder letter mailing will be analysed using a similar model to the primary outcome. The difference in completeness of responses at 6 months will be analysed using a linear regression model using a similar adjustments to the primary outcome.

The difference in cost per retained participant between those sent a thank you card and/or newsletter and those not sent the thank you card and/or newsletter will be calculated. In addition to the direct costs of the thank you card, newsletter and postage, it may also be necessary to include the cost of staff time spent administering the mail out (for example filling and labelling envelopes).

The secondary outcomes at 12 months will be analysed as described above for the 6 month outcomes.

Retention SWAT 2

Primary analysis: The difference in retention rates at 3 months will be analysed using a logistic regression model including each intervention (pen), treatment allocation and other important covariates as fixed effects and site as a random effect. Adjusted odds ratios and corresponding 95% CIs will be obtained from this model.

Secondary analysis: The difference in the proportion of participants requiring a reminder letter mailing will be analysed using a similar model to the primary outcome. The difference in completeness of responses at 3 months will be analysed using a linear regression model using a similar adjustments to the primary outcome.

The difference in cost per retained participant between those sent a pen and those not sent the pen will be calculated. In addition to the direct costs of the pen and postage, it may also be necessary to include the cost of staff time spent administering the mail out (for example filling and labelling envelopes).

The secondary outcomes at 12 months will be analysed as described above for the 6 month outcomes.

Exploring the feasibility of study participant self-reporting wound healing

Numeric data analyses

All questions will be analysed descriptively with number and percentage of participants' responses reported for each categorical question. Numerical data will be summarised as means with standard deviations. We will use a multiple linear regression model to explore whether factors might predict people's willingness to self-report healing outcomes.

Thematic coding of open-ended questions

Data in the open-ended questions about the barriers and facilitators of self-reporting of healing outcomes will be thematically coded and themes will be generated, based on the emerging data, using inductive codes. These themes will be merged into higher level categories and summarised narratively. This will be an iterative process and so further themes may emerge and higher categories will be reshaped throughout the analytical process.

10. Ethical Arrangements

10.1 Ethical Approval

The VenUS 6 trial will be conducted in accordance with the Clinical Trials Regulations (2004/1031) and will be subject to approval from the Research Ethics Committee and the Health Research Authority prior to study activity commencing. The study will be conducted in accordance with the UK Policy Framework for Health and Social Care Research [25] and Medical Research Council (MRC) Good Clinical Practice (GCP) Guidance. [26]

Before being enrolled in the VenUS 6 trial, participants must consent to participate after the nature, scope, and possible consequences of participating in the clinical study have been explained in a form understandable to them. The Investigator will not undertake any measures specifically required only for the clinical study until valid consent has been obtained.

A Patient Information Sheet (PIS) that includes information about the study and a consent form will be given to the participant. These documents will contain all the elements required by the

ICH E6 Guideline for GCP and any additional elements required by local regulations. The PIS will give a balanced account of the possible benefits and known risks of the interventions. It will state explicitly that quality of care will not be compromised if the participant decides to a) not enter the trial or b) withdraw their consent. We will make it clear that there is no obligation to participate.
Patients will be given the opportunity to ask questions and the nature and objectives of the study will be explained. At the time of consent, written informed consent must be confirmed by the personally dated signature of the participant and the person conducting the informed consent discussions.

- The original signed consent form will be retained in the study files. Other copies of the consent form are required:
  - One copy of the informed consent form will be sent securely to YTU (by secure fax or encrypted email) and filed in the Trial Master File.
  - One copy of the informed consent form will be kept in the patient's clinical notes where applicable. If a patient does not have clinical notes at the trial site, the informed consent document will be filed in a separate folder.
  - One copy will be given to the patient.

10.2 Risks and Benefits
Side effects for compression wraps, the two-layer bandage and EBC are uncommon, and both treatments are routinely used in the NHS for patients with venous leg ulcers. Risks to participants because of any of the treatments are not increased through trial participation.

10.3 Informing Participants of Potential Risks and Benefits
Informed consent will be obtained by the trained local research nurse or clinician using a detailed patient information sheet developed with the help of service users, which will explain the risks and benefits clearly. In the unlikely event that new information arises during the trial that may affect participants' willingness to take part, this will be reviewed by the Trial Steering Committee for addition to the patient information sheet. A revised consent form will also be completed if necessary.

10.4 End of Trial
The end of the VenUS 6 trial will be the Last Patient Last Visit (LPLV), defined as:
- Completion of final planned follow up assessment in the study (4 months or 12 months depending on participant follow up plan)
- Full withdrawal from follow up due to any reason

10.5 Retention of Trial Documentation
In line with the principles of Good Clinical Practice/UK Clinical Trials Regulations, essential Trial documentation will be kept with the Trial Master File and Investigator Site Files. This documentation will be retained for a minimum of 5 years after the conclusion of the trial to comply with standards of Good Clinical Practice, and Sponsor requirements.

Case Report Forms will be used to record all the information required from the protocol and will be stored for a minimum of 5 years after the conclusion of the trial (either as paper records stored in a secure storage facility either on or off-site, or electronically on a password protected server) in accordance with guidelines on Good Research Practice [26].

11. Trial Finance and Insurance

11.1 Trial Funding

The VenUS 6 trial is funded by the NIHR Health Technology Assessment (HTA) Programme (HTA Reference: NIHR128625).

The Schedule of Events Cost Attribution Tool (SoECAT) and Organisational Information Document (OID) approved by the Health Regulatory Authority details all related costings for the VenUS 6 trial.

All interventions are standard treatment options currently available in the NHS. We anticipate that there will be no excess treatment costs for these interventions.

11.2 Trial Insurance

The Clinical Negligence Scheme for NHS Trusts will provide insurance to cover for liabilities and prospective liabilities arising from negligent harm. Clinical negligence indemnification will rest with the participating NHS Trust or Trusts under the standard NHS arrangement.

12. Project Management

12.1 Trial Sponsor

The trial will be sponsored by Manchester University NHS Foundation Trust

12.2 Trial Management

York Trials Unit (YTU) at the University of York will manage the study. YTU will also provide quality assurance for trial processes through centralised monitoring of key trial procedures (e.g. consent and eligibility) and documentation completion, in addition to routine checks with sites with regards documentation held in Investigator Site Files.

Each site will have a site Principal Investigator (PI) who will be responsible locally for the study. All trial staff will be trained in the trial procedures by YTU during site set up, thereby meeting the Sponsors (and NIHR) standards. Where required by the NHS Trust site, trial staff will have current GCP certification.

To ensure continued engagement with, and to provide support to, participating research sites, bi-monthly meetings will be convened and all active research sites invited to participate. Meetings will include sharing best practice in relation to recruitment and retention, problem solving, and sharing any key information relating to the trial or to emerging evidence in relation to venous leg ulcers or associated treatments.

A Twitter account will also be used to provide updates on progress to study sites, and to engage the wider research community with the trial.

The trial manager, on behalf of the Chief Investigator, will submit and, where necessary, obtain approval from all relevant parties for all substantial amendments to the original approved documents. Regular progress reports will be submitted as required to the Funding Body.

12.3 Trial Management Group

A Trial Management Group (TMG) will monitor the day-to-day management of the trial including the detailed design, set up, initiation and supervision of the study. This will comprise the Chief Investigator (CI), all co-applicants, trial team at YTU, trial statistician, and trial health economist. A representative of the Sponsor will also be invited to attend. The group will meet bi-monthly (as a minimum) from the start of the study to the end of the pilot phase and quarterly thereafter to manage the detailed design, set up, initiation and supervision of the study.

12.4 Trial Steering and Data Monitoring Committees

Independent oversight of the study will be conducted by the Trial Steering Committee (TSC), who will monitor the progress of the trial and provide independent advice. The TSC will comprise of independent clinicians and health service researchers with appropriate expertise and an independent patient representative. The TSC meetings will also be attended by the trial statistician and the study Sponsor will be invited to attend.

The study will be regularly reviewed by the Data Monitoring Committee (DMC), comprising of independent clinicians and health service researchers with appropriate expertise. The DMC will monitor the data arising from the study and recommend whether there are any ethical or safety reasons why the trial should not continue.

Both the TSC and DMC will meet at regular intervals to provide project oversight to the trial.

12.5 Patient and Public Involvement (PPI)

Our PPI TMG member acts as an advocate for patients, providing advice and guidance to the wider research team on issues that require direct experience of having been treated within the NHS for a leg ulcer. This member will also be involved in convening a group of individuals to sit on a Patient Advisory Group (PAG) for the trial and will work closely the VenUS 6 PPI liaison contact to facilitate feedback from the PAG to the Trial Management Group (TMG).

Patient Advisory Group

We are keen to ensure the involvement of patients, with active and healed venous leg ulcers, and carers is integral to this study, and so will recruit patients and carers to the PAG via our PPI Forum. The trial PAG will input regularly to discuss issues raised by the research team which require the perspectives of patients and carers, for example, reviewing study documentation (lay summaries and related recruitment material, information sheets and consent forms, and other patient-facing material) intended for a public audience. We will also ask willing members of the PAG to code or validate some of the qualitative data. The PAG will determine how they will meet, for example whether this is in person or online (i.e. by teleconference or Skype), how frequently they want to meet and will be responsible for nominating their own chair person.

13. Dissemination and projected outputs

Results from this study will be written up and submitted to peer-reviewed journals, irrespective of the magnitude or direction of effect. A publications policy will be generated in advance to detail

authorship, acknowledgements and review processes for any publications arising from the VenUS 6 trial.

The executive summary and copy of the trial report will be sent to the National Institute for Health and Care Excellence (NICE) and other relevant bodies, including Clinical Commissioning Groups, so that study findings can be translated into clinical practice nationally. We will also work with the relevant National Clinical Director in the Department of Health to help ensure the findings of the trial are considered when implementing policy and will work with the Speciality Advisory Committees (SAC) to incorporate the findings into the training curriculum for clinicians who will undertake treatment of venous leg ulcers.

A summary of the study report will be produced and made available to participants, members of our patient advisory group and relevant patient-focused websites. Service users involved in the VenUS 6 patient advisory group will be asked to actively participate in the generation of this to ensure the results are easily accessible to patients.

VENUS 6 Trial Project Management Plan

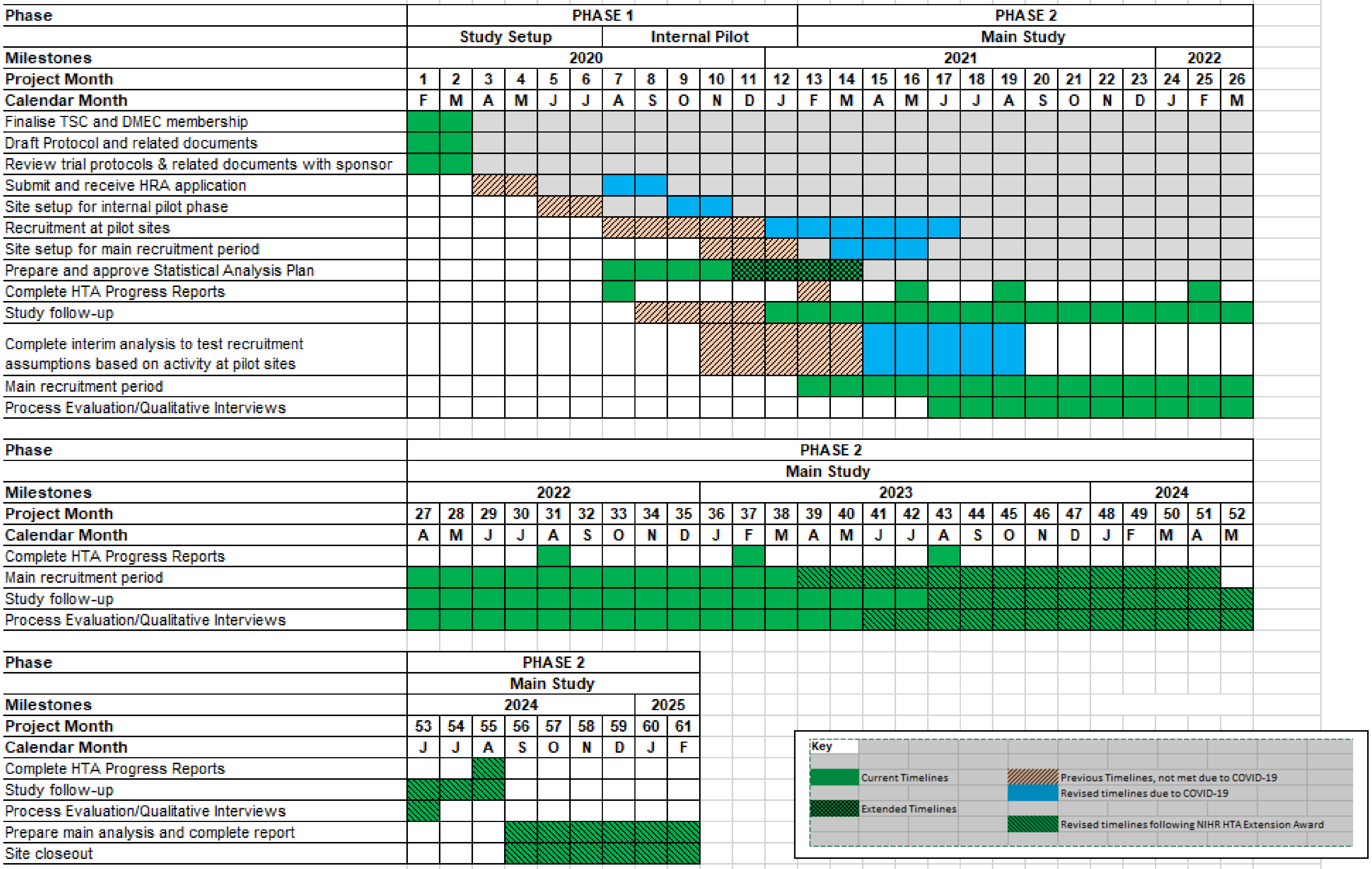

REFERENCE LIST

1. O'Meara S, Cullum N, Nelson EA, Dumville JC. Compression for venous leg ulcers. Cochrane Database of Systematic Reviews 2012, Issue 11. Art. No.: CD000265. DOI: 10.1002/14651858.CD000265.pub3.
2. Gohel MS, Heatley F, Liu X, Bradbury A, Bulbulia R, Cullum N, Epstein DM, Nyamekye I, Poskitt KR, Renton S, Warwick J, Davies AH; EVRA Trial Investigators. A Randomized Trial of Early Endovenous Ablation in Venous Ulceration. N Engl J Med. 2018 May 31;378(22):2105-2114.
3. Epstein DM, Gohel MS, Heatley F, Liu X, Bradbury A, Bulbulia R, Cullum N, Nyamekye I, Poskitt KR, Renton S, Warwick J, Davies AH; EVRA trial investigators. Cost-effectiveness analysis of a randomized clinical trial of early versus deferred endovenous ablation of superficial venous reflux in patients with venous ulceration. Br J Surg. 2019. doi: 10.1002/bjs.11082.
4. Ashby R, Gabe R, Ali S, Saramago P, Chuang L-H, Adderley U, Bland JM, Cullum NA, Dumville JC, Iglesias CP, Kang'ombe AR, Soares MO, Stubbs NC, Torgerson DJ. Compression hosiery versus compression bandaging in the treatment of venous leg ulcers: a randomised controlled trial, mixed treatment comparison and decision analytic model. Health Technol Assess 2014;18:1-293, v-vi. doi: 10.3310/hta18570.
5. Ashby R, Gabe R, Ali S, Adderley U, Bland JB, Cullum N, Dumville J et al. Compression hosiery versus compression bandages in treatment of venous leg ulcers (Venous leg Ulcer Study IV, VenUS IV): a randomised controlled trial. *Lancet.* 2014;383:871-9.
6. Blecken SR, Villavicencio JL, Kao TC. Comparison of elastic versus nonelastic compression in bilateral venous ulcers: a randomized trial. J Vasc Surg. 2005;42:1150-5.
7. Iglesias C, Nelson EA, Cullum NA, Torgerson DJ; VenUS Team. VenUS I: a randomised controlled trial of two types of bandage for treating venous leg ulcers. Health Technol Assess. 2004 Jul;8(29):iii, 1-105.
8. Nelson EA, Iglesias CP, Cullum N, Torgerson DJ; VenUS I collaborators. Randomized clinical trial of four-layer and short-stretch compression bandages for venous leg ulcers (VenUS I). Br J Surg. 2004;91:1292-9.
9. O'Meara S, Tierney J, Cullum N, Bland JM, Franks PJ, Mole T, Scriven M. Four-layer bandage compared with short stretch bandage for venous leg ulcers: systematic review and meta-analysis of randomised controlled trials with data from individual patients. BMJ. 2009 Apr 17;338:b1344.
10. Lazareth I, Moffatt C, Dissemond J, Lesne Padieu AS, Truchetet F, Beissert S, Wicks G, Tilbe H, Sauvadet A, Bohbot S, Meaume S. Efficacy of two compression systems in the management of VLUs: results of a European RCT. J Wound Care. 2012;21:553-4, 556, 558
11. Moffatt CJ, Edwards L, Collier M, Treadwell T, Miller M, Shafer L, Sibbald G, Brassard A, McIntosh A, Reyzelman A, Price P, Kraus SM, Walters SA, Harding K. A randomised controlled 8-week crossover clinical evaluation of the 3M Coban 2 Layer Compression System versus Profore to evaluate the product performance in patients with venous leg ulcers. Int Wound J. 2008;5:267-79.
12. Szewczyk MT, Jawień A, Cierzniakowska K, Cwajda-Białasik J, Mościcka P. Comparison of the effectiveness of compression stockings and layer compression systems in venous ulceration treatment. Arch Med Sci. 2010;6:793-9.
13. Treweek S, Pitkethly M, Cook J, Fraser C, Mitchell E, Sullivan F, et al. Strategies to improve recruitment to randomised trials. Cochrane Database Syst Rev. 2018 doi:10.1002/14651858.

14. Hill B, Perri-Moore S, Kuang J, Bray BE, Ngo L, Doig A, et al. Automated pictographic illustration of discharge instructions with Glyph: impact on patient recall and satisfaction. Journal of the American Medical Informatics Association. 2016;23(6):1136-42.
15. McCrorie AD, Chen JJ, Weller R, McGlade KJ, Donnelly C. Trial of infographics in Northern Ireland (TINI): Preliminary evaluation and results of a randomized controlled trial comparing infographics with text. Cogent medicine. 2018;5(1):1483591.
16. Buljan I, Malički M, Wager E, Puljak L, Hren D, Kellie F, et al. No difference in knowledge obtained from infographic or plain language summary of a Cochrane systematic review: three randomized controlled trials. Journal of Clinical Epidemiology. 2018;97:86-94.
17. Kumar A, and Epley N. Undervaluing gratitude: expressers misunderstand the consequences of showing appreciation. Psychological Science 2018, 29(9), 1423 – 1435.
18. Edwards PJ, Roberts I, Clarke MJ, DiGuiseppi C, Wentz R, Kwan I, Cooper R, Felix LM, Pratap S. Methods to increase response to postal and electronic questionnaires. Cochrane Database of Systematic Reviews 2009, Issue 3. Art. No.: MR000008. DOI: 10.1002/14651858.MR000008.pub4.
19. Bland JM, Dumville JC, Ashby RL, Gabe R, Stubbs N, Adderley U, Kang'ombe AR, Cullum NA. Validation of the VEINES-QOL quality of life instrument in venous leg ulcers: repeatability and validity study embedded in a randomised clinical trial. BMC Cardiovasc Disord 2015;15:85
20. Curtis, L. & Burns, A. (2018) Unit Costs of Health and Social Care 2018, Personal Social Services Research Unit, University of Kent, Canterbury.
21. Srivastava, A., .Thomson SB. , Framework analysis: a qualitative methodology for applied policy research. Journal of Administration & Governance, 2009. 4(2): p. 72-79
22. Bernard, H. and G. Ryan, Analysisng qualitative data; systematic approaches. 2010,LA: Sage.
23. Ritchie, J. and L. Spencer, Qualitative data analysis for applied policy, in Analysing qualitative data, A. Bryman and R. Burgess, Editors. 1994, Routledge: Oxon. p. 173 - 194. https://doi.org/10.22024/UniKent/01.02.70995
24. O'Cathain A, Murphy E, Nicholl J (2010) Three techniques for integrating data in mixed methods studies. BMJ ; 341 doi: https://doi.org/10.1136/bmj.c4587.
25. NHS Health Research Authority. UK Policy Framework for Health and Social Care Research. 2018 https://www.hra.nhs.uk/planning-and-improving-research/policies-standards-legislation/uk-policy-framework-health-social-care-research/ (Accessed 3rd December 2019)
26. Medical Research Council. Good Research Practice: Principles and guidelines. https://mrc.ukri.org/publications/browse/good-research-practice-principles-and-guidelines/ (Accessed 3rd December 2019).
27. Bell, K., Clark, L., Fairhurst, C., Mitchell, N., Lenaghan, E., Blacklock, J., Cushnaghan, J., Cooper, C., Gittoes, N., O'Neill, T.W. and Shepstone, L., 2016. Enclosing a pen reduced time to response to questionnaire mailings. Journal of clinical epidemiology, 74, pp.144-150.
28. Mitchell, A.S., Cook, L., Dean, A., Fairhurst, C.M., Northgraves, M., Torgerson, D.J. and Reed, M., 2020. Using pens as an incentive for questionnaire return in an orthopaedic trial: an embedded randomised controlled retention trial. F1000research.
29. Sharp, L., Cochran, C., Cotton, S.C., Gray, N.M., Gallagher, M.E. and TOMBOLA group, 2006. Enclosing a pen with a postal questionnaire can significantly increase the response rate. Journal of clinical epidemiology, 59(7), pp.747-754.
30. White, E., Carney, P.A. and Kolar, A.S., 2005. Increasing response to mailed questionnaires by including a pencil/pen. American journal of epidemiology, 162(3), pp.261-266.

31. Gelfand JM., Hoffstad O., and Margolis DJ. 2002. Surrogate endpoints for the treatment of venous leg ulcers. J Invest Dermatol*,* 119**,** pp.1420-5.
