# Supplementary material for: Compression therapies for venous leg ulcers: The VENous Ulcer Study 6 (VenUS 6), an open, multicentre, randomised clinical trial
Source: PLoS Med. 2026 Jul 10;23(7):e1005154. doi: 10.1371/journal.pmed.1005154 (PMC13354008; doi:10.1371/journal.pmed.1005154)

| VenUS 6 |
|---|
| |
| **A randomised controlled trial of compression therapies for the treatment of venous leg ulcers (ISRCTN67321719)** |
| |
| Statistical Analysis Plan |

| | |
|---|---|
| **Version** | 1.0 |
| **Date** | 24-11-2023 |
| **Author(s)** | Charlie Welch, Catherine Hewitt, Luke Strachan |
| **Chief investigator** | Jo Dumville |
| **Trial manager** | Catherine Arundel |

Contents


1. Background and rationale ... 4
2. Trial objectives ... 4
3. Design ... 5
4. Randomisation ... 7
5. Sample size ... 7
6. Outcomes ... 7
   6.1. Primary outcome (blinded assessment of reference ulcer healing) ... 7
   6.2. Secondary outcomes ... 10
      6.2.1. Healing of reference ulcer (blind and unblind assessments) ... 10
      6.2.2. Healing of reference ulcer (nurse assessment) ... 10
      6.2.3. Healing of the reference leg (nurse assessment) ... 10
      6.2.4. Recurrence ... 10
      6.2.5. Ulcer related pain ... 10
      6.2.6. VEINES-QOL/Sym questionnaire ... 11
   6.3. Data Collection and Follow-up ... 11
      6.3.1. Screening/Consent data ... 11
      6.3.2. Baseline data (investigator completed) ... 12
      6.3.3. Baseline data (participant completed) ... 12
      6.3.4. One month follow up (participant completed) ... 13
      6.3.5. Three, six and twelve month follow up (participant completed) ... 13
      6.3.6. Clinical events (investigator completed) ... 13
      6.3.7. Nurse/Health Care Professional follow up visits ... 14
7. Data ... 14
   7.1. Case Report Forms ... 14
   7.2. Management of datasets and data verification ... 15
8. Analysis ... 15
   8.1. Screening Data ... 15
   8.2. Baseline Data ... 16
   8.3. Treatment Delivery ... 16
      8.3.1. Receipt of allocated treatment following randomisation ... 16
      8.3.2. Adherence to allocated treatment and ease of use ... 16
   8.4. Primary Outcome Analyses ... 17
      8.4.1. Preliminary analyses ... 17
      8.4.2. Primary analysis ... 17
      8.4.3. Competing risks analysis ... 23
      8.4.4. Subgroup analyses ... 24
   8.5. Analysis of Secondary Outcomes ... 24
      8.5.1. Healing of reference ulcer (blind and unblind assessments) ... 24
      8.5.2. Healing of reference ulcer (nurse assessment) ... 24
      8.5.3. Healing of reference leg (nurse assessment) ... 25
      8.5.4. Ulcer recurrence ... 25

8.5.5. Incidence of clinical events during trial treatment ........ 26
8.5.6. VEINES-QOL ........ 27
8.5.7. VEINES-Sym ........ 28
8.5.8. Ulcer related pain ........ 29
9. SAP Revisions ........ 29
10. Roles and responsibilities ........ 29
11. References ........ 30
Appendix A - Data processing rules and self-evident corrections ........ 32
Appendix B – Trial flow diagram ........ 49

# 1. Background and rationale

Venous leg ulcers are common, recurring open wounds on the lower leg. In the UK, venous leg ulcer care is mainly delivered in the community, often in patients' homes or clinics, by nurses or other health professionals. Previous influential randomised controlled trial (RCT) evidence shows that strong compression treatments (aiming to deliver around 40mmHg at the ankle) reduce ulcer healing time [1]. Strong compression is the first line treatment for venous leg ulcers, although the EVRA trial has recently shown that early endovenous ablation surgery, together with strong compression, results in faster healing [2]. Whilst early use of endovenous ablation is likely to increase over time, most people with a venous leg ulcer will continue to spend many weeks being treated in the community with compression whilst waiting for referral and then surgery. Furthermore, some people with ulcers cannot or will not have surgery. Maximising ulcer-free days and health related quality of life (subject to budgetary constraints) with optimal compression use therefore remains important to patients and the NHS.

The strong compression treatments being compared in this project are:

- Evidence based compression (four-layer bandage or two-layer compression hosiery)
- Two-layer bandage (excluding short stretch bandages)
- Compression wraps (adjustable hook-and-loop fastened compression systems)

Two-layer hosiery and compression wraps can be given to people for self-application in some cases, whereas bandages are usually applied by trained staff. Data from NIHR Health Technology Assessment (HTA) funded RCTs suggest that the four-layer bandage and two-layer hosiery confer similar healing times but two-layer hosiery is likely to be cost effective [3]. Current best practice compression is two-layer hosiery for eligible, willing and able people (i.e. those with non-oedematous legs and often more mobile and dexterous patients) and four-layer bandages for those unsuited to hosiery. We refer to this 'choice' approach as 'evidence-based compression'. There has been much less research on two-layer bandages: there are few data from trials and existing evidence is highly uncertain. There is almost no evidence available on the clinical- and cost-effectiveness of compression wraps. Despite the lack of supportive research evidence, national prescribing data suggest that the two-layer bandage is the most commonly-used compression treatment and that use of the four-layer bandage has declined accordingly. Two-layer hosiery use has increased but overall usage of hosiery is much lower than bandage use. Prescribing data suggest that compression wrap use is increasing but remains much lower than bandage or hosiery use.

# 2. Trial objectives

VenUS 6 is a three arm, parallel group, randomised controlled trial with internal pilot and process evaluation. The statistical analysis will address the following primary research objectives:

- To investigate whether two-layer bandaging is non-inferior to evidence based compression in terms of time to healing of venous leg ulcers

- To compare the effectiveness of compression wraps with evidence based compression and two-layer bandaging in terms of time to healing of venous leg ulcers

The statistical analysis will also compare evidence based compression, two-layer bandaging and compression wraps in terms of:

- time to ulcer recurrence
- venous leg ulcer symptoms and their impact on daily activities and functioning
- venous leg ulcer related pain
- complications and clinical events

# 3. Design

VenUS 6 is a three arm, multi-centre, pragmatic, parallel group, randomised controlled trial, featuring both non-inferiority and superiority comparisons. Eligible patients will be randomised (1:1:1) to one of the following treatments:

- **EBC** – Evidence based compression (four-layer bandage or two-layer compression hosiery)
- **2LB** – Two-layer bandage
- **CW** – Compression wraps (adjustable hook-and-loop fastened compression)

The study has a 32-month recruitment period, and a four month follow up period (following recruitment of the last patient) during which there will be no further recruitment. Follow-up will be variable with participants followed for minimum of four months (i.e. those recruited the very end of the recruitment period) and a maximum of 12 months. A flow diagram illustrating the patient/participant pathway through the study is provided in Figure 1.

Neither participants nor health care practitioners delivering treatment can be blinded to treatment allocation. To mitigate against possible ascertainment bias resulting from the lack of blinding, the primary outcome (time to healing) will be based on assessments made by independent, blinded observers using standardised photographs, with nurse reported time to healing being treated as a secondary outcome. Where a participant has multiple venous ulcers, the eligible ulcer with the largest surface area ($cm^2$) will be termed the reference ulcer. The primary outcome (time to healing) will be based on the healing of the reference ulcer. The leg on which the reference ulcer is located will be termed the reference leg. Other secondary outcomes that will be analysed as part of the statistical analysis are time to ulcer recurrence, venous leg ulcer symptoms and their impact on daily activities and functioning, venous leg ulcer related pain and complications/clinical events.

This is a pragmatic trial. Following randomisation, as well as being offered their allocated compression treatment, participants will receive standard care, including wound dressing changes, as per routine clinical practice. Data on treatment use and clinical outcomes will be collected during this period by

nurses. During the trial, participants will also be asked to complete outcome assessments (postal questionnaires) at 1, 3, 6, and 12 months post-randomisation.

**Figure 1:** Trial flow diagram

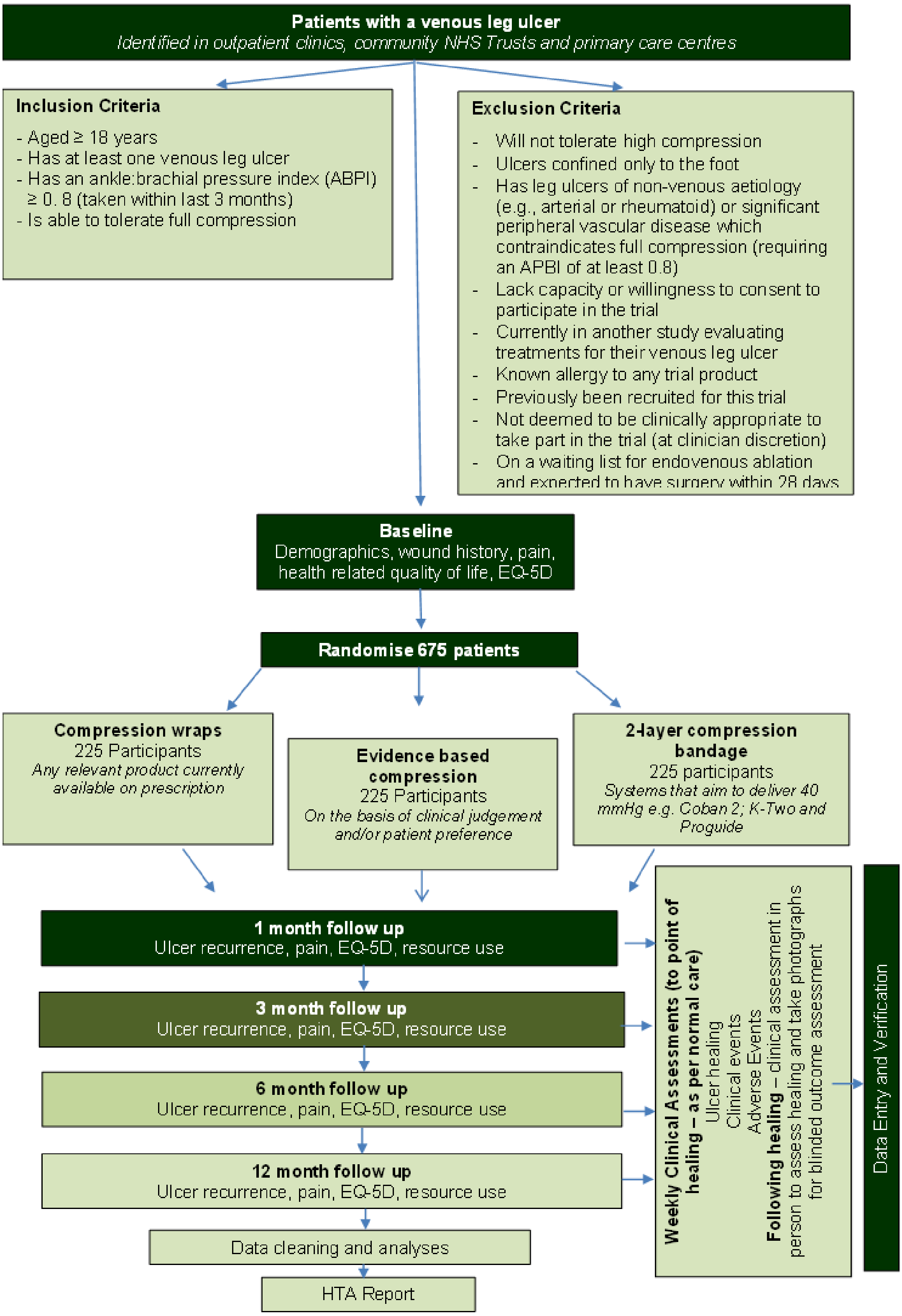

# 4. Randomisation

Following collection of baseline data, the research team will contact York Trials Unit (YTU) via the internet to access a secure randomisation service. The randomisation service will record key information (e.g. details used for stratifying the randomisation) and check eligibility to avoid inappropriate entry of patients into the trial. Block randomisation will be used with randomly varying block sizes (actual block sizes suppressed here to assist with maintaining allocation concealment), and will be stratified by two prognostic variables; reference ulcer duration at baseline (≤ 6 months and > 6 months) and reference ulcer area at baseline (≤ 5cm$^2$ and > 5cm$^2$). Once randomised, participants will begin their trial treatment as soon as it is available in line with what would happen in routine practice.

# 5. Sample size

The sample size calculation for this study is based on the findings of the HTA funded VenUS I [4] and VenUS IV [3] trials. A hazard ratio (HR) of 1.33 will be used as the non-inferiority margin for the comparison of 2LB with EBC. We assume a median time to healing of 2.3 months in the EBC group, an average follow-up time of 12 months and 10% attrition (pre-healing). We also assume that there is truly no difference between EBC and 2LB under the alternative hypothesis for this test (i.e. HR = 1). Under these assumptions, 225 patients per group are required to obtain 80% power for a one-sided test of size 2.5% of the null hypothesis that 2LB is inferior to EBC by a clinically relevant amount (i.e. HR = 1.33).

We also plan to recruit 225 patients to the compression wraps (CW) group. Under the same assumptions regarding healing rate in the EBC group, length of follow-up time and attrition as stated above, and assuming a hazard ratio of 1.33 (comparing CW with EBC) under the alternative hypothesis, this sample size (i.e. 225 per group) obtains 80% power for a superiority comparison of EBC and CW using a two-sided test of size 5%. If the 2LB and EBC groups are combined and compared 2:1 against CW, then under the same assumptions as the superiority comparison outlined above, this sample size (i.e. 450 vs 225) would obtain 90% power for a two-sided test of size 5%.

We will only combine the EBC and 2LB groups for the superiority comparison with CW if 2LB is found to be non-inferior to EBC (i.e. the null hypothesis of the non-inferiority comparison is rejected). If the null hypothesis of the non-inferiority comparison is not rejected, then the EBC and 2LB groups will not be combined, and CW will be compared with each of these groups individually (i.e. compared 1:1:1). Under the same alternative hypotheses (i.e. HR(EBC/2LB) = 1 and HR(CW/EBC) = 1.33) and assumptions (i.e. median healing time of 2.3 months in the EBC group, average follow-up time of 12 months, 10% attrition) as above, the power to detect superiority of CW over EBC, or EBC and 2LB, combined is approximately 86%.

# 6. Outcomes

## 6.1. Primary outcome (blinded assessment of reference ulcer healing)

The primary outcome for this trial is time to healing of the reference ulcer, defined as: complete epithelial cover in the absence of a scab with no dressing required. Treating nurses will be asked to report the date they consider the reference ulcer to have healed. A digital image will be taken at baseline and again when the treating nurse records the reference ulcer as healed. After this point, images will be taken once a week over the next 4 weeks. These images will be assessed by two clinical experts blind to trial allocation to confirm the date of healing, with disagreements being resolved through discussion and the involvement of a third reviewer if required. The blinded assessment of healing date will be used as the primary healing endpoint. The process used to derive healing times for the primary outcome is illustrated in Figure 2.

**Figure 2:** Primary outcome derivation process, where d is the event indicator (d = 1 if blinded outcome assessor confirmed healing and d = 0 otherwise) and t denotes the event time (healing or censoring). Note that competing events (death and/or amputation of the reference leg) will be treated as censoring events for the purposes of the primary analysis.

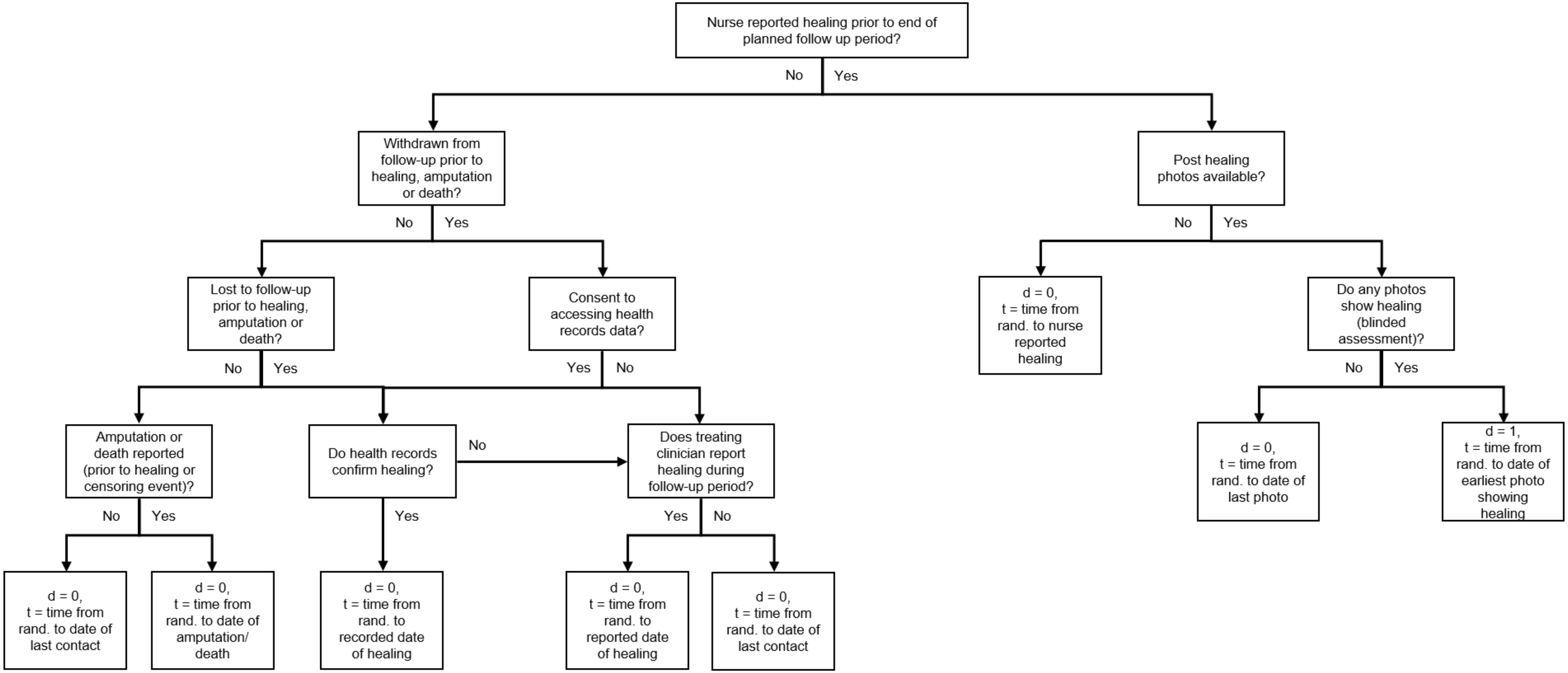

## 6.2. Secondary outcomes

### 6.2.1. Healing of reference ulcer (blind and unblind assessments)

For participants with available and sufficiently clear post-healing photography and participants that do not have nurse/clinician assessed healing reported this, outcome will be identical to the primary outcome. However, in contrast to the primary outcome, participants that have nurse/clinician healing reported, but do not have any post-healing photography of sufficient quality available will also be counted as healing events (with the date of healing based on the nurse/clinician reported date of healing).

### 6.2.2. Healing of reference ulcer (nurse assessment)

Treating nurses will be asked to report the date they consider the reference ulcer to have healed. Participants who withdraw from nurse follow-up (due to either full withdrawal or withdrawal from nurse follow-up specifically) prior to healing of the reference ulcer or death/amputation of the reference leg will be asked if the reference ulcer healing date can be collected from medical records once they reach the end of their planned follow-up period. Participants that consent to collection of this data will have healing times determined based on this information. Participants that do not consent to collection of this data, will have their healing times censored at the time of withdrawal. Healing times for participants that reach the end of their planned follow-up period without reference ulcer healing, death/amputation of the reference leg, or withdrawing, will have their healing times censored at the time of last contact. Healing times for participants who experience competing events (death, amputation of the reference leg), will be censored at the time the competing event occurred. The proportion of participants experiencing competing events is expected to be small.

### 6.2.3. Healing of the reference leg (nurse assessment)

Treating nurses will be asked to report the date they consider the reference leg to be healed. Healing times for participants who fully withdraw, are lost to follow up, or reach 12 months post-randomisation without the reference leg fully healing (or experiencing a competing event), will have their healing times censored at the time of last contact. Healing times for participants who experience competing events (death, amputation of the reference leg), will be censored at the time the competing event occurred. The proportion of participants experiencing competing events is expected to be small.

### 6.2.4. Recurrence

Following complete healing of the reference leg, monthly telephone assessments will be undertaken to assess ulcer recurrence until the participant exits the trial (either due to withdrawal or reaching 12 months post-randomisation). Recurrence times for participants who fully withdraw, are lost to follow up, or reach 12 months post-randomisation without recurrence (or experiencing a competing event), will be censored at the time of last contact. Recurrence times for participants who experience competing events (death, amputation of the reference leg), will be censored at the time the competing event occurred. The proportion of participants experiencing competing events is expected to be small.

### 6.2.5. Ulcer related pain

Participants will be asked to rate the intensity of any venous leg ulcer related pain that they have experienced during the previous 24 hours using a visual analogue scale. This instrument provides a score between 0 and 100 with higher scores indicating greater pain. Participants will also be asked to report the consistency of any venous leg ulcer related pain using a study specific five level ordinal scale (Painful all of the time, Painful most of the time, Painful some of the time, Painful when compression treatment is being changed, Not painful). These data will be collected from participants at baseline and 1, 3, 6 and 12 months post-randomisation.

### 6.2.6. VEINES-QOL/Sym questionnaire

Condition specific quality of life and symptoms will be assessed using the VEINES-QOL/Sym questionnaire, completed at baseline and 3, 6 and 12 months post-randomisation [5]. This questionnaire has 26 questions that are used to generate two summary scores; the VEINES Quality of Life (VEINES-QOL) score and the VEINES Symptom (VEINES-Sym) score. Both scores have a range of 0 to 100 with higher scores indicating a better outcome. The VEINES-QOL score provides a numerical measurement of the overall impact of venous leg ulceration on the participants' health related quality of life, and the VEINES-Sym score provides a numerical measurement of the severity/frequency of the symptoms participants' experience as a result of venous leg ulceration. The VEINES-QOL score is generated using the responses to all questions except question 2. The VEINES-Sym score is generated using the responses to the nine items which constitute question 1 and the response to question 7. Scores are generated as follows.

1. Question 3 (item 11), question 6 (item 20) and question 7 (item 21) are reverse scored
2. Question 4a (item 12) is set to missing if the response is 0 ("I do not work")
3. Each item is scored 1 to *k* where *k* is the number of categories for that item
4. Each item score *i* is scaled using *(i - 1)/(k - 1)* so that each item has a score between 0 and 1
5. The VEINES-QOL score is set to missing if >12 of the 25 items (including 4a) are missing
6. The VEINES-Sym score is set to missing if the response to question 7 (item 21) is missing, or if question 7 is not missing, but >4 of the nine items in question 1 are missing
7. The VEINES-QOL score is the arithmetic mean of the available scaled item scores (all items except question 2) multiplied by 100 (for participants whose score is not missing according to the conditions given in 5.)
8. The VEINES-Sym score is the arithmetic mean of the available scaled item scores (all nine items in question 1, and question 7) multiplied by 100 (for participants whose score is not missing according to the conditions given in 6.)

## 6.3. Data Collection and Follow-up

An overview of data collection and follow up is provided in Figure 1. Data will be collected by both investigators at study recruitment sites, and from participants themselves.

### 6.3.1. Screening/Consent data

- Inclusion criteria
  - Patient has confirmed venous leg ulcer and ulcer characteristics

  - Ankle : brachial pressure index ≥ 0.8 or other assessments to rule out arterial disease
  - Patient deemed suitable for strong compression
  - Patient aged over 18 years and date of birth
- Exclusion criteria
  - Ulcer confined only to foot
  - Other significant disease or disorder which warrants exclusion from the trial
  - Allergy to trial products
  - Patient is awaiting planned treatment to close/remove incompetent superficial veins (e.g. via endovenous ablation, sclerotherapy) and is expected to have surgery within 28 days
  - Patient unwilling to wear strong compression
  - Patient lacks capacity to provide full informed consent
  - Currently participating in another study evaluating treatments for venous leg ulcers
  - Patient previously recruited to trial
- Reason not approached for consent
- Consent
- Reasons for non-consent
- Treatment preference among patients who do not consent

#### 6.3.2. Baseline data (investigator completed)

- Presence and type of diabetes
- Previous surgeries for venous leg ulcers
- Number of previous episodes of ulceration of the reference leg
- Time since first episode of venous leg ulceration
- Duration of longest current episode of ulceration on the reference leg
- Duration of reference ulcer
- Position of all current venous leg ulcers
- Area of reference ulcer ($cm^2$)
- Weight and height
- Mobility status (Walks freely, Walks with difficulty, Immobile)
- Reference leg ankle mobility (Full range of motion, Reduced range of motion, ankle fixed)
- Reference leg ankle circumference (cm)
- Current treatment being received for reference ulcer
- Details of treatment application following randomisation

#### 6.3.3. Baseline data (participant completed)

- Gender
- Ethnicity
- Smoking status
- Employment status
- Intensity of ulcer related pain in previous 24 hours

- Consistency of ulcer related pain
- EQ-5D-5L
- VEINES -QOL/Sym Questionnaire
- Treatment preferences

#### 6.3.4. One month follow up (participant completed)

- Intensity of ulcer related pain in previous 24 hours
- Consistency of ulcer related pain
- EQ-5D-5L
- Self-reported assessment of allocated treatment
  - Satisfaction with treatment
  - Discomfort caused by treatment
  - Ease of use
  - Effect of treatment on ulcer related pain
  - Adherence to randomised treatment

#### 6.3.5. Three, six and twelve month follow up (participant completed)

- Intensity of ulcer related pain in previous 24 hours
- Consistency of ulcer related pain
- EQ-5D-5L
- VEINES-QOL/Sym Questionnaire
- Allocated treatment assessment
  - Satisfaction with treatment
  - Discomfort caused by treatment
  - Ease of use
  - Effect of treatment on ulcer related pain
  - Adherence to randomised treatment
- Resource use

#### 6.3.6. Clinical events (investigator completed)

- Date reference ulcer healed (nurse assessed)
- Date reference leg healed (nurse assessed)
- Ulcer recurrence following complete healing of reference leg (nurse assessed)
- Ulcer deterioration
- Skin deterioration
- Amputation of reference leg
- Hospital admissions related to reference ulcer
- Planned treatment of the reference leg to close/remove incompetent superficial veins
- Infection occurring on reference leg
- Occurrence of new ulcers on the reference leg
- Death

- Photographs of the reference ulcer each week for four weeks following nurse reported healing

#### 6.3.7. Nurse/Health Care Professional follow up visits

- Visit date
- Visit duration
- Visit location
- Clinical personnel delivering care
- Treatment activity
  - Reapplication of current compression treatment
  - Current compression treatment checked, but not replaced
  - Application of new compression treatment/treatment crossover
  - Compression treatment not applied
- Compression treatment items given
- Primary contact layer applied
- Reasons for changes to compression treatment

# 7. Data

## 7.1. Case Report Forms

Trial data will be captured primarily using paper Case Report Forms (CRFs), completed by study participants, treating nurses/health care professionals and trial site investigators. Copies of the CRFs marked up with variable names from the trial database are kept by the trial statistician in the statistical master file (see Y:\Project -- VENUS-6 - Statistics\2_Documentation\Case Report Forms\SPECs) and by both the trial management and data management teams. The CRFs that will be used for VenUS 6 are as follows;

- Screening for Eligibility (completed by trial investigators)
- Investigator Baseline
- Participant Baseline Questionnaire
- Participant 1 Month Questionnaire
- Participant 3 Month Questionnaire
- Participant 6 Month Questionnaire
- Participant 12 Month Questionnaire
- Participant Visit Log (completed by treating nurses)
- Participant Event Form (completed by trial investigators)
- Ulcer Healed Photography Form (completed by trial investigators)
- End of Follow-up Healing for Withdrawn Participants (completed by trial investigators)
- Blinded Outcome Assessment (completed by blinded outcome assessors)
- Adverse Event/Serious Adverse Event Form (completed by trial investigators)
- Adverse Event/Serious Adverse Event Form (completed by trial investigators)

- Participant Change of Status Form (completed by trial investigators)

### 7.2. Management of datasets and data verification

Comprehensive version controlled data validation plans have been devised for each CRF including checks for completeness, internal consistency, data formatting and range checks (see Y:\Project -- VENUS-6 - Statistics\2_Documentation\Case Report Forms\Validation plans). All data are validated according to these plans prior to export to the trial database. Current and previous versions of these validation plans are kept by the trial statistician in the statistical master file. The data management team will document any violations of the validation rules as well as any amendments following consultation with investigators at recruiting sites. Specified variables will not be queried with recruiting sites, due to them either being time sensitive (meaning data obtained at a later date would not be valid) or because of the burden such queries place on study participants. A range of self-evident corrections and processing rules are in place to address possible discrepancies that could occur among these data. These processing rules are detailed in Appendix A (note the marked up trial CRFs are required to follow these and can be made available on request).

The trial statistician will import data exports from the data management team into script based statistical software and will conduct further checks to investigate the consistency of data across different CRFs and check the range and format of any variables/quantities derived using the exported data (e.g. BMI derived using height and weight). Any anomalies identified during these processes will be documented and resolved in accordance with the procedures outlined in YTU SOP S02: Statistical Quality Control. Any changes to the dataset exported for the final analyses will be detailed in an assumptions log as described in YTU SOP S02: Statistical Quality Control.

## 8. Analysis

Analyses will be conducted once at the end of the trial using the latest available version of Stata/MP. For all analyses, the analysis sets will include all randomised participants with data available for the relevant outcome (unless otherwise stated), and participants will be analysed as part of the groups to which they were allocated. For time to event outcomes (healing times of reference ulcer and reference leg and time to recurrence), relative differences between groups will be presented in terms of hazard ratios (and appropriate two-tailed 95% confidence intervals), and absolute differences in terms of median healing times (and appropriate two-tailed 95% confidence intervals). For continuous (non-survival) outcomes (VEINES-QOL/Sym scores and intensity of ulcer related pain), differences between groups will be presented in terms of the differences in expected values (and appropriate two-tailed 95% confidence intervals). For binary outcomes (incidence of key clinical events), relative differences between groups will be presented in terms of the relative risk of the clinical event of interest (together with appropriate two-tailed 95% confidence intervals).

### 8.1. Screening Data

A summary of venous leg ulcer patients who did not end up being randomised will be provided. The following information will be reported

- Number of patients assessed for eligibility
- Number/proportion of patients assessed for eligibility who were found to be eligible
- Number/proportion of eligible patients who were approached for consent
- Number/proportion of patients approached for consent who consented to participation
- Number/proportion of consented patients who were randomised

Reasons for ineligibility and non-consent will be reported in both the trial CONSORT diagram and in tabular form (see Appendix B)

## 8.2. Baseline Data

Baseline data for all randomised participants (except any ineligible patients randomised in error) will be summarised descriptively by trial arm and overall. Continuous baseline data will be summarised in terms of the non-missing sample size, arithmetic mean, standard deviation, median, interquartile range, minimum and maximum. Categorical baseline data will be summarised in terms of frequencies and proportions. No formal between group comparisons of baseline data will be undertaken.

## 8.3. Treatment Delivery

Data relating to compression treatment delivery and concordance with the allocated treatment will be collected immediately following randomisation, during ongoing routine nurse follow up visits and in the participant completed follow up CRFs at 1, 3, 6 and 12 months post-randomisation. The reporting of these data will be primarily descriptive, although some formal comparisons will be undertaken to investigate differences in the proportion of treatment discontinuations in each arm, as well as differences in time to treatment discontinuation.

### 8.3.1. Receipt of allocated treatment following randomisation

The compression treatment received by participants immediately following randomisation (if any) will be reported by randomised group. Reasons for any departures from the allocated treatment will also be reported descriptively (where available). This information will also be reported in the trial CONSORT diagram (see Appendix B).

### 8.3.2. Adherence to allocated treatment and ease of use

Participant reported concordance with the allocated treatment at one month post randomisation will be reported, together with participants' experience of using their allocated treatment. The number and proportion of participants who begin using a different compression treatment from the one allocated prior to healing of the reference ulcer (or being censored) will be reported by randomised group, together with reasons for these departures. In some cases participants may not receive their allocated treatment following randomisation because this treatment is not available (e.g. compression wraps may need to be ordered following randomisation to the compression wraps group), and hence the participant may receive a different non-randomised treatment while they wait for the randomised treatment to arrive. These cases will be considered non-adherent if the participant continues to receive the non-randomised treatment for more than 14 days following randomisation. Cases that receive the allocated treatment within 14 days of randomisation will not be considered to have been non-adherent. The timing

of switches between treatments will be illustrated (by allocation) using the estimated cumulative incidence functions accounting for the competing risks of healing, death and amputation of the reference leg. The proportion of participants that switch treatment at some point prior to reference ulcer healing will be compared between groups using a mixed effect logistic regression model, with fixed effects for random allocation (2LB vs EBC vs CW), age, log reference ulcer area at baseline and log reference ulcer duration at baseline, and a random effect for recruitment site. The odds ratios for allocation (2LB vs EBC and CW vs EBC) will be reported with Wald method 95% CIs and p-values.

## 8.4. Primary Outcome Analyses

The primary outcome for this trial is time to healing of the reference ulcer, as defined in Section 6.1. The primary analysis will incorporate both non-inferiority and superiority comparisons. Due to the anticipated presence of intercurrent events that may impact treatment effectiveness, we will target two treatment effect estimands for the non-inferiority comparison (for reference, an estimand is a precise description of the treatment effect to be estimated with regards to five key attributes; (1) Population of interest, (2) Treatment strategies being compared, (3) Outcome of interest, (4) Treatment effect summary measure, (5) Handling of intercurrent events [6]). One estimand where all intercurrent events (e.g. treatment, switches, treatment discontinuation, receipt of surgery etc.) are accepted as part of completely pragmatic treatment policies, and a second estimand where certain constraints are placed on the intercurrent events that are included as part of the treatment policies being compared. For the superiority comparisons, just the unconstrained treatment policies will be compared. Precise specifications of the unconstrained and constrained treatment policies and the estimators/methods that will be used to estimate them are given in Section 8.4.2.

Estimates from the primary analyses will be supplemented with estimates from an analysis treating death and amputation of the reference leg as competing events (rather than censoring events). Additional analyses will be conducted to investigate the consistency of treatment effect estimates across potentially predictive baseline covariates (i.e. exploring baseline characteristics associated with treatment effect heterogeneity).

### 8.4.1. Preliminary analyses

Brief summaries of the total time at risk and number/proportion of participants who experienced the event of interest (namely healing of the reference ulcer) will be presented by randomised group and overall. The number and proportion of participants who are censored for different reasons (e.g. withdrawal, administrative censoring etc.), will be reported by group, as will the number and proportion of participants experiencing competing events (death or amputation of the reference leg). Time to healing (as defined in Section 6.1) will be illustrated using Kaplan-Meier cumulative incidence curves stratified by randomised group (without adjustment for any baseline covariates).

### 8.4.2. Primary analysis

As noted in section 8.4, for the non-inferiority comparison of EBC and 2LB both unconstrained (i.e. completely pragmatic) and constrained treatment policies will be compared. Precise description of the estimands targeted as part of the primary analysis are given in Table 1.

**Table 1**: Treatment effect estimands targeted for the primary analyses

| Attribute | Unconstrained estimand (Treatment policy estimand) | Constrained estimand (While of treatment estimand) |
|---|---|---|
| Population of interest | All randomised participants | Participants on treatment - i.e. receiving treatment in accordance with the allocated treatment policy |
| Treatment strategies being compared | Compression treatment of the designated reference leg with EBC, 2LB or CW, together with any treatment switches/ discontinuation (for any reason), and any receipt of additional non-compression ulcer related treatments (surgical or medical) | Compression treatment of the designated reference leg with EBC, 2LB or CW, excluding the following intercurrent events<br>• Not receiving allocated treatment following randomisation, and remaining on non-randomised treatment for >14 days (or until ulcer healing if this occurs before 14 days)<br>• Complete discontinuation of all compression treatment for a period of >7 days (for any reason)<br>• Receipt of surgical treatments to close/remove incompetent superficial veins or otherwise treat venous leg ulceration (reference leg only) |
| Outcome | Time to healing of the reference ulcer as defined in Section 6.1 | Time to healing of the reference ulcer as defined in Section 6.1 |
| Treatment effect summary | 1. Estimated hazard ratios for EBC vs 2LB, EBC vs CW and 2LB vs CW from Cox proportional hazards model with three level treatment group indicator (see details of model below)<br>2. Estimated hazard ratio for EBC+2LB vs CW from Cox proportional hazards model with two level treatment group indicator (see details of model below, contingent on results of non-inferiority comparison)<br>Proportional hazards (PH) will be assessed (see Section 8.4.2.3). If important departures from PH are identified, then a model with time varying treatment effects will be used to estimate hazard ratios over the whole 12 month follow-up period (see Section 8.4.2.3). The entire set of estimated hazard ratios over the 12 month follow-up period will be used for assessing non-inferiority | Estimated hazard ratios for EBC vs 2LB, EBC vs CW and 2LB vs CW from Cox proportional hazards model with three level treatment group indicator (see details of model below)<br>Proportional hazards (PH) will be assessed (see Section 8.4.2.3). If important departures from PH are identified, then a model with time varying treatment effects will be used to estimate hazard ratios over the whole 12 month follow-up period (see Section 8.4.2.3). The entire set of estimated hazard ratios over the 12 month follow-up period will be used for assessing non-inferiority |
| Handling of intercurrent events | Treatment policy (equivalent to ITT in this case) | While on treatment strategy (comparing the hazard of healing among participants that have not departed from the treatment strategies outlined above) |

#### 8.4.2.1 Non-inferiority comparisons

For the unconstrained treatment policy estimand, time to healing of the reference ulcer will initially be modelled using a Cox proportional hazards regression model with a three-level treatment group indicator (2LB vs EBC vs CW), conditioning on the following baseline covariates; reference ulcer area, reference ulcer duration, participant age, participant mobility status, and recruitment site (via shared

frailty for participants recruited at the same site). Details of these explanatory variables and how they will be included in the model are given in Table 2. Tied healing times will be handled using the Efron method [7]. The appropriateness of the proportional hazards assumption will be checked, with time varying treatment effects estimated and reported if important departures from proportional hazards is evident (see Section 8.4.2.3 for details of analyses undertaken in the presence of clear departures from proportional hazards).

**Table 2:** Terms included in the primary analysis outcome models

| Term | Interpretation | Type | Details |
|---|---|---|---|
| $z_{ij}$ | Randomised allocation for participant $i$ at recruitment site $j$ | Categorical | Three levels (2LB, EBC, CW) (two treatment group (2LB+EBC, CW) fitted if 2LB found to be non-inferior to EBC) |
| $\log(\text{area})_{ij}$ | Natural logarithm of the area of the reference ulcer (cm$^2$) at baseline for participant $i$ at recruitment site $j$ | Continuous | Restricted cubic spline with four knots placed at the following quantiles of the marginal distribution of the observed values of log(area): 5%, 35%, 65%, 95%. The generated spline basis variables will be centred around $\log(\text{area})_{ij} = \log(5)$<br><br>Any missing values will be imputed (prior to spline expansion) using the mean of the observed log-transformed values conditional on log(duration) and a random intercept for site (or just site if duration is also missing). These conditional means will be estimated via a linear mixed model |
| $\log(\text{duration})_{ij}$ | Natural logarithm of the duration of the reference ulcer (months) at baseline for participant $i$ at recruitment site $j$ | Continuous | Restricted cubic spline with four knots placed at the following quantiles of the marginal distribution of the observed values of log(duration): 5%, 35%, 65%, 95%. The generated spline basis variables will be centred around $\log(\text{duration})_{ij} = \log(6)$<br><br>Any missing values will be imputed (prior to spline expansion) using the mean of the observed log-transformed values conditional on log(area) and a random intercept for site (or just site if area is also missing). These conditional means will be estimated via a linear mixed model |
| $\text{age}_{ij}$ | Age at baseline for participant $i$ at recruitment site $j$ | Continuous | Restricted cubic spline with three knots placed at the following quantiles of the marginal distribution of the observed values of age: 10%, 50%, 90%. The generated spline basis variables will be centred around $\text{age}_{ij} = 60$<br><br>Any missing values of age will be imputed (prior to spline expansion) using the mean of the observed values conditional on a random intercept for site. These conditional means will be estimated via a linear mixed model |
| $\text{mobility}_{ij}$ | Mobility status at baseline for participant $i$ at recruitment site $j$ | Categorical | Three levels ("Walks freely", "Walks with difficulty", "Immobile"). Included in model via two indicator variables with "Walks freely" used as the reference category<br><br>Any missing values will be imputed with the most probable category conditional on log(area), log(duration), age and a random intercept for site. The relevant conditional probabilities will be estimated via mixed effect ordinal logistic regression |
| $u_j$ | Shared frailty for participants at recruitment site $j$ | Frailty | Centre level random effect (acting multiplicatively on the hazard function) from a gamma distribution with an expected value of 1 and variance estimated using the observed data |

For the constrained treatment policy, participants will have their healing times artificially censored at the first of any relevant departures from the constrained treatment policy (see Table 1). These healing times

will then be analysed using an identical outcome model as used to estimate the treatment effect for the unconstrained treatment policy, but time-varying (on a 28-day basis) stabilised inverse probability of censoring weights will be included in the estimation to mitigate against potential bias introduced by the aforementioned censoring. The weight determining models (one for each of the numerator and denominator of the time varying stabilised weights) will be fitted for each randomised group separately. These will be based on Cox proportional hazards regression with time to first departure from the constrained treatment policy as the outcome, conditioning on the baseline and time-varying covariates detailed in Table 3. Note, the time varying covariates will only be included in estimation of the denominator of the stabilised weights. The fitted models will be used to derive time varying stabilised weights for each 28-day time interval. The estimated weights will be checked, with appropriate action taken to address extreme weights if necessary (e.g. reducing the number of predictors in the weight-determining model, truncating extreme values of continuous predictors etc.).

**Table 3:** Terms included in the primary analysis weight-determining models

| Term | Interpretation | Type | Details |
|---|---|---|---|
| $\log(\text{area})_{ij}$ | Natural logarithm of the area of the reference ulcer ($cm^2$) at baseline for participant $i$ at recruitment site $j$ | Continuous | Linear term<br><br>Any missing values will be imputed using the mean of the observed values conditional on log(duration) and a random intercept for site (or just site if duration is also missing). These conditional means will be estimated via a linear mixed model<br><br>Included in estimation of weights for both the numerator and denominator of the stabilised weights |
| $\log(\text{duration})_{ij}$ | Natural logarithm of the duration of the reference ulcer (months) at baseline for participant $i$ at recruitment site $j$ | Continuous | Linear term<br><br>Any missing values will be using the mean of the observed values conditional on log(area) and a random intercept for site (or just site if area is also missing). These conditional means will be estimated via a linear mixed model<br><br>Included in estimation of weights for both the numerator and denominator of the stabilised weights |
| $\text{age}_{ij}$ | Age at baseline for participant $i$ at recruitment site $j$ | Continuous | Linear term<br><br>Any missing values of age will be imputed using the mean of the observed values conditional on a random intercept for site. These conditional means will be estimated via a linear mixed model<br><br>Included in estimation of weights for both the numerator and denominator of the stabilised weights |
| $u_j$ | Shared frailty for participants at recruitment site $j$ | Frailty | Centre level random effect (acting multiplicatively on the hazard function) from a gamma distribution with an expected value of 1 and variance estimated using the observed data<br><br>Included in estimation of weights for both the numerator and denominator of the stabilised weights |
| $\text{episodes\_det}_{ijt}$ | Cumulative number of episodes of skin/ulcer deterioration on the reference leg | Non-negative count | Truncated at the 95th percentile of the observed values and modelled using a linear term<br><br>Included in estimation of weights for just the denominator of the stabilised weights |

| | | | |
|---|---|---|---|
| | for participant $i$ at recruitment site $j$ at the start of time period $t$ | | |
| $\text{episodes\_trauma}_{ijt}$ | Cumulative number of episodes of skin trauma on the reference leg for participant $i$ at recruitment site $j$ at the start of time period $t$ | Non-negative count | Truncated at the 95th percentile of the observed values and modelled using a linear term<br><br>Included in estimation of weights for just the denominator of the stabilised weights |
| $\text{episodes\_infection}_{ijt}$ | Cumulative number of episodes of infection of the reference leg for participant $i$ at recruitment site $j$ at the start of time period $t$ | Non-negative count | Truncated at the 95th percentile of the observed values and modelled using a linear term<br><br>Included in estimation of weights for just the denominator of the stabilised weights |
| $\text{admissions}_{ijt}$ | Cumulative number of hospital admissions related to the reference leg for participant $i$ at recruitment site $j$ at the start of time period $t$ | Non-negative count | Linear term<br><br>Included in estimation of weights for just the denominator of the stabilised weights |

For both estimands, the point estimates of the HRs for all between group contrasts will be reported (see Section 8.4.2.3 for details of the reporting of time-varying HRs if important departures from proportional hazards are found). For the unconstrained treatment policy, Wald method 95% confidence intervals will be reported (calculated using model-based SEs if time invariant treatment effects are estimated, and delta method SEs if time varying treatment effects are estimated). For the constrained treatment policy, the 95% confidence intervals will be obtained via non-parametric bootstrapping of the entire inverse-probability of censoring weighted analysis (bias corrected with resampling stratified by treatment group).

If single HRs comparing EBC and 2LB are appropriate (i.e. the proportional hazards assumption is apparently reasonable), the estimated upper 95% confidence limits of the HRs for the EBC vs 2LB contrast for each estimand (Treatment policy and While-on-treatment) will be compared with the non-inferiority margin of 1.33. If either of the estimates is greater than 1.33, then the null hypothesis that 2LB is inferior to EBC will not be rejected. If both estimates are less than 1.33, then the null hypothesis that 2LB is inferior to EBC will be rejected. Should time-varying treatment effects be indicated (i.e. the proportional hazards assumption is inappropriate), non-inferiority will be assessed in a similar manner, but will instead be based on the supremums of the sets of the estimated upper confidence limits for the treatment effects observed over the 12 months of follow-up. A schematic of the potential outcomes of the non-inferiority comparison is provided in Table 4. In addition to the estimated HRs, we will also use the fitted models to estimate and plot the marginal (sample averaged) survival functions by allocation

and obtain estimates of differences in median healing time and cumulative incidence of healing at 1, 3, 6 and 12 months, together with appropriate 95% confidence intervals.

**Table 4:** Potential outcomes of the non-inferiority analyses comparing 2LB and EBC (assuming a single summary of treatment effect is appropriate). Here $u_1$ denotes the least upper bound (supremum) of the set of upper 95% confidence limits of the HR(s) comparing EBC and 2LB for the unconstrained treatment policies. $u_2$ denotes the least upper bound (supremum) of the set of upper 95% confidence limits for the constrained treatment policies

| | $u_1$ < 1.33 | $u_1$ ≥ 1.33 |
|---|---|---|
| $u_2$ < 1.33 | **Inferiority rejected** (2LB non-inferior to EBC) | **Inferiority not rejected** (2LB inferior to EBC) |
| $u_2$ ≥ 1.33 | **Inferiority not rejected** (2LB inferior to EBC) | **Inferiority not rejected** (2LB inferior to EBC) |

#### 8.4.2.2 Superiority comparisons

The point estimates and 95% confidence intervals of the HRs for the EBC vs CW and 2LB vs CW contrasts from the three-treatment group model (fitted to estimate the unconstrained treatment policy estimand for the non-inferiority comparisons) will be reported. In addition to the estimated HRs, we will also use the fitted models to estimate and plot the marginal (sample averaged) survival functions by allocation and obtain estimates of differences in median healing time and cumulative incidence of healing at 1, 3, 6 and 12 months, together with appropriate 95% confidence intervals. If the null hypothesis that 2LB is inferior to EBC is rejected (see Section 8.4.2.1) then a further model will be fitted with the 2LB and EBC groups combined, with the same fixed effect covariates and shared frailties as previously (see Table 2). The point estimate and 95% confidence interval of the HR for 2LB+EBC vs CW contrast will be reported. In addition to the estimated HRs, we will also use the fitted models to estimate and plot the marginal (sample averaged) survival functions by allocation and obtain estimates of differences in median healing time and cumulative incidence of healing at 1, 3, 6 and 12 months, together with appropriate 95% confidence intervals. As for the non-inferiority analyses, if there are important departures from the proportional then a model allowing for time-varying treatment effects will be fitted (see Section 8.4.2.3 for details).

#### 8.4.2.3 Proportional hazards assumption

Both the sample size calculation and the planned primary analyses assume the treatment effects meet the proportional hazards assumption. If this assumption is violated, then tests of between group differences based on the fitted model(s) may lose statistical power. In addition, a single summary of the differences between treatments will no longer be appropriate. The model(s) fitted for the primary

analysis also assume proportional hazards for the other prognostic covariates included in the model. The proportional hazards assumption for both the treatment effects and the covariates will be assessed using a range of diagnostic plots and statistical tests.

The extent to which the terms modelling the effects of the baseline covariates meet the proportional hazards assumption will be assessed first. This assessment will be based on plots of the scaled Schoenfeld residuals from the primary analysis model(s) against (untransformed) analysis time, and associated Grambsch-Therneau tests [8, 9]. If these plots and/or tests suggest the effects of particular baseline covariates seriously violate the proportional hazards assumption, then a model allowing for time varying effects of these covariates will be fitted via inclusion of interactions between the relevant baseline covariates and a flexible function of analysis time in the linear predictor. This will be accomplished using the flexible parametric modelling approach proposed by Royston and Parmar [10] in order to facilitate subsequent estimation of marginal differences in survival functions (e.g. differences in median healing times and cumulative incidence of healing). The estimated treatment effect(s) from this model will be reported in a similar manner to the primary analysis.

The extent to which the effect(s) of treatment meet the proportional hazards assumption will then be investigated using a plot of $-\log[-\log(\hat{S}(t))]$ against $\log(t)$ stratified by randomised group, where $t$ denotes analysis time and $\hat{S}(t)$ denotes the estimated survival at time $t$ from the relevant fitted Cox model with continuous covariates set to their median values and mobility status set to its modal value. Further investigation will be based on plots of the scaled Schoenfeld residuals against analysis time, and associated Grambsch-Therneau tests. If any of these plots and/or tests suggest the effects of treatment seriously violate the proportional hazards assumption, then a model with time varying treatment effects will be fitted via inclusion of interactions between treatment group and a flexible function of analysis time. This will be accomplished using the flexible parametric modelling approach proposed by Royston and Parmar [10] in order to facilitate subsequent estimation of marginal differences in survival functions (e.g. differences in median healing times and cumulative incidence of healing). Models with time varying treatment effects will be used to derive and plot hazard ratios for allocation over the whole 12 month follow-up period, together with appropriate 95% confidence intervals. The estimated marginal (sample averaged) survival functions will be plotted by randomised group, together with estimated differences in survival between groups (with appropriate 95% confidence intervals).

### 8.4.3. Competing risks analysis

The proportion of participants who experience competing events (amputation of the reference leg or death) will be summarised by randomised group and overall. Non-parametric estimates of the cumulative incidence functions for ulcer healing (the event of primary interest) and a composite of amputation and death (the competing event), will be plotted by randomised group.

If greater than or equal to 10% of randomised patients experience a competing event, then a semi-parametric estimate of the cumulative incidence function for ulcer healing in the presence of the

competing events, will be obtained for each randomised group, using cause specific proportional hazards regression models for each type of event. The cause specific hazards model for ulcer healing will condition on the same covariates as the primary analysis (see Table 1). The cause specific hazards model for the competing event will condition on linear terms for age, ulcer area (log transformed) and ulcer duration (log transformed). These models will be used to derive estimates of the hazard of each type of event at each event time and the probability of being event free at each event time. These estimates will be used to estimate the cumulative incidence function for ulcer healing for each randomised group, with baseline covariates set to representative values (median value for continuous covariates and modal value for categorical covariates). The estimated cumulative incidence functions will be used to estimate the difference between groups in median time to healing (accounting for competing events), together with bias corrected 95% confidence intervals based on a non-parametric bootstrap (2500 replicates).

### 8.4.4. Subgroup analyses

We will conduct exploratory analyses to assess whether reference ulcer size and duration are together a possible source of treatment effect heterogeneity. This analysis will be undertaken regardless of the results of the primary analysis. If 2LB is found to be non-inferior to EBC (i.e. the null hypothesis of inferiority is rejected) then the subgroup investigations will be conducted using both the three treatment group model, and the two treatment group model (i.e. with EBC and 2LB combined). If 2LB is found to be inferior (i.e. the null hypothesis of inferiority is not rejected) then only the three treatment group model will be used for subgroup investigations. In either case, a similar model(s) to the primary analysis will be fitted, but with a term for Margolis Index score at baseline (see Table 3 below) and all two-way interactions between treatment group and Margolis Index score added to the linear predictor [11]. The point estimates of the hazard ratios for allocation within each Margolis Index subgroup will be presented together with Wald method 95% confidence intervals. The reference ulcer Margolis Index score for participant $i$ at recruitment site $j$ at baseline is defined as follows;

**Table 3:** Margolis Index Score definition

| Margolis Index Score | Definition |
|---|---|
| 0 | Reference ulcer area ≤ 5cm$^2$ & Reference ulcer duration ≤ 6 months |
| 2 | Reference ulcer area > 5cm$^2$ & Reference ulcer duration > 6 months |
| 1 | Otherwise |

## 8.5. Analysis of Secondary Outcomes

### 8.5.1. Healing of reference ulcer (blind and unblind assessments)

This outcome is identical to the primary outcome (see Section 6.1) for participants with available post-healing photography, but also makes use of healing times calculated based on the healing dates reported by the treating nurses/clinicians for participants that do not have any post-healing photography. This outcome will be analysed in an identical manner to the primary outcome (see Section 8.4.1 and 8.4.2).

### 8.5.2. Healing of reference ulcer (nurse assessment)

This outcome is defined similarly to the primary outcome (see Section 6.1), but with healing times calculated based on the healing dates reported by the treating nurses (as opposed to the healing dates reported by blinded outcome assessors using photographs). This outcome will be analysed in an identical manner to the primary outcome (see Section 8.4.1 and 8.4.2).

### 8.5.3. Healing of reference leg (nurse assessment)

This outcome is defined in Section 6.2.3. Summaries of the time at risk and number/proportion of participants who experienced the event of interest (namely nurse reported complete healing of the reference leg) will be presented by randomised group and overall. The number/proportion of participants who are lost to follow up, or reach the end of follow up without experiencing an event, will be reported by group, as will the number/proportion of participants experiencing competing events (death or amputation of the reference leg).

Time to healing of the reference leg will be modelled using a Cox proportional hazards regression model with a three level treatment group indicator (EBC vs 2LB vs CW), adjusting for reference ulcer area at baseline (log-transformed and modelled using a restricted cubic spline with three knots at the $10^{th}$, $50^{th}$ and $90^{th}$ percentiles of the observed data), duration of ulceration of the reference leg at baseline (log-transformed and modelled using a restricted cubic spline with four knots at the $5^{th}$, $35^{th}$, $65^{th}$ and $95^{th}$ percentiles of the observed data), age at baseline (modelled using a restricted cubic spline with three knots at the $10^{th}$, $50^{th}$ and $90^{th}$ percentiles of the observed data), participant mobility status, and recruitment site (via shared frailties for participants recruited at the same site). Tied healing times will be handled using the Efron method [7]. Any missing values of reference ulcer area and age will be imputed as for the primary analysis. Any missing values of duration of ulceration of the reference leg at baseline will be imputed with the duration of the reference ulcer at baseline. If duration of the reference ulcer is also missing, then duration of ulceration of the reference leg at baseline will be imputed with the mean of the observed values. The point estimates and Wald method 95% confidence intervals of the HRs for all between group contrasts will be reported. In addition to the estimated HRs, we will also use the fitted model to estimate and plot the marginal (sample averaged) survival functions by allocation and obtain estimates of differences in median healing time and cumulative incidence of healing at 1, 3, 6 and 12 months, together with appropriate 95% confidence intervals.

The analysis specified for this outcome assumes proportional hazards and violation of this assumption may compromise power and cast doubt on the validity of a single numerical summary of treatment effectiveness over the whole follow up period. The extent to which the effect of treatment meets the proportional hazards assumption will be assessed and potentially addressed following the same approach specified for the primary analysis model (see Section 8.4.2.3).

### 8.5.4. Ulcer recurrence

This outcome is defined in Section 6.2.4. The number of participants who experience complete healing of the reference leg will be reported by randomised group, and these participants will be included in the analysis comparing rates of ulcer recurrence.

Summaries of the time at risk of recurrence and number/proportion of participants who experienced the event of interest (namely ulcer recurrence on the previously healed reference leg) will be presented by randomised group and overall. The number/proportion of participants who are subsequently lost to follow up, or reach the end of follow up without experiencing an event, will be reported by group, as will the number/proportion of participants experiencing competing events (death or amputation of the reference leg).

Time to recurrence will be modelled using a Cox proportional hazards regression model with a three level treatment group indicator (EBC vs 2LB vs CW), adjusting for reference ulcer area at baseline (log-transformed and modelled using a restricted cubic spline with three knots at the 10$^{th}$, 50$^{th}$ and 90$^{th}$ percentiles of the observed data), reference ulcer duration at baseline (log-transformed and modelled using a restricted cubic spline with three knots at the 10$^{th}$, 50$^{th}$ and 90$^{th}$ percentiles of the observed data), age at baseline (modelled using a restricted cubic spline with three knots at the 10$^{th}$, 50$^{th}$ and 90$^{th}$ percentiles of the observed data), participant mobility status, ulcer chronicity at baseline (log-transformed and modelled using a linear term) and recruitment site (via shared frailties for participants recruited at the same site). Tied healing times will be handled using the Efron method [7]. Any missing values of reference ulcer area, reference ulcer duration, age and mobility will be imputed as for the primary analysis. Any missing values of (log-transformed) ulcer chronicity will be imputed with the mean of the observed log-transformed values. The point estimates and Wald method 95% confidence intervals of the HRs for all between group contrasts will be reported. In addition to the estimated HRs, we will also use the fitted model to estimate and plot the marginal (sample averaged) survival functions by allocation and obtain estimates of differences in median recurrence time together with appropriate 95% confidence intervals.

The analysis specified for this outcome assumes proportional hazards and violation of this assumption may compromise power and cast doubt on the validity of a single summary measure of treatment effectiveness over the whole follow up period. The extent to which the effect of treatment meets the proportional hazards assumption will be assessed and addressed following a similar approach specified for the primary analysis model (see Section 8.4.2.3).

### 8.5.5. Incidence of clinical events during trial treatment

In addition to the reference ulcer and reference leg healing dates, information about four other clinical events relating to the reference leg will be collected using the participant clinical event form. These events are; ulcer related infection, ulcer deterioration, skin deterioration and occurrence of a new ulcer(s). For each of these four events, the difference in incidence between groups will be estimated following the approach detailed in [12] and outlined below;

At time $t = 0$ no patient has experienced either the clinical event of interest (e.g. ulcer related infection on the reference leg) or a competing event. For these analyses, a competing event is defined as any one of the following events occurring prior to complete healing of the reference leg

- Death from any cause
- Amputation of the reference leg
- Permanent discontinuation of allocated treatment followed by 7 days without event of interest

Let $T_i$ denote the (earliest) time at which participant $i$ moves from state 0 (event free) to either state 1 (event of interest has occurred) or state 2 (competing event has occurred). Let $\epsilon_i$ denote the event type ($\epsilon_i = 1$ if event of interest, $\epsilon_i = 2$ if competing event) and $C_i$ denote the (potential) censoring time for participant $i$. For each participant only the minimum of the event time and the censoring time is observed, hence each participant provides an observation of the form $[\min\{T_i, C_i\}, \mathbb{I}(T_i \leq C_i)\epsilon_i]$, where $\mathbb{I}(\cdot)$ denotes the standard 0-1 indicator function. Finally let $\tau_{\max}$ denote the minimum of the maximum follow-up/censoring times in each of the three randomised groups, and $\tau_{p50}$ denote the minimum of the 50th percentiles of the observed follow-up/censoring times across the three randomised groups.

We will use the (non-parametric) Aalen-Johansen estimator of the cause specific cumulative incidence function to time $\tau$ (=$\tau_{\max}$ or $\tau_{p50}$) to estimate $\widehat{p_Z}(\tau)$ (i.e. the incidence of the event of interest in randomised group $Z$ by time $\tau$) [13]. These estimates will be obtained using the community contributed Stata command `stcompet` [14]. The variance estimates $\widehat{s_Z}(\tau)^2$ will obtained using a non-parametric bootstrap (2500 replicates). The point estimates $\widehat{p_Z}(\tau)$ will be used to derive risk ratios for each between group comparison (2LB vs EBC, CW vs EBC and 2LB vs CW) at time $\tau$, denoted $\widehat{RR}(\tau)$. The variance estimate for the relative risk comparing groups $Z_j$ and $Z_k$ will then be computed using the following estimator

$$\widehat{\text{var}}\left(\log\left(\widehat{RR_{jk}}(\tau)\right)\right) = \frac{\widehat{s_{Z_j}}(\tau)^2}{\widehat{p_{Z_j}}(\tau)^2} + \frac{\widehat{s_{Z_k}}(\tau)^2}{\widehat{p_{Z_k}}(\tau)^2}$$

For each event (ulcer related infection, ulcer deterioration, skin deterioration and occurrence of a new ulcer(s)), the point estimates of the three relative risks (2LB vs EBC, CW vs EBC, 2LB vs CW), will be reported together with Wald method 95% confidence intervals obtained using the variance estimator given above.

### 8.5.6. VEINES-QOL

Analysis of the VEINES-QOL will include all participants with at least one observed post-randomisation VEINES-QOL score. The between group differences (2LB – EBC and CW – EBC) in mean VEINES-QOL score at each post-randomisation time point, will be estimated using a covariance pattern model, with all post-randomisation VEINES-QOL scores included as outcomes. Treatment group (EBC, 2LB or CW) and time point (month 3, 6 or 12) and their interaction will be included as fixed effects. The model will also adjust for various informative baseline covariates as fixed effects, namely baseline VEINES-QOL score (modelled using a linear term), ulcer area at baseline (log-transformed and modelled using a restricted cubic spline with three knots at the 10th, 50th and 90th percentiles of the observed data), ulcer duration at baseline (log-transformed and modelled using a restricted cubic spline with three knots at the 10th, 50th and 90th percentiles of the observed data), participant age (modelled using a restricted

cubic spline with three knots at the 10th, 50th and 90th percentiles of the observed data) and participant mobility status. A random intercept will be included to account for correlation among participants recruited at the same site, and correlation between repeated measurements (within participants) will be accounted for using an unstructured covariance matrix. This model will be fitted using restricted maximum likelihood estimation, with denominator degrees of freedom being calculated using the method of Kenward and Roger [15]. This model will be used to derive estimates of the between group differences in mean VEINES-QOL score at each post-randomisation time point, together with two-sided Wald method 95% CIs and p-values. Key model assumptions (conditional normality, homoscedasticity of lowest level residuals) will be checked. If these appear to be severely violated then a mixed effect semi-parametric proportional odds model will be fitted [16] to each time point separately, with the same fixed and random effect specification as outlined above (minus the effects of time point). This model will be used to derive estimates of the between group differences in mean scores and/or differences for any other quantiles of interest, together with two-sided 95% confidence intervals and p-values based on delta method standard errors.

### 8.5.7. VEINES-Sym

Analysis of the VEINES-Sym will include all participants with at least one observed post-randomisation VEINES-Sym score. The between group differences (2LB – EBC and CW – EBC) in mean VEINES-QoL score at each post-randomisation time point, will be estimated using a covariance pattern model, with all post-randomisation VEINES-Sym scores included as outcomes. Treatment group (EBC, 2LB or CW) and time point (month 3, 6 or 12) and their interaction will be included as fixed effects. The model will also adjust for various informative baseline covariates as fixed effects, namely baseline VEINES-Sym score (modelled using a linear term), ulcer area at baseline (log-transformed and modelled using a restricted cubic spline with three knots at the 10th, 50th and 90th percentiles of the observed data), ulcer duration at baseline (log-transformed and modelled using a restricted cubic spline with three knots at the 10th, 50th and 90th percentiles of the observed data), participant age (modelled using a restricted cubic spline with three knots at the 10th, 50th and 90th percentiles of the observed data) and participant mobility status. A random intercept will be included to account for correlation among participants recruited at the same site, and correlation between repeated measurements (within participants) will be accounted for using an unstructured covariance matrix. This model will be fitted using restricted maximum likelihood estimation, with the number of degrees of freedom being adjusted using the method of Kenward and Roger [15]. This model will be used to derive estimates of the between group differences in mean VEINES-Sym score at each post-randomisation time point, together with two-sided Wald method 95% CIs and p-values. Key model assumptions (conditional normality, homoscedasticity of lowest level residuals) will be checked. If these appear to be severely violated then a mixed effect semi-parametric proportional odds model will be fitted [16] to each time point separately (minus the effects of time point), with the same fixed and random effect specification as outlined above. This model will be used to derive estimates of the between group differences in mean scores and/or differences for any other quantiles of interest, together with two-sided 95% confidence intervals and p-values based on delta method standard errors.

### 8.5.8. Ulcer related pain

Intensity of ulcer related pain at each time point (1, 3, 6 and 12 months post randomisation) will be reported descriptively (non-missing sample size, mean, SD, median, inter-quartile range, range) and graphically (histograms and box plots). These will be reported in two ways. One set of summaries will include all participants with available data (at each time point), including those participants who indicate that they have no ulcers, assigning these participants a score of 0 for the relevant follow up. The other set will include (at each follow up) only those participants who are still suffering from at least one venous leg ulcer. Patient reported consistency of pain at each time point will also be reported descriptively. The frequency and proportion of participants in each of the following categories will be reported by randomised group at each time point; No leg ulcer(s), Leg ulcer(s) not painful, Leg ulcer(s) painful only when compression treatment removed, Leg ulcer(s) painful some of the time, Leg ulcers painful most of the time, Leg ulcer painful all of the time, Other.

## 9. SAP Revisions

| Amendment/addition to SAP and reason for change | New version number, name and date |
|---|---|
| | |
| | |
| | |

## 10. Roles and responsibilities

| Name | Trial Role | Signature | Date |
|---|---|---|---|
| Prof. Jo Dumville | Chief Investigator | | 5th Feb 2024 |
| Ms Catherine Arundel | Trial Manager | | 05.02.2024 |
| Mr. Charlie Welch | Trial Statistician | | |
| Ms Susan Dutton | Chair of DMC | Sign-off via email (Y:\Project -- VENUS-6 - Statistics\6_SAP\University of York Mail - Re. VenUS 6 SAP Review_Sign-off.pdf) | 10-10-2023 |
| Prof. Georgina Gethin | Chair of TSC | | 13-12-2023 |

# 11. References


[1] O'Meara S., Cullum N., Nelson E.A., Dumville J.C., *Compression for venous leg ulcers*, Cochrane Database of Systematic Reviews, 2012 11; Article Number: CD000265

[2] Gohel M.S., Heatley F., Liu X., Bradbury A., Bulbulia R., Cullum N., Epstein D.M., Nyamekye I., Poskitt K.R., Renton S., Warwick J., Davies A.H., et al., *A Randomized Trial of Early Endovenous Ablation in Venous Ulceration* New England Journal of Medicine, 2018 378(22); 2105-2114

[3] Ashby R., Gabe R., Ali S., Adderley U., Bland J.B., Cullum N., Dumville J. et al., *Compression hosiery versus compression bandages in treatment of venous leg ulcers (Venous leg Ulcer Study IV, VenUS IV): a randomised controlled trial*, Lancet, 2014 383; 871-879

[4] Nelson E.A., Iglesias C.P., Cullum N., Torgerson D.J., et al., *Randomized clinical trial of four-layer and short-stretch compression bandages for venous leg ulcers (VenUS I)*, British Journal of Surgery, 2004 91; 1292-1299.

[5] Bland J.M., Dumville J.C., Ashby R.L., Gabe R., Stubbs N., Adderley U., Kang'ombe A.R., Cullum N.A., *Validation of the VEINES-QOL quality of life instrument in venous leg ulcers: repeatability and validity study embedded in a randomised clinical trial*, BMC Cardiovascular Disorders, 2015 15; 85

[6] ICH E9 statistical principles for clinical trials. *European Medicines Agency*. Published September 17, 2018. https://www.ema.europa.eu/en/ich-e9-statistical-principles-clinical-trials

[7] Efron B., *The efficiency of Cox's likelihood function for censored data*, Journal of the American Statistical Association, 1977 72; 557-565

[8] Schoenfeld D., *Chi-squared goodness of fit tests for the proportional hazards regression model*, Biometrika, 1980 67(1); 145-153

[9] Grambsch P.M., Therneau T.M., *Proportional hazards tests and diagnostics based on weighted residuals*, Biometrika, 1994 81(3); 515-526

[10] Royston, P., and M. K. B. Parmar. 2002. Flexible parametric proportional-hazards and proportional-odds models for censored survival data, with application to prognostic modelling and estimation of treatment effects. Statistics in Medicine 21: 2175–2197.

[11] Margolis D.J., Allen-Taylor L., Hoffstad O., Berlin J., *The accuracy of venous leg ulcer prognostic models in a wound care system*, Wound Repair and Regeneration, 2004 12; 163-168

[12] Stegherr R., Schmoor C., Lübbert M., Friede T., Beyersmann J., *Estimating and comparing adverse event probabilities in the presence of varying follow-up times and competing events*, Pharmaceutical Statistics, 2021;1-22

[13] Aalen O.O., Søren J., *An empirical transition matrix for non-homogeneous Markov chains based on censored observations*, Scandinavian Journal of Statistics,1978 5(3); 141-150

[14] Coviello V., Boggess M., *Cumulative incidence estimation in the presence of competing risks*, The Stata Journal, 2004 4(2); 103-112

[15] Kenward, M.G., Roger J.H., *Small sample inference for fixed effects from restricted maximum likelihood*, Biometrics, 1997 53; 983-997

[16] Liu Q., Shepard B.E., Li C., Harrell F.E., *Modelling continuous response variables using ordinal regression*, Statistics in Medicine, 2017 36(27); 4316-4335

Royston, P., and M. K. B. Parmar. 2002. Flexible parametric proportional-hazards and proportional-odds models for censored survival data, with application to prognostic modelling and estimation of treatment effects. Statistics in Medicine 21: 2175–2197.

# Appendix A - Data processing rules and self-evident corrections

Self-evident corrections (SECs) and Miscellaneous Data Anomalies to be read in conjunction with the VenUS 6 Validation Plans. The below rules relate to errors or omissions in case report forms returned to the York Trials Unit (YTU) which will be corrected without the need to raise a data query with the trial co-ordinators. These will be limited to cases where there is no ambiguity. Sites will be made aware of these changes through a centralised file note which will be provided to sites at the end of recruitment.

**General Data Processing Decision Rules**

| No. | CRF | Query | YTU Action |
|---|---|---|---|
| 1 | All CRFs | Where a YES/NO or OTHER response is missing but data has been given to justify response | Where there is no ambiguity DM will apply SECs to correct response |
| 2 | All CRFs | Date provided in incorrect or erroneous format | Where there is no ambiguity, DM will apply SECs to correct response |
| 3 | All CRFs | Text provided for a numeric field | DM will apply SEC to convert response to a number |
| 4 | All CRFs | Date CRF being completed is missing | Where CRF is completed in clinic, the date of clinic will be used and DM will add this during processing<br>Where CRF is completed remotely (e.g. Participant M1, M3, M6 and M12) the date 2 days before CRF received will be used and DM will add this during processing |
| 5 | All CRFs | Data entries provided in a response where a related checkbox or question preceding it has not been completed. | If there is no ambiguity with regard to the description of the responses, DM will complete the appropriate preceding box/question and use the data provided, otherwise queried |
| 6 | All CRFs | Where a response is No, but 0s are given in number of times fields | Where there is no ambiguity 0s will be removed by DM during processing |

| | | | |
|---|---|---|---|
| 7 | All CRFs | Spelling errors with regard to any text entry | Obvious spelling errors (e.g. drug and disease names) will be amended by DM to the correct term, maintaining the original meaning, otherwise queried. |
| 8 | All CRFs | Name is missing but signature present | Where there is no ambiguity, DM will apply SECs to correct response |
| 9 | All CRFs | Site ID not provided | If no ambiguity about the site to which the participant belongs, DM will complete site ID, otherwise queried. |
| 17 | All CRFs | Assessor ID does not match signature on CRF | If the assessor ID does not match the signature on the CRF (as determined by the site delegation log), DM will amend the ID to match the signature, otherwise queried. |

## Screening Form

| | Variable | Validation plan rules | Processing rule |
|---|---|---|---|
| 1 | Exc4_Date | Not Null if Exc4 = 1 and Exc4_NA is Null; else Null | If Exc4_Date is incomplete (e.g. month and year are given, but day is missing), remove Exc4_Date and set Exc4_NA = 1 |
| 2 | Exc4_NA | Not Null if Exc4 = 1 and Exc4_Date is Null; else Null | If Exc4 = 1 and both Exc4_Date and Exc4_NA are missing, set Exc4_NA = 1 |
| 3 | PtNonCon4 | 1 or Null | If PtInfCon = 2 and PtNonCon1/2/3/4 are all missing, then set PtNonCon4 = 1 |

## Investigator Baseline CRF

| | Variable | Validation plan rules | Processing rule |
|---|---|---|---|
| 1 | Diabetes | Range 1 - 2; Not Null. If = 1 DiabetesType should be completed | If Diabetes is missing, but DiabetesType = 1 or 2, set Diabetes = 1 |

| | | | |
|---|---|---|---|
| 2 | Surg | Range 1 - 2; Not Null. If = 1 Surg1 to SurgOth2 should be completed and one or more should = 1 | Where the response to Surg = 2 and Surg1/2/3/4/Oth = 2, Where there is no ambiguity DM will apply SEC to remove No response to each surgery type |
| 3 | Surg1/2/3/4 | Range 1 - 2; Not Null if Surg = 1. If = 1 SurgX_Date should be completed | If Surg = 1 and at least one of Surg1/2/3/4/Oth1/Oth2= 1 and SurgX and SurgX_Date are missing, assume SurgX = 2 |
| 4 | Surg1/2/3/4_Date | Not Null if SurgX = 1; else Null<br>Valid date<br>Before today and after recruitment start date | If SurgX_Date is incomplete (i.e. the year is given, but the month is missing) then assume the month is 06, unless this means SurgX_Date is later than DateComp, in which case assume the month is 01 |
| 5 | SurgOth1/2 | Range 1 - 2; Not Null if Surg = 1. If = 1 SurgOthX_Date should be completed | If Surg = 1 and at least one of Surg1/2/3/4/Oth1/Oth2= 1 and SurgOthX_Info, SurgOthX and SurgOthX_Date are missing, assume SurgOthX = 2 |
| 6 | SurgOth1/2_Date | Not Null if SurgOthX = 1; else Null<br>Valid date<br>Before today and after recruitment start date | If SurgOthX_Date is incomplete (i.e. the year is given, but the month is missing) then assume the month is 06, unless this means SurgOthX_Date is later than DateComp, in which case assume the month is 01 |
| 7 | RUA4y | Range 0 – 10; Not Null | If RUA4y is missing and RUA4m is completed and is greater than 0, assume RUA4y = 0 |
| 8 | RUA4m | Range 0 – 11; Not Null | If RUA4m is missing and RUA4y is completed and is greater than 0, assume RUA4m = 0 |
| 9 | Weight_lbs | Range 0 - 13; Not Null if Weight_kg is Null | If Weight_st is completed and Weight_lbs is missing, then assume Weight_lbs = 0 |
| 10 | Height_in | Range 0 - 11; Not Null if Height_cm is Null | If Height_ft is completed and Height_in is missing, then assume Height_in = 0 |
| 11 | DateRand | Not Null; Valid date; Before today and after recruitment start date; Matches ParticipantID | If DateRand is not provided or is incorrect DM will check against management system and apply a SEC |

| | | | |
|---|---|---|---|
| 12 | Treat2a | Range 1 - 3; Not Null<br>If = 2 Treat2a_Info should be completed | If Treat1 = 2 and Treat2 - Treat2_14b are all missing and Treat2a is missing, assume Treat2a = 3 |

## Participant Baseline CRF

| | Variable | Validation plan rules | Processing rule |
|---|---|---|---|
| 1 | Gender | Range 1 - 3; Not Null | If multiple options selected, assume Gender = missing |
| 2 | Ethnic | Range 1 - 6; Not Null<br>If = 6 EthnicOth should be completed | If multiple options selected, assume Ethnic = missing |
| 3 | SmkStatus | Range 1 – 3; Not Null | If multiple options selected, assume SmkStatus = missing |
| 4 | Employ | Range 1 - 6; Not Null<br>If = 1 EmployType should be completed<br>If = 6 EmployOth should be completed | If multiple options selected, assume Employ = missing, unless additional information is given to support a particular option (e.g EmployType = 1 or 2) |
| 5 | DateComp | Not Null<br>Valid date<br>Before today and after recruitment start date<br>On or after DateVisit | If DateComp is missing, assume DateComp = DateVisit |
| 6 | UlcerLoc | Range 1 - 3; Not Null<br>If = 1 or 2 UlcerNum to UlcerPain_Oth should be completed<br>If = 3 UlcerNum to UlcerPain_Oth should be Null | If multiple options selected choose "worst" option (i.e. 2 is chosen over 1 or 3 and 1 is chosen over 3) |
| 7 | UlcerNum | Range 1 – 10 | If UlcerLoc = 3 and UlcerNum = 0, set UlcerNum = missing |

| | | | |
|---|---|---|---|
| | | Not null if UlcerLoc = 1 or 2; else Null | |
| 8 | UlcerScore | Range 0 – 100<br>Not null if UlcerLoc = 1 or 2; else Null | If UlcerLoc = 3 and UlcerScore = 0, set UlcerScore = missing<br>If two values selected on VAS, Set UlcerScore to middle/average value from range indicated<br>If range of values indicated, Set UlcerScore to middle/average value from range indicated |
| 9 | UlcerScore | Range 0 – 100<br>Not null if UlcerLoc = 1 or 2; else Null | Response marked on line but not annotated in associated box. Where response is marked on the line, DM will add the value to the associated box during processing. |
| 10 | UlcerPain | Range 1 – 6<br>Not null if UlcerLoc = 1 or 2; else Null<br>If = 6 UlcerPain_Oth should be completed | If exactly one of the options 1 to 5 is selected and option 6 (i.e. "Other") is selected, then assume UlcerPain is equal to the value selected between 1 and 5 (i.e. ignore response of "Other"), but leave any details given in UlcerPain_Oth<br>If exactly two of options 1 to 5 are selected (e.g. 2 and 3), choose the worse option (i.e. lower scoring option). If option 6 (i.e. "Other") is also selected, then ignore this, but leave any details given in UlcerPain_Oth<br>If more than two of options 1 to 5 are selected then ignore these responses, and set UlcerPain = missing if option 6 is not selected, or set UlcerPain = 6 if option 6 is selected |
| 11 | EQ5D_0X_5L | Range 1 - 5; Not Null | Standard rules for ambiguous data in EQ-5D (i.e. treat as missing) |
| 12 | EQ5D_THERM_5L | Range 0 - 100; Not Null | Standard rules for ambiguous data in EQ-5D (i.e. treat as missing) |
| 13 | EQ5D_THERM_5L | Range 0 - 100; Not Null | Response marked on line but not annotated in associated box Where response is marked on the line, DM will add the value to the associated box during processing. |
| 14 | VEINES_01 – VEINES_09 | Range 1 – 5; Not Null | If exactly two options with adjacent scores are selected, choose the worse option (i.e. lower scoring option) |

| | | | |
|---|---|---|---|
| | | | If two options with non-adjacent scores are selected, set VEINES_X = missing<br>If more than two options are selected, set VEINES_X = missing |
| 15 | VEINES_10 | Range 1 – 6; Not Null | If more than one option is selected, set VEINES_10 = missing |
| 16 | VEINES_11 | Range 1 – 6; Not Null | If exactly two options with adjacent scores are selected, choose the worse option (i.e. higher scoring option)<br>If two options with non-adjacent scores are selected, set VEINES_11 = missing<br>If more than two options are selected, set VEINES_11 = missing |
| 17 | VEINES_12 | Range 0 – 3; Not Null | If more than one option is selected, set VEINES_12 = missing |
| 18 | VEINES_13 – VEINES_15 | Range 1 – 3; Not Null | If exactly two options with adjacent scores are selected, choose the worse option (i.e. lower scoring option)<br>If two options with non-adjacent scores are selected, set VEINES_X = missing<br>If more than two options are selected, set VEINES_X = missing |
| 19 | VEINES_16 – VEINES_19 | Range 1 – 2; Not Null | If both options selected, choose worse option (i.e. lower scoring option) |
| 20 | VEINES_20 | Range 1 – 5; Not Null | If exactly two options with adjacent scores are selected, choose the worse option (i.e. higher scoring option)<br>If two options with non-adjacent scores are selected, set VEINES_20 = missing<br>If more than two options are selected, set VEINES_20 = missing |
| 21 | VEINES_21 | Range 1 – 6; Not Null | If exactly two options with adjacent scores are selected, choose the worse option (i.e. higher scoring option)<br>If two options with non-adjacent scores are selected, set VEINES_21 = missing |

| | | | |
|---|---|---|---|
| | | | If more than two options are selected, set VEINES_21 = missing |
| 22 | VEINES_22 – VEINES_26 | Range 1 – 6; Not Null | If exactly two options with adjacent scores are selected, choose the worse option (i.e. lower scoring option)<br>If two options with non-adjacent scores are selected, set VEINES_X = missing<br>If more than two options are selected, set VEINES_X = missing |
| 23 | TreatPref | Range 1 – 4; Not Null | If more than one option selected, set TreatPref = missing |

## Adverse Event Initial Report Form

| | Variable | Validation plan rules | Processing rule |
|---|---|---|---|
| 1 | OnsetDate | Not Null<br>Valid date<br>Before today and after recruitment start date | If the month and year of OnsetDate are given, but the day is missing, assume the day is 15 (i.e. OnsetDate is the middle of the month indicated), except if this makes OnsetDate after EndDate, in which case assume the day is 01 (i.e. OnsetDate is the beginning of the month indicated) |
| 2 | OnsetTime | Valid time if completed. Can be Null | If the hour is given, but the minutes are missing, assume the minutes are 00 (i.e. OnsetTime was 0 minutes past the hour indicated |
| 3 | EndDate | Not Null if Outcome = 1 or 3; else Null<br>Valid date<br>On or after OnsetDate<br>Before today and after recruitment start date | If the month and year of EndDate are given, but the day is missing, assume the day is 15 (i.e. EndDate is the middle of the month indicated), unless this make EndDate after OnsetDate, in which case assume the day is the last day of the month (i.e. EndDate is the end of the month indicated) |
| 4 | EndTime | Valid time if completed. Can be Null. After OnsetTime if OnsetDate = EndDate | If the hour is given, but the minutes are missing, assume the minutes are 00 (i.e. EndTime was 0 minutes past the hour indicated |

## Event Form

| | Variable | Validation plan rules | Processing rule |
|---|---|---|---|
| 1 | DateUlcDeter1/2 | Not Null if UlcDeterX = 1; else Null<br>Valid date<br>Before today and after recruitment start date<br>On or before DateComp | If DateUlcDeterX is incomplete (i.e. month and year are given, but day is missing), assume day = 15, unless this is after DateComp, in which case assume day = 01 |
| 2 | DateDisc | Not Null if DiscHosp = 1; else Null<br>Valid date<br>Before today and after recruitment start date<br>On or before DateComp<br>After DateAdmit | If DateDisc is incomplete (i.e.month and year are given, but day is missing), assume day = 15, unless this is before DateAdmit, in which case assume day is the last day of the month |
| 3 | DateUlcInfec | Not Null if UlcInfec1 = 1; else Null<br>Valid date<br>Before today and after recruitment start date<br>On or before DateComp | If DateUlcInfec is incomplete (i.e. month and year are given, but day is missing), assume day = 15, unless this is after DateComp, in which case assume day = 01 |
| 4 | DateNewUlc | Not Null if NewUlc = 1; else Null<br>Valid date<br>Before today and after recruitment start date<br>On or before DateComp | If DateNewUlc is incomplete (i.e. month and year are given, but day is missing), assume day = 15, unless this is after DateComp, in which case assume day = 01 |

## Visit Log

| | Variable | Validation plan rules | Processing rule |
|---|---|---|---|
| 1 | VisitDur1 | Valid time<br>Range 10 mins to 2 hours<br>Not Null if DateComp1 is completed | If minutes are given, but hours are missing, assume number of hours is 00<br>If hours are given and are greater than 0,= and minutes are missing, assume number of minutes in 00 |
| 2 | NewWrap1 | Range 1 - 2<br>Not Null if Activ1 = 1 or 3 and NoItems1 is Null<br>If = 1 NewWrap1_1 should be completed | If NewWrap1 = 2 and Type1 = 3, 4, 5, 6, 7, 8, 9 or 10, set NewWrap1 = missing |
| 3 | NewBand1 | Range 1 - 2<br>Not Null if Activ1 = 1 or 3 and NoItems1 is Null<br>If = 1 NewBand1_1 should be completed | If NewBand1 = 2 and Type1 = 1, 2, 7, 8, 9 or 10, set NewBand1 = missing |
| 4 | NewHos1 | Range 1 - 2<br>Not Null if Activ1 = 1 or 3 and NoItems1 is Null<br>If = 1 NewHos1_1 and NewHos1_2 should be completed | If NewHos1 = 2 and Type1 = 1, 2, 3, 4, 5 or 6, set NewHos1 = missing |

## Participant 1 Month Follow Up CRF

| | Variable | Validation plan rules | Processing rule |
|---|---|---|---|
| 1 | DateComp | Not Null<br>Valid date<br>Before today and after recruitment start date<br>On or after DateSent | If DateComp is missing, set to two days before date returned to YTU |
| 2 | UlcerLoc | Range 1 - 3; Not Null<br>If = 1 or 2 UlcerNum to UlcerPain_Oth should be completed<br>If = 3 UlcerNum to UlcerPain_Oth should be Null | If multiple options selected choose "worst" option (i.e. 2 is chosen over 1 or 3 and 1 is chosen over 3) |
| 3 | UlcerNum | Range 1 – 20<br>Not null if UlcerLoc = 1 or 2; else Null | If UlcerLoc = 3 and UlcerNum = 0, set UlcerNum = missing |
| 4 | UlcerScore | Range 0 – 100<br>Not null if UlcerLoc = 1 or 2; else Null | If UlcerLoc = 3 and UlcerScore = 0, set UlcerScore = missing<br>If two values selected on VAS, Set UlcerScore to middle/average value from range indicated<br>If range of values indicated, Set UlcerScore to middle/average value from range indicated |
| 5 | UlcerScore | Range 0 – 100<br>Not null if UlcerLoc = 1 or 2; else Null | Response marked on line but not annotated in associated box. Where response is marked on the line, DM will add the value to the associated box during processing. |
| 6 | UlcerPain | Range 1 – 6<br>Not null if UlcerLoc = 1 or 2; else Null<br>If = 6 UlcerPain_Oth should be completed | If exactly one of the options 1 to 5 is selected and option 6 (i.e. “Other”) is selected, then assume UlcerPain is equal to the value selected between 1 and 5 (i.e. ignore response of “Other”), but leave any details given in UlcerPain_Oth<br>If exactly two of options 1 to 5 are selected (e.g. 2 and 3), choose the worse option (i.e. lower scoring option). If option 6 (i.e. “Other”) |

| | | | |
|---|---|---|---|
| | | | is also selected, then ignore this, but leave any details given in UlcerPain_Oth<br>If more than two of options 1 to 5 are selected then ignore these responses, and set UlcerPain = missing if option 6 is not selected, or set UlcerPain = 6 if option 6 is selected |
| 7 | CT_01 | Range 1 – 5<br>Not null if CT_Prev = 1 or 2; else Null | If exactly two options with adjacent scores selected, choose "worse" response (i.e. higher scoring response)<br>If two options with non-adjacent scores selected, set CT_01 = missing<br>If more than two options selected, set CT_01 = missing |
| 8 | CT_02Score | Range 0 – 100<br>Not null if PT_Prev = 1 or 2; else Null | If CT_Prev = 3 and CT_02Score = 0, set CT_02Score = missing |
| 9 | CT_03b | 1 or Null<br>If = 1 CT_03b_2 should be completed | If CT_03b is missing and CT_03b_2 is not missing, set CT_03b = 1 |
| 10 | CT_03b_2 | Range 1 – 5<br>Not null if CT_03b = 1; else Null | If two options with adjacent scores selected, choose "worse" response (i.e. higher scoring response)<br>If two options with non-adjacent scores selected, set CT_03b_2 = missing<br>If more than two options selected, set CT_03b_2 = missing |
| 11 | CT_03c | 1 or Null.<br>If = 1 CT_03c_2 should be completed | If CT_03c is missing and CT_03c_2 is not missing, set CT_03c = 1 |
| 12 | CT_03c_2 | Range 1 – 5<br>Not null if CT_03c = 1; else Null | If two options with adjacent scores selected, choose "worse" response (i.e. higher scoring response)<br>If two options with non-adjacent scores selected, set CT_03c_2 = missing<br>If more than two options selected, set CT_03c_2 = missing |

| | | | |
|---|---|---|---|
| 13 | CT_03d | 1 or Null.<br>If = 1 CT_03d_2 should be completed | If CT_03d is missing and CT_03d_2 is not missing, set CT_03d = 1 |
| 14 | CT_03d_2 | Range 1 – 5<br>Not null if CT_03d = 1; else Null | If two options with adjacent scores selected, choose "worse" response (i.e. higher scoring response)<br>If two options with non-adjacent scores selected, set CT_03d_2 = missing<br>If more than two options selected, set CT_03d_2 = missing |
| 15 | CT_06 | Range 1 – 5<br>Not null if CT_Prev = 1 or 2; else Null | If multiple options selected, assume CT_06 = missing |
| 16 | CT_07 | Range 2 – 5<br>Not null if CT_Prev = 1 or 2; else Null | If multiple options selected, assume CT_07 = missing |
| 17 | CT_08 | Range 2 – 5<br>Not null if CT_Prev = 1 or 2; else Null | If multiple options selected, assume CT_08 = missing |
| 18 | CT_09a | Range 1 – 4<br>Not Null if CT_Prev = 1 or 2 and MDB AllocationID = 3 ; else Null | If multiple options selected, assume CT_09a = missing |
| 19 | CT_09b | Range 1 – 4<br>Not Null if CT_Prev = 1 or 2 and MDB AllocationID = 3 ; else Null | If multiple options selected, assume CT_09b = missing |
| 20 | EQ5D_0X_5L | Range 1 - 5; Not Null | Standard rules for ambiguous data in EQ-5D (i.e. treat as missing) |
| 21 | EQ5D_THERM_5L | Range 0 - 100; Not Null | Standard rules for ambiguous data in EQ-5D (i.e. treat as missing) |
| 22 | EQ5D_THERM_5L | Range 0 - 100; Not Null | EQ5D thermometer/VAS line: Response marked on line but not annotated in associated box. Where response is marked on the line, DM will add the value to the associated box during processing. |

## Participant 3, 6 and 12 Month Follow Up CRF

| | Variable | Validation plan rules | Processing rule |
|---|---|---|---|
| 1 | DateComp | Not Null<br>Valid date<br>Before today and after recruitment start date<br>On or after DateSent | If DateComp is missing, set to two days before date returned to YTU |
| 2 | UlcerLoc | Range 1 - 3; Not Null<br>If = 1 or 2 UlcerNum to UlcerPain_Oth should be completed<br>If = 3 UlcerNum to UlcerPain_Oth should be Null | If multiple options selected choose "worst" option (i.e. 2 is chosen over 1 or 3 and 1 is chosen over 3) |
| 3 | UlcerNum | Range 1 – 20<br>Not null if UlcerLoc = 1 or 2; else Null | If UlcerLoc = 3 and UlcerNum = 0, set UlcerNum = missing |
| 4 | UlcerScore | Range 0 – 100<br>Not null if UlcerLoc = 1 or 2; else Null | If UlcerLoc = 3 and UlcerScore = 0, set UlcerScore = missing<br>If two values selected on VAS, Set UlcerScore to middle/average value from range indicated<br>If range of values indicated, Set UlcerScore to middle/average value from range indicated |
| 5 | UlcerScore | Range 0 – 100<br>Not null if UlcerLoc = 1 or 2; else Null | Response marked on line but not annotated in associated box. Where response is marked on the line, DM will add the value to the associated box during processing. |
| 6 | UlcerPain | Range 1 – 6<br>Not null if UlcerLoc = 1 or 2; else Null<br>If = 6 UlcerPain_Oth should be completed | If exactly one of the options 1 to 5 is selected and option 6 (i.e. “Other”) is selected, then assume UlcerPain is equal to the value selected between 1 and 5 (i.e. ignore response of “Other”), but leave any details given in UlcerPain_Oth<br>If exactly two of options 1 to 5 are selected (e.g. 2 and 3), choose the worse option (i.e. lower scoring option). If option 6 (i.e. “Other”) |

| | | | |
|---|---|---|---|
| | | | is also selected, then ignore this, but leave any details given in UlcerPain_Oth<br>If more than two of options 1 to 5 are selected then ignore these responses, and set UlcerPain = missing if option 6 is not selected, or set UlcerPain = 6 if option 6 is selected |
| 7 | CT_01 | Range 1 – 5<br>Not null if CT_Prev = 1 or 2; else Null | If exactly two options with adjacent scores selected, choose "worse" response (i.e. higher scoring response)<br>If two options with non-adjacent scores selected, set CT_01 = missing<br>If more than two options selected, set CT_01 = missing |
| 8 | CT_02Score | Range 0 – 100<br>Not null if PT_Prev = 1 or 2; else Null | If CT_Prev = 3 and CT_02Score = 0, set CT_02Score = missing |
| 9 | CT_03b | 1 or Null<br>If = 1 CT_03b_2 should be completed | If CT_03b is missing and CT_03b_2 is not missing, set CT_03b = 1 |
| 10 | CT_03b_2 | Range 1 – 5<br>Not null if CT_03b = 1; else Null | If two options with adjacent scores selected, choose "worse" response (i.e. higher scoring response)<br>If two options with non-adjacent scores selected, set CT_03b_2 = missing<br>If more than two options selected, set CT_03b_2 = missing |
| 11 | CT_03c | 1 or Null.<br>If = 1 CT_03c_2 should be completed | If CT_03c is missing and CT_03c_2 is not missing, set CT_03c = 1 |
| 12 | CT_03c_2 | Range 1 – 5<br>Not null if CT_03c = 1; else Null | If two options with adjacent scores selected, choose "worse" response (i.e. higher scoring response)<br>If two options with non-adjacent scores selected, set CT_03c_2 = missing<br>If more than two options selected, set CT_03c_2 = missing |

| | | | |
|---|---|---|---|
| 13 | CT_03d | 1 or Null.<br>If = 1 CT_03d_2 should be completed | If CT_03d is missing and CT_03d_2 is not missing, set CT_03d = 1 |
| 14 | CT_03d_2 | Range 1 – 5<br>Not null if CT_03d = 1; else Null | If two options with adjacent scores selected, choose "worse" response (i.e. higher scoring response)<br>If two options with non-adjacent scores selected, set CT_03d_2 = missing<br>If more than two options selected, set CT_03d_2 = missing |
| 15 | CT_06 | Range 1 – 5<br>Not null if CT_Prev = 1 or 2; else Null | If multiple options selected, assume CT_06 = missing |
| 16 | CT_07 | Range 2 – 5<br>Not null if CT_Prev = 1 or 2; else Null | If multiple options selected, assume CT_07 = missing |
| 17 | CT_08 | Range 2 – 5<br>Not null if CT_Prev = 1 or 2; else Null | If multiple options selected, assume CT_08 = missing |
| 18 | CT_09a | Range 1 – 4<br>Not Null if CT_Prev = 1 or 2 and MDB AllocationID = 3 ; else Null | If multiple options selected, assume CT_09a = missing |
| 19 | CT_09b | Range 1 – 4<br>Not Null if CT_Prev = 1 or 2 and MDB AllocationID = 3 ; else Null | If multiple options selected, assume CT_09b = missing |
| 20 | EQ5D_0X_5L | Range 1 - 5; Not Null | Standard rules for ambiguous data in EQ-5D (i.e. treat as missing) |
| 21 | EQ5D_THERM_5L | Range 0 - 100; Not Null | Standard rules for ambiguous data in EQ-5D (i.e. treat as missing) |
| 22 | EQ5D_THERM_5L | Range 0 - 100; Not Null | EQ5D thermometer/VAS line: Response marked on line but not annotated in associated box. Where response is marked on the line, DM will add the value to the associated box during processing. |

| | | | |
|---|---|---|---|
| 23 | VEINES_01 – VEINES_09 | Range 1 – 5; Not Null | If exactly two options with adjacent scores are selected, choose the worse option (i.e. lower scoring option)<br>If two options with non-adjacent scores are selected, set VEINES_X = missing<br>If more than two options are selected, set VEINES_X = missing |
| 24 | VEINES_10 | Range 1 – 6; Not Null | If more than one option is selected, set VEINES_10 = missing |
| 25 | VEINES_11 | Range 1 – 6; Not Null | If exactly two options with adjacent scores are selected, choose the worse option (i.e. higher scoring option)<br>If two options with non-adjacent scores are selected, set VEINES_11 = missing<br>If more than two options are selected, set VEINES_11 = missing |
| 26 | VEINES_12 | Range 0 – 3; Not Null | If more than one option is selected, set VEINES_12 = missing |
| 27 | VEINES_13 – VEINES_15 | Range 1 – 3; Not Null | If exactly two options with adjacent scores are selected, choose the worse option (i.e. lower scoring option)<br>If two options with non-adjacent scores are selected, set VEINES_X = missing<br>If more than two options are selected, set VEINES_X = missing |
| 28 | VEINES_16 – VEINES_19 | Range 1 – 2; Not Null | If both options selected, choose worse option (i.e. lower scoring option) |
| 29 | VEINES_20 | Range 1 – 5; Not Null | If exactly two options with adjacent scores are selected, choose the worse option (i.e. higher scoring option)<br>If two options with non-adjacent scores are selected, set VEINES_20 = missing<br>If more than two options are selected, set VEINES_20 = missing |
| 30 | VEINES_21 | Range 1 – 6; Not Null | If exactly two options with adjacent scores are selected, choose the worse option (i.e. higher scoring option) |

| | | | |
|---|---|---|---|
| | | | If two options with non-adjacent scores are selected, set VEINES_21 = missing<br>If more than two options are selected, set VEINES_21 = missing |
| 31 | VEINES_22 – VEINES_26 | Range 1 – 6; Not Null | If exactly two options with adjacent scores are selected, choose the worse option (i.e. lower scoring option)<br>If two options with non-adjacent scores are selected, set VEINES_X = missing<br>If more than two options are selected, set VEINES_X = missing |

# Appendix B – Trial flow diagram

**Figure xx:** Participant flow diagram (reference ulcer healing)

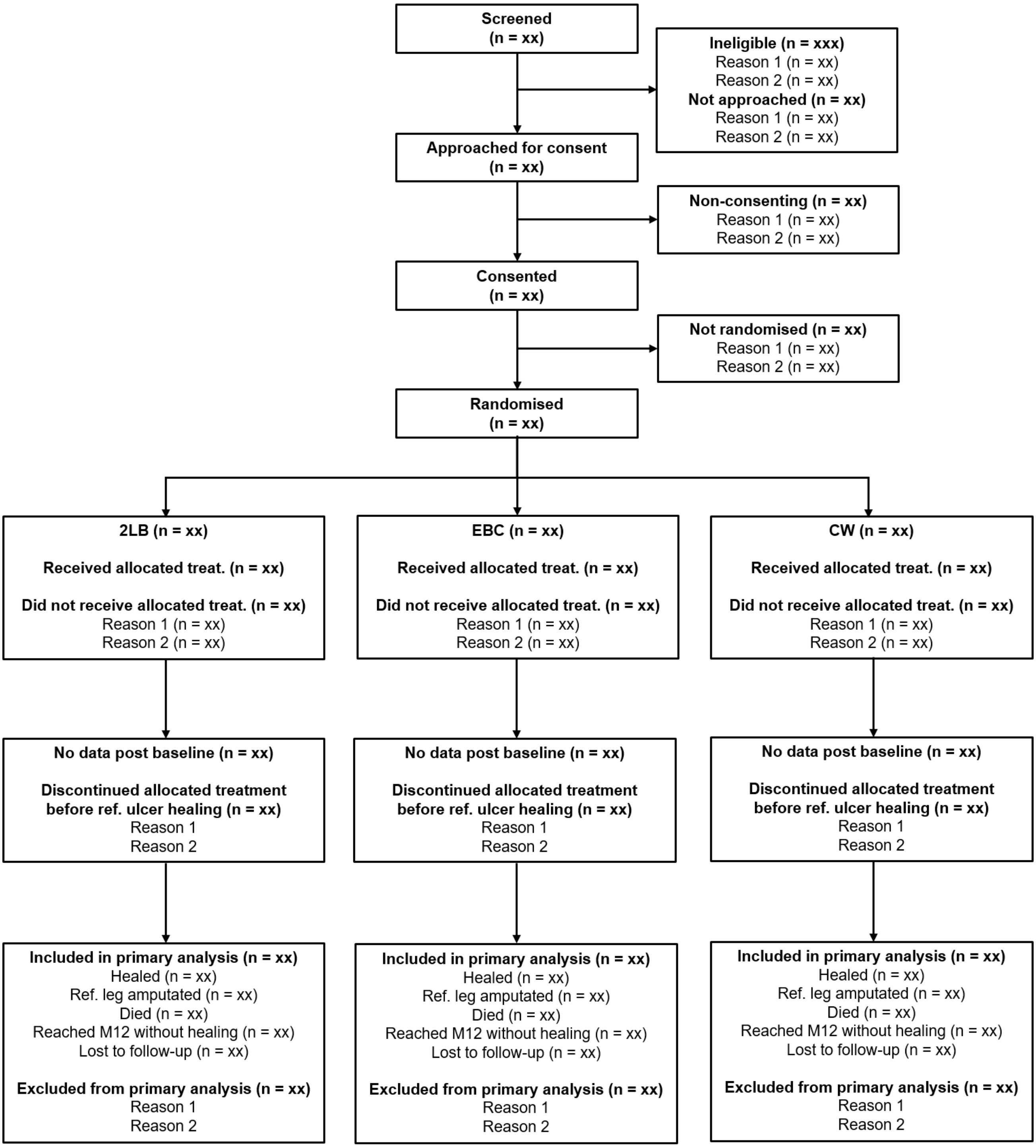

**Figure xx:** Participant flow diagram (participant complete follow ups)

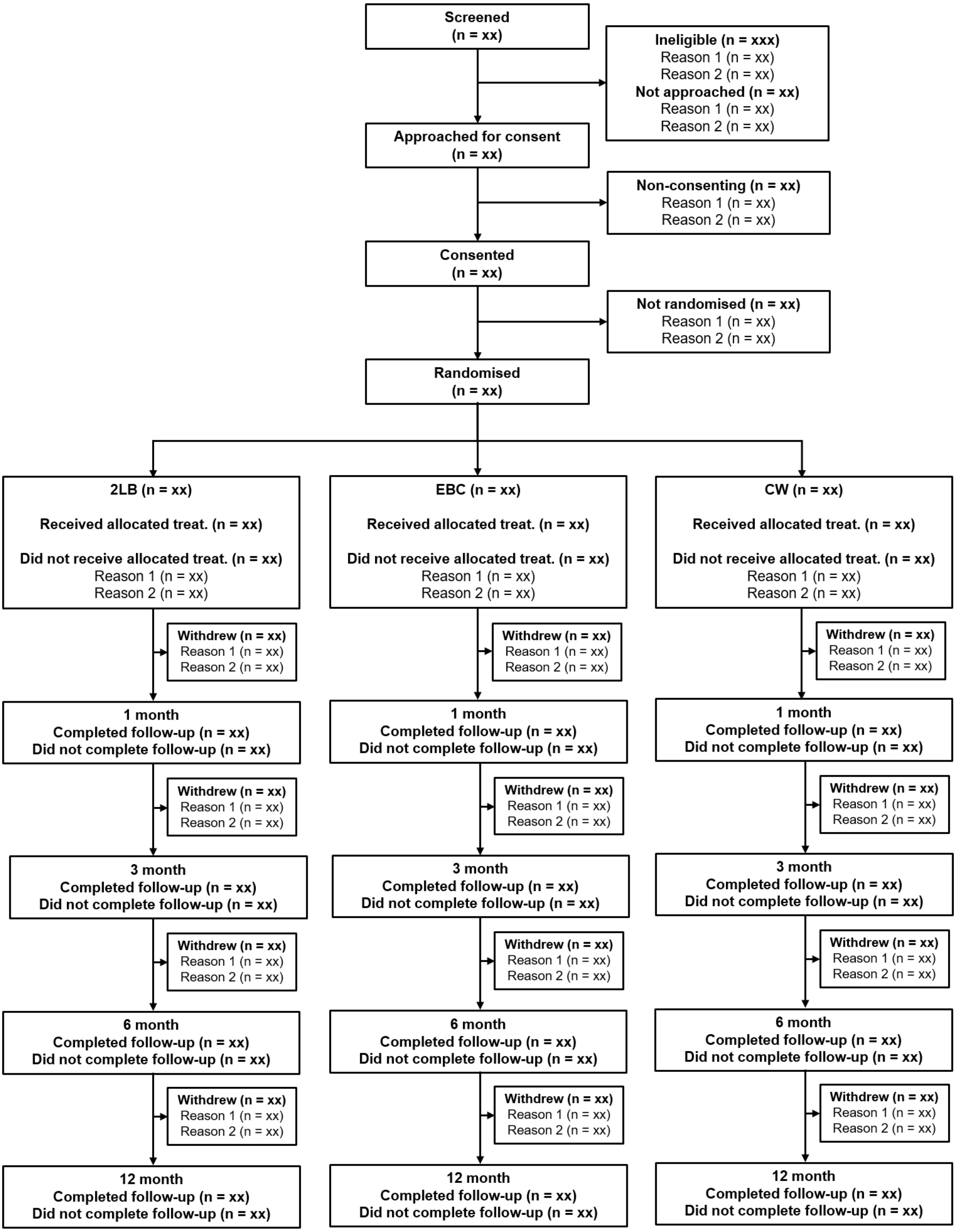

Supplement: S6 File — (PDF) [file pmed.1005154.s006.pdf]
