# Supplementary material for: Compression therapies for venous leg ulcers: The VENous Ulcer Study 6 (VenUS 6), an open, multicentre, randomised clinical trial
Source: PLoS Med. 2026 Jul 10;23(7):e1005154. doi: 10.1371/journal.pmed.1005154 (PMC13354008; doi:10.1371/journal.pmed.1005154)
Supplement: Attachment — Submitted filename: Response to Editorial requests 02.02.26.docx [file pmed.1005154.s009.docx]

|  | **EDITORIAL** | |
| --- | --- | --- |
| 1. | *       We ask every co-author listed on the manuscript to fill in a contributing author statement, making sure to declare all competing interests. If any of the co-authors have not filled in the statement, we will remind them to do so when the paper is revised. If all statements are not completed in a timely fashion this could hold up the re-review process. If new competing interests are declared later in the revision process, this may also hold up the submission. Should there be a problem getting one of your co-authors to fill in a statement we will be in contact. Please do not add or remove authors without first discussing this with the handling editor. You can see our competing interests policy here: <http://journals.plos.org/plosmedicine/s/competing-interests>. | We will be happy to help to support completing of any outstanding forms. If there are any please do let me know. |
| 2. | *       Please upload any figures associated with your paper as individual TIF or EPS files with 300dpi resolution at resubmission; please read our figure guidelines for more information on our requirements: <http://journals.plos.org/plosmedicine/s/figures>. While revising your submission, we strongly recommend that you use PLOS's NAAS tool (<https://ngplosjournals.pagemajik.ai/artanalysis>) to test your figure files. NAAS can convert your figure files to the TIFF file type and meet basic requirements (such as print size, resolution), or provide you with a report on issues that do not meet our requirements and that NAAS cannot fix. | Figures associated with the manuscript are uploaded as TIF figures. |
| 3. | *       Please ensure that the paper adheres to the PLOS Data Availability Policy (see <http://journals.plos.org/plosmedicine/s/data-availability>), which requires that all data underlying the study's findings be provided in a repository or as Supporting Information. For data residing with a third party, authors are required to provide instructions with contact information (web or email address) for obtaining the data. Please note that a study author cannot be the contact person for the data. PLOS journals do not allow statements supported by "data not shown" or "unpublished results." For such statements, authors must provide supporting data or cite public sources that include it. | We have included a data sharing statement at the end of the manuscript and will make anonymised data sets available via a repository (OSF) following publication. |
| 4. | *       We expect all researchers with submissions to PLOS in which author-generated code underpins the findings in the manuscript to make all author-generated code available without restrictions upon publication of the work. In cases where code is central to the manuscript, we may require the code to be made available as a condition of publication. Authors are responsible for ensuring that the code is reusable and well documented. Please make any custom code available, either as part of your data deposition or as a supplementary file. Please add a sentence to your data availability statement regarding any code used in the study, e.g. "The code used in the analysis is available from Github [URL] and archived in Zenodo [DOI link]" Please review our guidelines at <https://journals.plos.org/plosmedicine/s/materials-software-and-code-sharing> and ensure that your code is shared in a way that follows best practice and facilitates reproducibility and reuse. Because Github depositions can be readily changed or deleted, we encourage you to make a permanent DOI'd copy (e.g. in Zenodo) and provide the URL. | We will make anonymised data and the associated code available on publication of the paper via OSF. We have updated our data sharing statement to reflect that this will include both components. |
| 5. | *       In the Ethics statement in the Methods section, please clearly state whether informed consent was written or oral. | We have clarified that consent was written on Page 6. |
| 6. | FORMATTING - GENERAL *       Abstract: Please structure your abstract using the PLOS Medicine headings (Background, Methods and Findings, Conclusions). Please combine the Methods and Findings sections into one section.  *       At this stage, we ask that you include a short, non-technical Author Summary of your research to make findings accessible to a wide audience that includes both scientists and non-scientists. The Author Summary should immediately follow the Abstract in your revised manuscript. This text is subject to editorial change and should be distinct from the scientific abstract. Ideally each sub-heading should contain 2-3 single sentence, concise bullet points containing the most salient points from your study. In the final bullet point of 'What Do These Findings Mean?', please include the main limitations of the study in non-technical language. Please see our author guidelines for more information: <https://journals.plos.org/plosmedicine/s/revising-your-manuscript#loc-author-summary>.  *       Please express the main results with 95% CIs as well as p values. When reporting p values please report as p<0.001 and where higher as the exact p value p=0.002, for example. Throughout, suggest reporting statistical information as follows to improve clarity for the reader "22% (95% CI [13%,28%]; p</=)". Please be sure to define all numerical values at first use.  *       Please include page numbers and line numbers in the manuscript file. Use continuous line numbers (do not restart the numbering on each page).  *       Please cite the reference numbers in square brackets. Citations should precede punctuation. | The abstract is structured as requested.  We have added an author summary to the end of the abstract as requested.  We have checked to ensure p values are reported for the main results.  Page and continuous line numbers are present in the manuscript.  Referencing has been converted to square brackets as requested |
| 7. | FIGURES AND TABLES *       Please provide titles and legends for all figures and tables (including those in Supporting Information files).  *       Please define all abbreviations used in each figure/table (including those in Supporting Information files).  *       Please consider avoiding the use of red and green in order to make your figure more accessible to those with color blindness. | Titles/legends for all tables and figures are included at the end of the manuscript.  All abbreviations are defined as footers. |
| 8. | SUPPLEMENTARY MATERIAL *       Please note that supplementary material will be posted as supplied by the authors. Therefore, please amend it according to the relevant comments outlined here.  *       Please cite your Supporting Information as outlined here: <https://journals.plos.org/plosmedicine/s/supporting-information> | Supplementary materials have been amended, if needed, in line with comments elsewhere in this review. |
| 9. | REFERENCES *       PLOS uses the numbered citation (citation-sequence) method and first six authors, et al.  *       Please ensure that journal name abbreviations match those found in the National Center for Biotechnology Information (NCBI) databases (<http://www.ncbi.nlm.nih.gov/nlmcatalog/journals>), and are appropriately formatted and capitalised.  *       Where website addresses are cited, please include the complete URL and specify the date of access (e.g. [accessed: 12/06/2023]).  *       Please also see <https://journals.plos.org/plosmedicine/s/submission-guidelines#loc-references> for further details on reference formatting. | Referencing has been checked to ensure compliance with journal requirements |
| 10. | STUDY TYPE-SPECIFIC REQUESTS *       PLOS Medicine requires that all trials be prospectively registered in one of registries recognized by WHO. Please ensure that study registration details are included in the Methods section.  *       Please structure the Methods section using the following sub-headings: Study design and participants, Randomization and masking, Procedures, Outcomes, Statistical analysis.  *       Please outline any outcome measures that differ between the submitted manuscript and the protocol [and/or trial registry]. Please clarify and explain all discrepancies between the paper and protocol. If the outcomes were not prespecified in the protocol, please define them in the Methods (Outcomes section) as post hoc and explain why they were added. Post-hoc comparisons should be presented as hypothesis generating rather than conclusive.  * Is the outcome on 'resource use' included in the paper?  * Will the semi-structured interviews exploring the views and experiences of participants and nurses on the different compression therapies be reported in a separate paper? If so, please mention this in the Methods section.  *       Please ensure that all prespecified outcomes (primary, secondary, and exploratory) are listed in the Methods/Outcomes section and indicate whether there are outcomes that are not presented in the current report.  *       Please specify the dates (Month Day, Year) during which study enrollment and follow up occurred.  *       Please include absolute numbers wherever you report percentages; eg, n/N (%)  *       Please present the safety data for the study including numbers of specific events and whether or not adverse events are thought to be related to treatment. AEs should be reported in the abstract, per CONSORT and CONSORT-Harms.  *       Please complete the CONSORT checklist (<https://www.equator-network.org/reporting-guidelines/consort/>) and ensure that all components of CONSORT are present in the manuscript, including how randomization was performed, allocation concealment, blinding of intervention, definition of lost to follow-up, power statement. When completing the checklist, please use section and paragraph numbers, rather than page numbers.  * We also encourage authors to complete the SPIRIT 2025 checklist, which highlights the relevant information in the study protocol for readers.  *       Please report your abstract according to CONSORT for abstracts, following the PLOS Medicine abstract structure (Background, Methods and Findings, Conclusions) <https://www.equator-network.org/reporting-guidelines/consort-abstracts/>  *       If your trial had to undergo important modifications in response to extenuating circumstances, please complete the CONSERVE-CONSORT checklist and provide in your Supporting Information; (<https://www.equator-network.org/reporting-guidelines/guidelines-for-reporting-trial-protocols-and-completed-trials-modified-due-to-the-covid-19-pandemic-and-other-extenuating-circumstances-the-conserve-2021-statement/>). When completing the checklist, please use section and paragraph numbers, rather than page numbers.  *       In keeping with our commitment to Open Science, please include the study protocol document and analysis plan (including any amendments) as Supporting Information to be published with the manuscript if accepted.  *       Please note that PLOS Medicine requires prospective, public registration of a data sharing plan (as part of mandatory clinical trials registration) for all clinical trials that began enrollment on or after January 1, 2019, in accordance with ICMJE requirements. | Study registration is included in the Methods section as requested  The structure of the methods section has been amended as requested.  Not applicable, no changes to outcome measures during the trial.  No, this outcome is not included in the manuscript. It is included in a separate cost effectiveness manuscript relating to the trial.  The semi structured interviews will be reported separately. A line has been added to the methods section to reflect this for both papers.  Dates of follow up have been added to the results section.  We have checked and included absolute numbers for percentages.  For this study the clinical outcomes were also collected as adverse events. As a result adverse event reporting is covered in the reporting of that outcome however we have included a summary of serious adverse events reported in the manuscript for completeness.  A CONSORT checklist is provided, updated with section and paragraph numbers.  We thank the journal for this suggestion. A SPIRIT checklist accompanies our published trial protocol manuscript which should fulfil this requirement  The abstract is reported as per the CONSORT Abstract checklist and a copy uploaded with this revision.  Not applicable, no modifications were required.  The protocol and analysis plan are included as Supporting information  We have included a data sharing statement at the end of the manuscript. |
