# Supplementary material for: Compression therapies for venous leg ulcers: The VENous Ulcer Study 6 (VenUS 6), an open, multicentre, randomised clinical trial
Source: PLoS Med. 2026 Jul 10;23(7):e1005154. doi: 10.1371/journal.pmed.1005154 (PMC13354008; doi:10.1371/journal.pmed.1005154)
Supplement: Attachment — Submitted filename: All responses_02.04.2026.docx [file pmed.1005154.s010.docx]

|  | **Editor** | |
| --- | --- | --- |
| 1. | * Please confirm that your title complies with to PLOS Medicine's style. Your title must be nondeclarative and not a question. It should begin with main concept if possible. "Effect of" should be used only if causality can be inferred, i.e., for an RCT. Please place the study design ("A randomized controlled trial," "A retrospective study," "A modelling study," etc.) in the subtitle (ie, after a colon).  Editorial suggestion: Compression therapies for venous leg ulcers: The VENous Ulcer Study (VenUS 6), an open, multicentre, randomised clinical trial | We thank the editor for flagging this request. We have reviewed and accept the suggested amendment to the title with the addition of the VenUS study number: Compression therapies for venous leg ulcers: The VENous Ulcer Study 6 (VenUS 6), an open, multicentre, randomised clinical trial |
| 2. | * Statistical reporting: Please revise throughout the manuscript, including tables and figures. - Please report statistical information as follows to improve clarity for the reader ""22% (95% CI [13,28]; p</=)"". - Please separate upper and lower bounds with commas instead of hyphens as the latter can be confused with reporting of negative values. - Please repeat statistical definitions (HR, CI etc.) for each set of parentheses. | The manuscript has been reviewed and amended as requested where necessary.  We have reviewed and amended the formatting here as requested. |
| 3. | * Please ensure that all abbreviations are defined at first use throughout the text (including statistical abbreviations). | We have reviewed and ensured all abbreviations are defined at first use. |
| 4. | * Please ensure that tables and figures, including those in supplementary files, are appropriately referenced in the main text. | We have reviewed and ensured all tables and figures in both the main report and supplementary files are referenced in the text. |
| 5. | * Please review your text for claims of novelty or primacy (e.g. 'for the first time' or ‘novel’) and remove this language. | We have reviewed the manuscript and amended where required. |
| 6. | * Please confirm that any use of statistical terms (such as trend or significant) are supported by the data, and if not please remove them. The term trend should be used only when the test for trend has been conducted. | We have reviewed the manuscript and amended where required. |
| 7. | * Please provide titles and legends for all figures and tables (including those in Supporting Information files). | The manuscript and Supplementary material have been reviewed titles and legends added for all figures and tables if not already provided. |
| 8. | * Please define all acronyms used in each figure or table in its corresponding legend. | The manuscript and Supplementary material have been reviewed and acronyms defined in the legend as required. |
| 9. | * Please revise for use of patient-centered language. Please note that patient-centered language is constructed with the use of post-modified nouns (e.g. 'patients with psoriasis’ (or similar) instead of ‘psoriasis patients’) putting the person first in the sentence structure. | We have reviewed to ensure the manuscript is compliant with this request |
| 10. | * Causal language - In trials, there is usually a distinction in the language in terms of causal vs associational for primary and secondary trial outcomes. It would be beneficial to use associational language in the discussion and other sections for secondary outcomes. | Thank you for this interesting comment. about causation vs association for primary and then secondary outcomes.  We agree with the reviewer that even in an RCT, causal inferences rest on the appropriateness of the scientific and analytic assumptions that underpin them. In response we have checked our wording to ensure that we are careful about the attribution of causation.  However, we don’t see the same distinction as the reviewer regarding the (in)appropriateness of causal conclusions with respect to the primary and secondary outcomes. Despite potential differences in importance, statistical power/precision and risks of bias between the results for primary and secondary outcomes, the appropriateness of any causal conclusions rests on the validity of the assumptions and analytical methods underpinning these results, rather than whether or not they are considered to be primary or secondary. Again, we have checked the appropriateness of the language used to present findings throughout.  Following some of the edits to address other reviewer comments, we believe that our interpretation of results is appropriate. In particular, for analyses where causal conclusions would clearly rest on strong or implausible assumptions (e.g. the analyses of ulcer recurrence), we avoid causal language entirely. For example, when describing the results of the analyses for the recurrence outcome (which necessarily was restricted to the subset of participants that had healing reported), we frame the results in terms of association (E.g. *Allocation to EBC was associated with…*). |
| 11. | * Financial Disclosure (l.628ff): Please update the metadata in the online submission form with the details provided here. | We have removed this from the manuscript and included in the online submission form. |
| 12. | * Thank you for agreeing to make your data available. At this time, please provide the link to the data repository and accession numbers required for access. | We have added details of the repository and access details as requested. |
| 13. | ABSTRACT * Please confirm that your abstract complies with our requirements, including providing all the information relevant to this study type <https://journals.plos.org/plosmedicine/s/submission-guidelines#loc-abstract> | We have reviewed the journal guidance to ensure the abstract corresponds. |
| 14. | * Per CONSORT, please note that only the primary outcome of the trial should be reported in your Abstract. Secondary outcomes should only be included in the Abstract if all secondary outcomes are fully reported. For trials that have many secondary outcomes, the Abstract should be limited to reporting the primary outcome. You may mention the secondary outcomes in the Methods section, but do not report the results. | We have revised the abstract to remove reference to our secondary outcome results. |
| 15. | * Please confirm that all numbers presented in the abstract are present and identical to numbers presented in the main manuscript text.  * In the abstract, please include the important dependent variables that are adjusted for in the analyses.  * In the last sentence of the Abstract Methods and Findings section, please describe the main limitation(s) of the study's methodology.  * Please state that analysis was modified intention to treat.  * Please indicate if the trial met the pre-specified endpoint(s).  * Please provide the number of participants lost to follow up in each group. | We have reviewed the abstract and confirm numbers correspond to those in the main body of the manuscript.  We have added brief details of the explanatory variables that were included in the primary analysis to the Methods and Findings section of the abstract. This material also clarifies that participants were included in the analyses as part of the groups to which they were randomly allocated (in accordance with intention to treat principles).  The key limitation has been added to the abstract as requested.  We have amended the abstract to reflect a modified ITT analysis was used.  The inclusion of the limitation in the abstract provides relevant information with regards completion or not of prespecified end points and attrition in relation to the primary outcome. |
| 16. | * Please briefly explain the superiority and non-inferiority comparisons, or at least state that the main outcome is based on superiority comparisons. | We have added a sentence to the abstract that states that the comparison of two-layer bandage with evidence-based compression adopted a non-inferiority perspective, and comparisons of these treatment strategies with compression wraps adopted a superiority test perspective. |
| 17. | * “Time to healing was slower in the compression wrap group compared with both evidence-based compression (hazard ratio (HR): 0·78; 95% CI 0·61 to 1·00; p = 0·05) and two-layer bandage groups (HR: 0·79; 95% CI 0·61 to 1·01; p = 0·06) respectively. – Based on the numerical results, we find that the statement is not sufficiently accurate. The time to healing appears to be comparable, with numerical findings crossing 1 and p-values not showing statistical significance. Would these numbers have clinical significance? Please revise and report that the superiority of the compression wrap was not established. | Thank you for flagging this important point. The HR point estimate here is an important difference being a 22 or 21% relative reduction in the hazard of healing. Whilst the point estimate for time to healing was slower in the compression wrap group, we have made clearer the uncertainty around the estimate here. |
| 18. | AUTHOR SUMMARY * Please remove the bullet point from the questions.  * In the final bullet point of ‘What Do These Findings Mean?’, please clearly state the main limitation(s) of the study in non-technical language.  * Under “What Did the Researchers Do and Find?”, please describe the study methods in lay language in one or two bullet points.  * “In this randomized clinical trial of 637 80 UK participants with venous leg ulcers..” – please revise according to our above comment for accuracy. | We have removed the bullet points as requested  We have added the main limitation in lay language as requested.  We have added a lay summary of study results and methods and have actioned this clarification as requested |
| 19. | METHODS AND RESULTS * Secondary Outcomes: Please transform the bullet points into full sentences.  * Please remove the paragraph on role of the funding source. This information should only be included in the metadata in the online submission form. | We have removed the bullet points from the secondary outcomes as requested.  The funding statement has been removed as requested. |
| 20. | *Figure 2: The file is of low quality, and the numbers and text are small and nearly unreadable. The figure included in the main text appears to be higher quality, but the numbers and text are still small. Please revise. | We have sought to improve the readability of the figures as attached separately with this resubmission. |
| 21. | * “Rates of healing were seemingly slower in the CW group compared with both the EBC group (HR = 0·78 95% CI 0·61 to 1·00, p = 0·046) and the 2LB group (HR = 0·79 95% CI 0·61 to 1·01, p = 0·056) (Table 2, eFigure 3,eTable 4), although hypotheses positing essentially no difference between CW and these other treatments are also not conclusively refuted by the observed data. – Please note that, compared to the abstract, the reporting here appears to be more transparent. However, we recommend revising the statement "although hypotheses positing essentially no difference between CW and these other treatments are also not conclusively refuted by the observed data" to use clearer and more concise language (test of superiority). * l.428, “although the data are also compatible with hypotheses positing…” – please see our comment above. Please revise throughout. | We have revised in line with the abstract to present an interpretation of the 95% confidence intervals to reflect uncertainty around the trial’s point estimates of effect.  We have amended instances of ‘hypotheses positing’ to ‘there being’ throughout. |
| 22. | * “However, some estimates are uncertain, with 95% confidence intervals including potentially important differences.” – please revise for clarity. | As requested we have reviewed and amended this sentence for additional clarity |
| 23. | * “One event was deemed possibly related as cause of death was unknown at the time of study closure (2LB=1).” – can you disclose which one? | Unfortunately, there is very limited information for this death occurring in a patient allocated to be offered 2-layer bandaging. The patient was deceased out of their local area and the GP was unable to provide a cause of death. We have clarified as far as possible in the text. |
| 24. | * Please confirm that you provided the unadjusted comparisons as well as the adjusted comparisons in all relevant Tables.  * Please confirm that you specified the variables controlled for in all relevant Tables. | Thank you for this question. We can confirm that for the healing time outcomes, ulcer recurrence outcome, and participant reported VEINES questionnaire outcomes, we have reported the results from analyses that condition on the baseline covariates specified in our SAP, including the variables used for stratification of the randomisation (as is recommended in e.g. Kahan, B.C. and Morris, T.P. (2012), Improper analysis of trials randomised using stratified blocks or minimisation. Statist. Med., 31: 328-340. <https://doi.org/10.1002/sim.4431>).  Given that the randomisation was stratified by two key prognostic variables (ulcer area and duration), and following the approach taken in the analysis of many RCTs (and recommended in much of the statistical/methodological literature, for example Kahan, B.C., Jairath, V., Doré, C.J. et al. The risks and rewards of covariate adjustment in randomized trials: an assessment of 12 outcomes from 8 studies. Trials 15, 139 (2014). https://doi.org/10.1186/1745-6215-15-139), we would prefer to avoid adding results from analyses without covariate adjustment to the manuscript. The inclusion of these additional results would substantially complicate and lengthen the reporting, whilst providing little to no additional value to the reader. We suggest this is why the approach is not often seen RCT reporting in major journals. Indeed, submission guidelines for this journal do not stipulate routine presentation of both adjusted and unadjusted estimates, and this approach to reporting has not been taken in recent articles disseminating RCT results published in this journal (e.g. Kawamoto A, Hashida M, Ishida K, Furihata K, Ota A, et al. (2025) Immunogenicity and safety of DS-5670d, an omicron XBB.1.5-targeting COVID-19 mRNA vaccine: A phase 3, randomized, active-controlled study. PLOS Medicine 22(10): e1004499. https://doi.org/10.1371/journal.pmed.1004499). We also note that this approach would represent a departure from our SAP (which did not specify the reporting of unadjusted estimates for most of our outcomes) |
| 25. | * In this test of superiority it is not possible to determine that the intervention and control are equivalent, just that the intervention is not superior to the control. Please confirm that you reported the findings accordingly. | We agree that failure to reject the null hypothesis in a test of superiority should not be taken to imply/prove equivalence and have attempted to reflect this throughout our discussion of the results (e.g. by explicitly acknowledging the remaining uncertainty regarding the comparative effectiveness of CW compared with EBC, rather than simply stating that the null was rejected at the 5% level).  We have however added a caveat to the Conclusions section to explicitly state that uncertainty remains regarding the inferiority of two-layer bandaging compared with the evidence-based compression treatment strategy |
| 26. | * Please ensure that where relevant figures include 95% CIs. | We have reviewed the manuscript and 95% confidence intervals are provided for all between-group comparisons. |
| 27. | DISCUSSION * Please remove the 'conclusions' subheading from the discussion. Please also remove any other subheadings from the discussion. | The discussion section has been reviewed and all subheadings removed. |
| 28. | * “The level of removal and application is one mechanism that may explain the findings observed.” – Although we understand that this topic will be discussed in a separate publication, could you please explain it a bit more here? | We have added additional information to support this statement. |
| 29. | * RE the reference in l.483, “(submitted for publication)”, papers cannot be listed as such until they have been accepted for publication or are publicly available on a preprint archive. The information may be cited in the text as a personal communication with the author if the author provides written permission to be named. Alternatively please provide a different appropriate reference. | The publication is currently under review with another journal and has not been uploaded to a pre-print server. We are therefore unable to provide a reference currently and have revised the text to comply with journal requirements. |
| 30. | * In the paragraph titled "Strengths and Limitations," you seem to focus more on the limitations than the strengths of the trial. We think the strengths could be highlighted more. | We have reviewed the manuscript and added a paragraph to focus on the strengths of this trial. |
| 31. | * We also feel that there is little discussion of the existing evidence. Please keep this in mind when revising the manuscript. | We have reviewed and reflected on the links to existing evidence in the discussion section as suggested. |
| 32. | * You stated in the discussion that due to the COVID-19 pandemic the rate of receipt observed in this study is likely to have been significantly reduced because of restrictions and NHS pressures. Please re-consider completing the CONSERVE-CONSORT checklist and providing it in your Supporting Information. While no direct modification to the trial protocol were made, it appears that the trial was affected by extenuating circumstances, When completing the checklist, please use section and paragraph numbers, rather than page numbers. | A conserve checklist has been completed as requested, although as noted no direct modifications were made to the trial. |

|  | **Academic Editorial** | |
| --- | --- | --- |
| 1. | This is a nice study and the manuscript looks reasonable. There appear to be a couple of issues principally related to interpretation of results and presentation of conclusions. Currently this reads as a head to head trial of different compression methods, and that's not quite the case. | We thank the academic editor for their positive comments and helpful reflection. |
| 2. | As reviewer #2 pointed out, EBC was a treatment strategy rather than a single treatment. In the EBC arm, the choice was between 4LB and 2LH, based on clinical judgment and patient preference. Additionally, these compression treatments could be switched. Therefore, the estimates of effect are against a treatment strategy, not against specific types of compression. It would be better if this were clearer in the title, abstract, and conclusion. Additionally, I suggest moving the initial split of compression types within EBC and the extent of switching that occurred to the main paper. | We have clarified in the study design and participants section that EBC is technically a treatment strategy.  We have also, as requested, added the plot showing treatment receipt over time to the main body of the manuscript as Figure 2. |
| 3. | There seems to be inconsistency regarding the recommendation for two-layer bandaging. In the results, the non-inferiority of the two-layer bandage (2LB) was not established. However, the conclusion states that the results support a four-layer bandage, a two-layer hosiery, or a two-layer bandage as first-line options. | We have added a caveat to the Conclusions section to explicitly state that uncertainty remains regarding the inferiority of two-layer bandaging compared with the evidence-based compression treatment strategy |
| 4. | The results for CW vs. EBC and CW vs. 2LB are also borderline. CW did not shorten healing time, and healing may actually be slower. However, the abstract and conclusion are much more definitive. | We have amended the abstract to make this clearer. |
| 5. | Finally, the introductory paragraph on the aetiology of venous ulcers is confusing and not entirely accurate. Venous ulcers are not caused by arterial insufficiency, neuropathy or diabetes, except in cases of mixed aetiology. They are caused by chronic venous disease/insufficiency. The next paragraph states that compression therapy aims to reduce venous pressure. This should read 'venous hypertension'. | We apologise that this paragraph remains confusing. We have amended this further |
| 6. | **Reviewer 1** | |
| 7. | 1) The original request to note the underlying reason behind venous leg ulcers' origin hasn't been addressed. We are now aware that microcirculation plays an important role (see Tew GA et al., Eur J Appl Physiol. 2018 Feb;118(2):321-329) and this isn't noted in the references cited. A relevant reference should be added. | We apologise that this was not adequately addressed in the first revision. We have amended this further and also cited accordingly. |
| 8. | 2) Citation 38 should be removed. The fact that you are undertaking supporting work is encouraging, but as this is something under-writing it should be quoted as (unpublished data). | The publication is currently under review with another journal. We are therefore unable to provide a reference currently and have revised the text to comply with journal requirements. |
