# Supplementary material for: Compression therapies for venous leg ulcers: The VENous Ulcer Study 6 (VenUS 6), an open, multicentre, randomised clinical trial
Source: PLoS Med. 2026 Jul 10;23(7):e1005154. doi: 10.1371/journal.pmed.1005154 (PMC13354008; doi:10.1371/journal.pmed.1005154)
Supplement: Attachment — Submitted filename: Response to reviewers.docx [file pmed.1005154.s011.docx]

Response to reviewers

* *You meant to use ITT for analysis of the non-inferiority comparison, but you. don't include that result in the Abstract. You need to do so, specifying that it's an ITT. Separately you use a mITT for the superiority analyses, and state that in the Abstract, which is fine, but you still need to modify the following language.*
We have revised the text in the abstract to provide the estimates for both of the treatment effect estimands targeted as part of the non-inferiority comparisons, ensuring the description of these matches the language used earlier on the 'Methods and Findings' subsection, and have provided brief interpretation of these estimates with respect to the pre-specified non-inferiority margin.

* *You say: " Time to healing was slower in the compression wrap group compared with the both evidence-based compression (hazard ratio (HR): 0·78; (95% CI [0·61 to, 1·00]; p = 0·05) and two-layer bandage groups (HR: 0·79; (95% CI [0·61, to 1·01]; p = 0·06) respectively, with the 95% CI intervals ranging from a 39% reduction in time to healing to there being no difference between groups." You need to change to "Time to healing was slower in the compression wrap group compared with the evidence-based compression group....and showed no significant difference in duration compared with the two-layer bandage group..."*

We have revised discussion of the results of the superiority comparisons in the abstract to state explicitly that the hypothesis test undertaken for the comparison of CW and 2LB was not statistically significant at the two-sided 5% level. We have not followed the suggested language verbatim on the basis that it may erroneously suggest to a reader with limited training in statistics that the results for CW vs EBC and CW vs 2LB comparisons should be interpreted entirely differently on the basis of a p-value of 0.046 for the former and 0.056 for the latter, when in fact these p-values indicate remarkably similar information against the respective null hypotheses (approximately 4.4 bits for the CW vs EBC comparison, compared with 4.2 bits for the CW vs 2LB comparison). This choice reflects the advice provided in much recent statistical literature concerning the interpretation of frequentist hypothesis tests (for example *Rafi, Z., & Greenland, S. (2020). Semantic and cognitive tools to aid statistical science: replace confidence and significance by compatibility and surprise. BMC medical research methodology, 20(1), 244.* [*https://doi.org/10.1186/s12874-020-01105-9*](https://doi.org/10.1186/s12874-020-01105-9))

* *Please provide the transparency throughout the manuscript how many people switched treatment regimens (that point needs to be very transparent)*

We have added text to the results section explicitly stating the number and proportion of randomised participants that had any time not receiving their assigned compression treatment between randomisation and ulcer healing (or trial exit due to withdrawal, death or administrative censoring). We have also added material to both the Abstract and Discussion sections explicitly stating that the results provide information concerning the comparative effectiveness of allocation to the various treatments studied, and may provide limited information concerning the effectiveness of the treatments if used under 'ideal' settings with perfect adherence.
