# Supplementary material for: Compression therapies for venous leg ulcers: The VENous Ulcer Study 6 (VenUS 6), an open, multicentre, randomised clinical trial
Source: PLoS Med. 2026 Jul 10;23(7):e1005154. doi: 10.1371/journal.pmed.1005154 (PMC13354008; doi:10.1371/journal.pmed.1005154)
Supplement: Peer review history

# Editor report: Decision Letter 0

**DOI:** 10.1371/journal.pmed.1005154.r001

**Authors:** Richard Turner

23 Oct 2025

Dear Dr Arundel,

Thank you for submitting your manuscript entitled "VENous Ulcer Study (VenUS 6): An open, multi-centre, randomised clinical trial of compression therapies for the treatment of venous leg ulcers" for consideration by PLOS Medicine.

Your manuscript has now been evaluated by the PLOS Medicine editorial staff, and I am writing to let you know that we would like to send your submission out for external assessment.

However, we first need you to complete your submission by providing the metadata that are required for full assessment. To this end, please login to Editorial Manager where you will find the paper in the 'Submissions Needing Revisions' folder on your homepage. Please click 'Revise Submission' from the Action Links and complete all additional questions in the submission questionnaire.

For clinical studies, please upload a copy of your trial study protocol as a supporting information file. The study protocol should be the version submitted for approval to the institutional review board or ethics committee, should include any amendments to the study protocol, as well as the date of their approval by the institutional review or ethics committee. Please also detail any deviations from the study protocol in the Methods section of your manuscript. The editors will consider the protocol and study conduct prior to a final decision for external review.

Please re-submit your manuscript within two working days, i.e. by Oct 27 2025 11:59PM.

Login to Editorial Manager here: https://www.editorialmanager.com/pmedicine

Once your full submission is complete, your paper will undergo a series of checks in preparation for external assessment.

Feel free to email us at plosmedicine@plos.org if you have any queries relating to your submission.

Kind regards,

Richard Turner, PhD

Consulting Editor, PLOS Medicine

plosmedicine@plos.org

# Peer review: Decision Letter 1

**DOI:** 10.1371/journal.pmed.1005154.r002

**Authors:** Richard Turner, Alexandra Tosun

18 Dec 2025

Dear Dr Arundel,

Many thanks for submitting your manuscript "VENous Ulcer Study (VenUS 6): An open, multi-centre, randomised clinical trial of compression therapies for the treatment of venous leg ulcers" (PMEDICINE-D-25-03650R1) to PLOS Medicine. The paper has been reviewed by subject experts and a statistician; their comments are included below and can also be accessed here: [LINK]

As you will see, the reviewers found the trial interesting and important. However, they ask for clarification on the study design and raise concerns about treatment switching and the study being underpowered. After discussing the paper with the editorial team, I'm pleased to invite you to revise the paper in response to the reviewers' comments. We plan to send the revised paper to some or all of the original reviewers, and we cannot provide any guarantees at this stage regarding publication.

In addition to these revisions, you may need to complete some formatting changes, which you will receive in a follow up email. A member of our team will be in touch with a set of requests shortly. If you do not receive a separate email within a few days, please assume that checks have been completed, and no additional changes are required.

When you upload your revision, please include a point-by-point response that addresses all of the reviewer and editorial points, indicating the changes made in the manuscript and either an excerpt of the revised text or the location (eg: page and line number) where each change can be found. Please also be sure to check the general editorial comments at the end of this letter and include these in your point-by-point response. When you resubmit your paper, please include a clean version of the paper as the main article file and a version with changes tracked as a marked-up manuscript. It may also be helpful to check the guidelines for revised papers at http://journals.plos.org/plosmedicine/s/revising-your-manuscript for any that apply to your paper.

We ask that you submit your revision by Jan 29. However, if this deadline is not feasible, please contact me by email, and we can discuss a suitable alternative.

Due to the upcoming holiday season, the journal will operate at reduced capacity from December 22 to January 2. This will cause delays in the manuscript process. We appreciate your understanding.

Don't hesitate to contact me directly with any questions (atosun@plos.org).

Best regards,

Alexandra

Alexandra Tosun, PhD

Senior Editor

PLOS Medicine

atosun@plos.org

-----------------------------------------------------------

Comments from the reviewers:

Reviewer #1: Thank you for sharing the findings of this interesting study.

I only have some minor comments to make:

a) Introduction (Line 82): It would be probably worth to note the underlying reason behind venous leg ulcers. Noting only that they are primarily caused by "damaged or diseased" veins is not sufficient and as we now have a better idea as of how VLUs originate (see Tew GA et al., Eur J Appl Physiol. 2018 Feb;118(2):321-329), it would be good to present it considering that treatment effectiveness is related to how they appear in the first place.

b) Introduction (Lines 105-106): Please give brief examples of the type of evidence that is missing to reach a conclusion on which type of compression is more clinically effective.

c) Methods (Lines 153-154): Please explain in brief the reasoning behind the choice of your stratification criteria.

d) Methods (Line 165): Is there a citation for what is standard UK routine practice?

e) Methods (Lines 188-193): Considering that data collection was uneven, it would probably be important to have a table presenting how many patients completed each data collection stage. Figure 1 does seem incomplete without it. It is also positive that you do refer to this under trial limitations, but without presenting clearly the full numbers in the main text, the effect that the missing data has on the study conclusions is inconclusive.

It would also be important to add under "study limitations" also the fact that follow-ups were carried out by postal questionnaires and telephone follow-ups.

f) Methods (Lines 232-234): Why did you opt for 80% power (with a 10% loss of follow-up), considering that having a complete dataset would probably be not achievable?

g) Results: This section is quite well presented. I would recommend that you reduce the word count (as most of the text is covered by the tables) and that you add a "." at the end of Line 400.

h) Discussion: I would suggest that the authors are a bit more careful with the use of the English language (i.e.,"likely" and "likelihood" are used together in lines 414-415).

The authors spent also two paragraphs (Lines 412-427) repeating statements that already exist under results and although I agree that the key messages can be repeated, this doesn't need to be so extensive.

Moreover what I don't see in the discussion is the authors' explanation and translation of their results. For example, I don't see the authors theorising as of why there is no real difference between these two compression methods. There is an "Implication to Practice" paragraph at the end, but this probably needs to be before "Strengths and Implications", as well as it needs to go a bit more in depth, than simply re-stating what is covered elsewhere, extensively.

Finally, mentioning endovenous ablation (Lines 435-438) at the end of the Discussion does seem a bit odd, particularly as there wasn't any previous discussion on alternative treatment options (surgical or non-surgical): this does seem incomplete, despite the statement in Line 438 being correct.

Reviewer #2: "VENous Ulcer Study (VenUS 6): An open, multi-centre, randomised clinical trial of compression therapies for the treatment of venous leg ulcers" reports a randomized controlled trial (RCT) on the clinical effectiveness of compression wraps and two-layer compression bandages, against evidence-based compression. The three-arm trial was conducted from 2021 to 2024 in 33 UK sites, on 637 participants that were assigned approximately equally to each of the trial arms. It was found that time to healing was slower in the compression wrap group compared against evidence-based compression and two-layer bandages, although there were no statistically significant differences for other clinically-relevant outcomes.

While the findings are relevant to the practical treatment of venous leg ulcers, the existence (and possibly prevalence) of treatment switching is a concern that warrants further assessment. Other issues might also be considered:

1. In the Study design subsection, it is stated that EBC involves either 4LB or 2LH, with the choice between the two later clarified to be based on clinical judgment and/or patient preference. It might be considered to state the actual split between the two choices (if consistently applied throughout).

2. For the trial profile flowchart in Figure 1, the interaction between (full) withdrawal from clinical follow-up and the availability of clinical follow-up data, might be clarified. For example, for the EBC arm, there were 19 withdrawals, but all n=213 participants were then stated to have clinical follow-up data available.

3. In the Participants subsection, it is stated that the largest eligible ulcer at baseline was deemed the reference ulcer, and the leg on which it was located as the reference leg. It might be clarified as to whether a patient might require compression treatment on both legs, and if so, whether there was any limitation on the treatment received on the non-reference leg. If prevalent, the proportion of patients that required compression on both legs might be stated.

4. Further, it might be clarified if patients might have received any other therapy or treatment, that could have affected any of the reported clinical outcomes.

5. In the Primary Outcome subsection, the definition of the gaiter area and the measurement procedure for ulcers (e.g. ruler, tracing, planimetry etc.) might be briefly described. If possible, reference image(s) might be included, possibly as supplementary material.

6. In Line 216, "well know" might be "well known".

7. The Secondary outcomes subsection might be reorganized due to sentence fragments such as "Treatment of incompetent veins... death" in Line 220. In particular, it might be considered to explicitly label individual secondary outcomes, possibly as a numerical list.

8. Clinical definitions as applied for the various secondary outcomes (e.g. ulcer/skin deterioration) might be included if relevant, possibly in supplementary material.

9. In the Sample Size subsection, the assumption of an exponential distribution (e.g. prior work, empirical fitting on historical evidence) might be briefly justified, and the parameter of the exponential distribution stated.

10. In the Results section, it is stated that many participants switched compression treatments from the one allocated at some point between randomisation and healing. While admittedly possibly common in clinical practice, it might be clarified as to whether this switching was addressed in the analysis of the results. If possible, subsidiary analyses involving only patients who had maintained the same (assigned) treatment throughout, might be presented.

Reviewer #3: Firstly I would like to congratulate the team on bringing such a difficult and complex trial through to completion.

This is a good and vital study.

I have some comments, which hopefully will improve the manuscript.

Participants and endovenous ablation - patients were eligible if they had treatment scheduled after 4 weeks of baseline visit? And of those with leg ulcers only 29 patients had any form of treatment during follow-up? Was this not due to COVID more than any other factor? This needs to discussed a bit further as I would be concerned about participating centres not treating sufficiently (foam sclerotherapy counts as endovenous ablation) if there had not been a pandemic.

No duplex data was obtained to suggest spread of venous insufficiency in the groups, is that correct?

The null hypothesis was that CW and EBC showed the same healing rate.

A description of estimands and some of the statistics would be beneficial for generalisability. Perhaps as a supplement.

How was compliance with CW instructions measured - time in place and pressure applied?

The study under-recruited, and median times to healing were longer than expected. Are you satisfied that the study achieved the appropriate power?

What was the concordance of the blinded assessment of healing?

To the non-statistician the primary outcome paragraph does not make sense. For the EBC vs 2LB, both the non-inferiority estimates cross 1.

This unless explained better suggests to the reader that both situations (policy and hypothetical) are the same.

In addition the phrase "rates of healing were seemingly slower..." but a HR at 1 and a p value of 0.05 suggest no statical difference.

One would happily read this as no evidence of superiority between CW and 2LB or EBC, and no evidence of inferiority between 2LB and EBC. This then suggests essentially equivalence throughout. Most people will not read the supplement so whilst this appears clearer in eTable4 I think some adjustment is needed.

This would fit far more with the lack of difference seen in all the other outcomes described.

IF this is not the case then I suggest careful re-writing.

Conclusion:

If the likelihood of healing confidence with CW compared to EBC is -39% up to 0%, then this suggests an underpowered study. Clearly CW does not appear superior however, which is important. The incidence of infection was not statistically different, and therefore one should not state it is higher.

Clearly this is a completex study, which has provided evidence about compression strategies. However, the manuscript I believe needs some work to make it more useful to the reader and Clearer.

Any attachments provided with reviews can be seen via the following link: [LINK]

--------------------------------------------------------- ---

General editorial requests:

(Note: not all will apply to your paper, but please check each item carefully)

*We ask every co-author listed on the manuscript to fill in a contributing author statement, making sure to declare all competing interests. If any of the co-authors have not filled in the statement, we will remind them to do so when the paper is revised. If all statements are not completed in a timely fashion this could hold up the re-review process. If new competing interests are declared later in the revision process, this may also hold up the submission. Should there be a problem getting one of your co-authors to fill in a statement we will be in contact. Please do not add or remove authors without first discussing this with the handling editor. You can see our competing interests policy here: http://journals.plos.org/plosmedicine/s/competing-interests.

*Please upload any figures associated with your paper as individual TIF or EPS files with 300dpi resolution at resubmission; please read our figure guidelines for more information on our requirements: http://journals.plos.org/plosmedicine/s/figures. While revising your submission, we strongly recommend that you use PLOS's NAAS tool (https://ngplosjournals.pagemajik.ai/artanalysis) to test your figure files. NAAS can convert your figure files to the TIFF file type and meet basic requirements (such as print size, resolution), or provide you with a report on issues that do not meet our requirements and that NAAS cannot fix.

After uploading your figures to PLOS's NAAS tool - https://ngplosjournals.pagemajik.ai/artanalysis, NAAS will process the files provided and display the results in the "Uploaded Files" section of the page as the processing is complete.

If the uploaded figures meet our requirements (or NAAS is able to fix the files to meet our requirements), the figure will be marked as "fixed" above. If NAAS is unable to fix the files, a red "failed" label will appear above.

When NAAS has confirmed that the figure files meet our requirements, please download the file via the download option, and include these NAAS processed figure files when submitting your revised manuscript.

*Please ensure that the paper adheres to the PLOS Data Availability Policy (see http://journals.plos.org/plosmedicine/s/data-availability), which requires that all data underlying the study's findings be provided in a repository or as Supporting Information. For data residing with a third party, authors are required to provide instructions with contact information (web or email address) for obtaining the data. Please note that a study author cannot be the contact person for the data. PLOS journals do not allow statements supported by "data not shown" or "unpublished results." For such statements, authors must provide supporting data or cite public sources that include it.

*We expect all researchers with submissions to PLOS in which author-generated code underpins the findings in the manuscript to make all author-generated code available without restrictions upon publication of the work. In cases where code is central to the manuscript, we may require the code to be made available as a condition of publication. Authors are responsible for ensuring that the code is reusable and well documented. Please make any custom code available, either as part of your data deposition or as a supplementary file. Please add a sentence to your data availability statement regarding any code used in the study, e.g. "The code used in the analysis is available from Github [URL] and archived in Zenodo [DOI link]" Please review our guidelines at https://journals.plos.org/plosmedicine/s/materials-software-and-code-sharing and ensure that your code is shared in a way that follows best practice and facilitates reproducibility and reuse. Because Github depositions can be readily changed or deleted, we encourage you to make a permanent DOI'd copy (e.g. in Zenodo) and provide the URL.

*In the Ethics statement in the Methods section, please clearly state whether informed consent was written or oral.

FORMATTING - GENERAL

*Abstract: Please structure your abstract using the PLOS Medicine headings (Background, Methods and Findings, Conclusions). Please combine the Methods and Findings sections into one section.

*At this stage, we ask that you include a short, non-technical Author Summary of your research to make findings accessible to a wide audience that includes both scientists and non-scientists. The Author Summary should immediately follow the Abstract in your revised manuscript. This text is subject to editorial change and should be distinct from the scientific abstract. Ideally each sub-heading should contain 2-3 single sentence, concise bullet points containing the most salient points from your study. In the final bullet point of 'What Do These Findings Mean?', please include the main limitations of the study in non-technical language. Please see our author guidelines for more information: https://journals.plos.org/plosmedicine/s/revising-your-manuscript#loc-author-summary.

*Please express the main results with 95% CIs as well as p values. When reporting p values please report as p<0.001 and where higher as the exact p value p=0.002, for example. Throughout, suggest reporting statistical information as follows to improve clarity for the reader "22% (95% CI [13%,28%]; p</=)". Please be sure to define all numerical values at first use.

*Please include page numbers and line numbers in the manuscript file. Use continuous line numbers (do not restart the numbering on each page).

*Please cite the reference numbers in square brackets. Citations should precede punctuation.

FIGURES AND TABLES

*Please provide titles and legends for all figures and tables (including those in Supporting Information files).

*Please define all abbreviations used in each figure/table (including those in Supporting Information files).

*Please consider avoiding the use of red and green in order to make your figure more accessible to those with color blindness.

SUPPLEMENTARY MATERIAL

*Please note that supplementary material will be posted as supplied by the authors. Therefore, please amend it according to the relevant comments outlined here.

*Please cite your Supporting Information as outlined here: https://journals.plos.org/plosmedicine/s/supporting-information

REFERENCES

*PLOS uses the numbered citation (citation-sequence) method and first six authors, et al.

*Please ensure that journal name abbreviations match those found in the National Center for Biotechnology Information (NCBI) databases (http://www.ncbi.nlm.nih.gov/nlmcatalog/journals), and are appropriately formatted and capitalised.

*Where website addresses are cited, please include the complete URL and specify the date of access (e.g. [accessed: 12/06/2023]).

*Please also see https://journals.plos.org/plosmedicine/s/submission-guidelines#loc-references for further details on reference formatting.

STUDY TYPE-SPECIFIC REQUESTS

*PLOS Medicine requires that all trials be prospectively registered in one of registries recognized by WHO. Please ensure that study registration details are included in the Methods section.

*Please structure the Methods section using the following sub-headings: Study design and participants, Randomization and masking, Procedures, Outcomes, Statistical analysis.

*Please outline any outcome measures that differ between the submitted manuscript and the protocol [and/or trial registry]. Please clarify and explain all discrepancies between the paper and protocol. If the outcomes were not prespecified in the protocol, please define them in the Methods (Outcomes section) as post hoc and explain why they were added. Post-hoc comparisons should be presented as hypothesis generating rather than conclusive.

* Is the outcome on 'resource use' included in the paper?

* Will the semi-structured interviews exploring the views and experiences of participants and nurses on the different compression therapies be reported in a separate paper? If so, please mention this in the Methods section.

*Please ensure that all prespecified outcomes (primary, secondary, and exploratory) are listed in the Methods/Outcomes section and indicate whether there are outcomes that are not presented in the current report.

*Please specify the dates (Month Day, Year) during which study enrollment and follow up occurred.

*Please include absolute numbers wherever you report percentages; eg, n/N (%)

*Please present the safety data for the study including numbers of specific events and whether or not adverse events are thought to be related to treatment. AEs should be reported in the abstract, per CONSORT and CONSORT-Harms.

*Please complete the CONSORT checklist (https://www.equator-network.org/reporting-guidelines/consort/) and ensure that all components of CONSORT are present in the manuscript, including how randomization was performed, allocation concealment, blinding of intervention, definition of lost to follow-up, power statement. When completing the checklist, please use section and paragraph numbers, rather than page numbers.

* We also encourage authors to complete the SPIRIT 2025 checklist, which highlights the relevant information in the study protocol for readers.

*Please report your abstract according to CONSORT for abstracts, following the PLOS Medicine abstract structure (Background, Methods and Findings, Conclusions) https://www.equator-network.org/reporting-guidelines/consort-abstracts/

*If your trial had to undergo important modifications in response to extenuating circumstances, please complete the CONSERVE-CONSORT checklist and provide in your Supporting Information; (https://www.equator-network.org/reporting-guidelines/guidelines-for-reporting-trial-protocols-and-completed-trials-modified-due-to-the-covid-19-pandemic-and-other-extenuating-circumstances-the-conserve-2021-statement/). When completing the checklist, please use section and paragraph numbers, rather than page numbers.

*In keeping with our commitment to Open Science, please include the study protocol document and analysis plan (including any amendments) as Supporting Information to be published with the manuscript if accepted.

*Please note that PLOS Medicine requires prospective, public registration of a data sharing plan (as part of mandatory clinical trials registration) for all clinical trials that began enrollment on or after January 1, 2019, in accordance with ICMJE requirements.

# Author comment: Author response to Decision Letter 2

**DOI:** 10.1371/journal.pmed.1005154.r003

17 Feb 2026

Attachment Submitted filename: Response to Editorial requests 02.02.26.docx

# Peer review: Decision Letter 2

**DOI:** 10.1371/journal.pmed.1005154.r004

**Authors:** Richard Turner, Alexandra Tosun, Alexandra Tosun

27 Mar 2026

Dear Dr. Arundel,

Thank you very much for re-submitting your manuscript "VENous Ulcer Study (VenUS 6): An open, multi-centre, randomised clinical trial of compression therapies for the treatment of venous leg ulcers" (PMEDICINE-D-25-03650R2) for review by PLOS Medicine.

Thank you for your detailed response to the reviewers' and editors’ comments. I have discussed the paper with my colleagues, and it has also been seen again by the original reviewers. The changes made to the paper were mostly satisfactory to the reviewers. Please note that the Academic Editor has provided a set of comments which the editorial team strongly agrees with. Please carefully address these issues in a further revision, taking into account the comments of the reviewers and editors below. When submitting your revised paper, please once again include a detailed point-by-point response to the editorial comments. The remaining issues that need to be addressed are listed at the end of this email.

In revising the manuscript for further consideration here, please ensure you address the specific points made by each reviewer and the editors. In your rebuttal letter you should indicate your response to the reviewers' and editors' comments and the changes you have made in the manuscript. Please submit a clean version of the paper as the main article file. A version with changes marked must also be uploaded as a marked up manuscript file. Please also check the guidelines for revised papers at http://journals.plos.org/plosmedicine/s/revising-your-manuscript for any that apply to your paper.

In addition to these revisions, you may need to complete some formatting changes, which you will receive in a follow up email. A member of our team will be in touch with a set of requests shortly. If you do not receive a separate email within a few days, please assume that checks have been completed, and no additional changes are required.

Please note, when your manuscript is accepted, an uncorrected proof of your manuscript will be published online ahead of the final version, unless you've already opted out via the online submission form. If, for any reason, you do not want an earlier version of your manuscript published online or are unsure if you have already indicated as such, please let the journal staff know immediately at plosmedicine@plos.org.

We ask that you submit your revision by Apr 03 2026. However, if this deadline is not feasible, please contact the journal staff by email, and we can discuss a suitable alternative.

We look forward to receiving the revised manuscript.

Sincerely,

Alexandra Tosun, PhD

Senior Editor

PLOS Medicine

plosmedicine.org

***Please note while forming your response, if your article is accepted, you may have the opportunity to make the peer review history publicly available. The record will include editor decision letters (with reviews) and your responses to reviewer comments. If eligible, we will contact you to opt in or out.***

------------------------------------------------------------

Comments from the Academic Editor:

This is a nice study and the manuscript looks reasonable. There appear to be a couple of issues principally related to interpretation of results and presentation of conclusions. Currently this reads as a head to head trial of different compression methods, and that's not quite the case.

As reviewer #2 pointed out, EBC was a treatment strategy rather than a single treatment. In the EBC arm, the choice was between 4LB and 2LH, based on clinical judgment and patient preference. Additionally, these compression treatments could be switched. Therefore, the estimates of effect are against a treatment strategy, not against specific types of compression. It would be better if this were clearer in the title, abstract, and conclusion. Additionally, I suggest moving the initial split of compression types within EBC and the extent of switching that occurred to the main paper.

There seems to be inconsistency regarding the recommendation for two-layer bandaging. In the results, the non-inferiority of the two-layer bandage (2LB) was not established. However, the conclusion states that the results support a four-layer bandage, a two-layer hosiery, or a two-layer bandage as first-line options.

The results for CW vs. EBC and CW vs. 2LB are also borderline. CW did not shorten healing time, and healing may actually be slower. However, the abstract and conclusion are much more definitive.

Finally, the introductory paragraph on the aetiology of venous ulcers is confusing and not entirely accurate. Venous ulcers are not caused by arterial insufficiency, neuropathy or diabetes, except in cases of mixed aetiology. They are caused by chronic venous disease/insufficiency. The next paragraph states that compression therapy aims to reduce venous pressure. This should read 'venous hypertension'.

------------------------------------------------------------

Comments from Reviewers:

Reviewer #1: Thank you for responding to the original comments.

I have two minor suggestions:

1) The original request to note the underlying reason behind venous leg ulcers' origin hasn't been addressed. We are now aware that microcirculation plays an important role (see Tew GA et al., Eur J Appl Physiol. 2018 Feb;118(2):321-329) and this isn't noted in the references cited. A relevant reference should be added.

2) Citation 38 should be removed. The fact that you are undertaking supporting work is encouraging, but as this is something under-writing it should be quoted as (unpublished data).

Reviewer #2: We thank the authors for addressing our previous comments, and the additional post-hoc analyses for patients who maintained their allocated treatment for most of the study.

Reviewer #3: Thank you for your adjustments. The manuscript is much clearer and concise on the statements and outcomes.

Well done.

------------------------------------------------------------

Requests from Editors:

GENERAL

* Please confirm that your title complies with to PLOS Medicine's style. Your title must be nondeclarative and not a question. It should begin with main concept if possible. "Effect of" should be used only if causality can be inferred, i.e., for an RCT. Please place the study design ("A randomized controlled trial," "A retrospective study," "A modelling study," etc.) in the subtitle (ie, after a colon).

Editorial suggestion: Compression therapies for venous leg ulcers: The VENous Ulcer Study (VenUS 6), an open, multicentre, randomised clinical trial

* Statistical reporting: Please revise throughout the manuscript, including tables and figures.

- Please report statistical information as follows to improve clarity for the reader ""22% (95% CI [13,28]; p</=)"".

- Please separate upper and lower bounds with commas instead of hyphens as the latter can be confused with reporting of negative values.

- Please repeat statistical definitions (HR, CI etc.) for each set of parentheses.

* Please ensure that all abbreviations are defined at first use throughout the text (including statistical abbreviations).

* Please ensure that tables and figures, including those in supplementary files, are appropriately referenced in the main text.

* Please review your text for claims of novelty or primacy (e.g. 'for the first time' or ‘novel’) and remove this language.

* Please confirm that any use of statistical terms (such as trend or significant) are supported by the data, and if not please remove them. The term trend should be used only when the test for trend has been conducted.

* Please provide titles and legends for all figures and tables (including those in Supporting Information files).

* Please define all acronyms used in each figure or table in its corresponding legend.

* Please revise for use of patient-centered language. Please note that patient-centered language is constructed with the use of post-modified nouns (e.g. 'patients with psoriasis’ (or similar) instead of ‘psoriasis patients’) putting the person first in the sentence structure.

* Causal language - In trials, there is usually a distinction in the language in terms of causal vs associational for primary and secondary trial outcomes. It would be beneficial to use associational language in the discussion and other sections for secondary outcomes.

* Financial Disclosure (l.628ff): Please update the metadata in the online submission form with the details provided here.

* Thank you for agreeing to make your data available. At this time, please provide the link to the data repository and accession numbers required for access.

ABSTRACT

* Please confirm that your abstract complies with our requirements, including providing all the information relevant to this study type https://journals.plos.org/plosmedicine/s/submission-guidelines#loc-abstract

* Per CONSORT, please note that only the primary outcome of the trial should be reported in your Abstract. Secondary outcomes should only be included in the Abstract if all secondary outcomes are fully reported. For trials that have many secondary outcomes, the Abstract should be limited to reporting the primary outcome. You may mention the secondary outcomes in the Methods section, but do not report the results.

* Please confirm that all numbers presented in the abstract are present and identical to numbers presented in the main manuscript text.

* In the abstract, please include the important dependent variables that are adjusted for in the analyses.

* In the last sentence of the Abstract Methods and Findings section, please describe the main limitation(s) of the study's methodology.

* Please state that analysis was modified intention to treat.

* Please indicate if the trial met the pre-specified endpoint(s).

* Please provide the number of participants lost to follow up in each group.

* Please briefly explain the superiority and non-inferiority comparisons, or at least state that the main outcome is based on superiority comparisons.

* “Time to healing was slower in the compression wrap group compared with both evidence-based compression (hazard ratio (HR): 0·78; 95% CI 0·61 to 1·00; p = 0·05) and two-layer bandage groups (HR: 0·79; 95% CI 0·61 to 1·01; p = 0·06) respectively. – Based on the numerical results, we find that the statement is not sufficiently accurate. The time to healing appears to be comparable, with numerical findings crossing 1 and p-values not showing statistical significance. Would these numbers have clinical significance? Please revise and report that the superiority of the compression wrap was not established.

AUTHOR SUMMARY

* Please remove the bullet point from the questions.

* In the final bullet point of ‘What Do These Findings Mean?’, please clearly state the main limitation(s) of the study in non-technical language.

* Under “What Did the Researchers Do and Find?”, please describe the study methods in lay language in one or two bullet points.

* “In this randomized clinical trial of 637 80 UK participants with venous leg ulcers..” – please revise according to our above comment for accuracy.

METHODS AND RESULTS

* Secondary Outcomes: Please transform the bullet points into full sentences.

* Please remove the paragraph on role of the funding source. This information should only be included in the metadata in the online submission form.

*Figure 2: The file is of low quality, and the numbers and text are small and nearly unreadable. The figure included in the main text appears to be higher quality, but the numbers and text are still small. Please revise.

* “Rates of healing were seemingly slower in the CW group compared with both the EBC group (HR = 0·78 95% CI 0·61 to 1·00, p = 0·046) and the 2LB group (HR = 0·79 95% CI 0·61 to 1·01, p = 0·056) (Table 2, eFigure 3,eTable 4), although hypotheses positing essentially no difference between CW and these other treatments are also not conclusively refuted by the observed data. – Please note that, compared to the abstract, the reporting here appears to be more transparent. However, we recommend revising the statement "although hypotheses positing essentially no difference between CW and these other treatments are also not conclusively refuted by the observed data" to use clearer and more concise language (test of superiority).

* l.428, “although the data are also compatible with hypotheses positing…” – please see our comment above. Please revise throughout.

* “However, some estimates are uncertain, with 95% confidence intervals including potentially important differences.” – please revise for clarity.

* “One event was deemed possibly related as cause of death was unknown at the time of study closure (2LB=1).” – can you disclose which one?

* Please confirm that you provided the unadjusted comparisons as well as the adjusted comparisons in all relevant Tables.

* Please confirm that you specified the variables controlled for in all relevant Tables.

* In this test of superiority it is not possible to determine that the intervention and control are equivalent, just that the intervention is not superior to the control. Please confirm that you reported the findings accordingly.

* Please ensure that where relevant figures include 95% CIs.

DISCUSSION

* Please remove the 'conclusions' subheading from the discussion. Please also remove any other subheadings from the discussion.

* “The level of removal and application is one mechanism that may explain the findings observed.” – Although we understand that this topic will be discussed in a separate publication, could you please explain it a bit more here?

* RE the reference in l.483, “(submitted for publication)”, papers cannot be listed as such until they have been accepted for publication or are publicly available on a preprint archive. The information may be cited in the text as a personal communication with the author if the author provides written permission to be named. Alternatively please provide a different appropriate reference.

* In the paragraph titled "Strengths and Limitations," you seem to focus more on the limitations than the strengths of the trial. We think the strengths could be highlighted more.

* We also feel that there is little discussion of the existing evidence. Please keep this in mind when revising the manuscript.

* You stated in the discussion that due to the COVID-19 pandemic the rate of receipt observed in this study is likely to have been significantly reduced because of restrictions and NHS pressures. Please re-consider completing the CONSERVE-CONSORT checklist and providing it in your Supporting Information. While no direct modification to the trial protocol were made, it appears that the trial was affected by extenuating circumstances, When completing the checklist, please use section and paragraph numbers, rather than page numbers.

------------------------------------------------------------

General Journal Requests

1) We ask every co-author listed on the manuscript to fill in a contributing author statement. If any of the co-authors have not filled in the statement, we will remind them to do so when the paper is revised. If all statements are not completed in a timely fashion this could hold up the re-review process. Should there be a problem getting one of your co-authors to fill in a statement we will be in contact. YOU MUST NOT ADD OR REMOVE AUTHORS UNLESS YOU HAVE ALERTED THE EDITOR HANDLING THE MANUSCRIPT TO THE CHANGE AND THEY SPECIFICALLY HAVE AGREED TO IT.

2) Please ensure that the paper adheres to the PLOS Data Availability Policy (see http://journals.plos.org/plosmedicine/s/data-availability), which requires that all data underlying the study's findings be provided in a repository or as Supporting Information. For data residing with a third party, authors are required to provide instructions with contact information for obtaining the data. PLOS journals do not allow statements supported by "data not shown" or "unpublished results." For such statements, authors must provide supporting data or cite public sources that include it.

3) Please review your reference list to ensure that it is complete and correct. If you have cited papers that have been retracted, please include the rationale for doing so in the manuscript text, or remove these references and replace them with relevant current references. Any changes to the reference list should be mentioned in the rebuttal letter that accompanies your revised manuscript.

# Author comment: Author response to Decision Letter 3

**DOI:** 10.1371/journal.pmed.1005154.r005

16 Apr 2026

Attachment Submitted filename: All responses_02.04.2026.docx

# Editor report: Decision Letter 3

**DOI:** 10.1371/journal.pmed.1005154.r006

**Authors:** Richard Turner, Alexandra Tosun, Alexandra Tosun, Katarzyna Starowicz

26 May 2026

Dear Dr. Arundel,

Thank you very much for re-submitting your manuscript "Compression therapies for venous leg ulcers: The VENous Ulcer Study 6 (VenUS 6), an open, multicentre, randomised clinical trial " (PMEDICINE-D-25-03650R3) for review by PLOS Medicine.

I have discussed the paper with my colleagues and we would like you to address and provide some clarification on some remaining questions.

The remaining issues that need to be addressed are listed at the end of this email. Any accompanying reviewer attachments can be seen via the link below. Please take these into account before resubmitting your manuscript:

[LINK]

***Please note while forming your response, if your article is accepted, you may have the opportunity to make the peer review history publicly available. The record will include editor decision letters (with reviews) and your responses to reviewer comments. If eligible, we will contact you to opt in or out.***

In revising the manuscript for further consideration here, please ensure you address the specific points made by each reviewer and the editors. In your rebuttal letter you should indicate your response to the reviewers' and editors' comments and the changes you have made in the manuscript. Please submit a clean version of the paper as the main article file. A version with changes marked must also be uploaded as a marked up manuscript file.

Please also check the guidelines for revised papers at http://journals.plos.org/plosmedicine/s/revising-your-manuscript for any that apply to your paper. If you haven't already, we ask that you provide a short, non-technical Author Summary of your research to make findings accessible to a wide audience that includes both scientists and non-scientists. The Author Summary should immediately follow the Abstract in your revised manuscript. This text is subject to editorial change and should be distinct from the scientific abstract.

In addition to these revisions, you may need to complete some formatting changes, which you will receive in a follow up email. A member of our team will be in touch with a set of requests shortly. If you do not receive a separate email within a few days, please assume that checks have been completed, and no additional changes are required.

We expect to receive your revised manuscript within 1 week. Please email us (plosmedicine@plos.org) if you have any questions or concerns.

We ask every co-author listed on the manuscript to fill in a contributing author statement. If any of the co-authors have not filled in the statement, we will remind them to do so when the paper is revised. If all statements are not completed in a timely fashion this could hold up the re-review process. Should there be a problem getting one of your co-authors to fill in a statement we will be in contact. YOU MUST NOT ADD OR REMOVE AUTHORS UNLESS YOU HAVE ALERTED THE EDITOR HANDLING THE MANUSCRIPT TO THE CHANGE AND THEY SPECIFICALLY HAVE AGREED TO IT.

Please ensure that the paper adheres to the PLOS Data Availability Policy (see http://journals.plos.org/plosmedicine/s/data-availability), which requires that all data underlying the study's findings be provided in a repository or as Supporting Information. For data residing with a third party, authors are required to provide instructions with contact information for obtaining the data. PLOS journals do not allow statements supported by "data not shown" or "unpublished results." For such statements, authors must provide supporting data or cite public sources that include it.

To enhance the reproducibility of your results, we recommend that you deposit your laboratory protocols in protocols.io, where a protocol can be assigned its own identifier (DOI) such that it can be cited independently in the future. Additionally, PLOS ONE offers an option to publish peer-reviewed clinical study protocols. Read more information on sharing protocols at https://plos.org/protocols?utm_medium=editorial-email&utm_source=authorletters&utm_campaign=protocols

Please review your reference list to ensure that it is complete and correct. If you have cited papers that have been retracted, please include the rationale for doing so in the manuscript text, or remove these references and replace them with relevant current references. Any changes to the reference list should be mentioned in the rebuttal letter that accompanies your revised manuscript.

Please note, when your manuscript is accepted, an uncorrected proof of your manuscript will be published online ahead of the final version, unless you've already opted out via the online submission form. If, for any reason, you do not want an earlier version of your manuscript published online or are unsure if you have already indicated as such, please let the journal staff know immediately at plosmedicine@plos.org.

If you have any questions in the meantime, please contact me or the journal staff on plosmedicine@plos.org.

We look forward to receiving the revised manuscript by Jun 02 2026 11:59PM.

Sincerely,

Katarzyna Starowicz, PhD

Senior Editor

PLOS Medicine

plosmedicine.org

------------------------------------------------------------

Requests and comments from Editors:

* Please rephrase statements in both the Abstract and the Discussion so that conclusions reflect the data, and the data offer weak support as they are not in all instances statistically significant.

* You meant to use ITT for analysis of the non-inferiority comparison, but you. don't include that result in the Abstract. You need to do so, specifying that it's an ITT. Separately you use a mITT for the superiority analyses, and state that in the Abstract, which is fine, but you still need to modify the following language.

*You say: " Time to healing was slower in the compression wrap group compared 66

with the both evidence-based compression (hazard ratio (HR): 0·78; (95% CI [0·61 to, 67

1·00]; p = 0·05) and two-layer bandage groups (HR: 0·79; (95% CI [0·61, to 1·01]; p 68

= 0·06) respectively, with the 95% CI intervals ranging from a 39% reduction in time 69

to healing to there being no difference between groups." You need to change to "Time to healing was slower in the compression wrap group compared with the evidence-based compression group....and showed no significant difference in duration compared with the two-layer bandage group..."

* Please, edit the discussion to avoid strong claims and caveat their results to specify they cannot generalize. Adding a few more sentences pertaining to the statistical significance (or lack thereof) pertaining to the results would be helpful. You don't need a complete rewrite.

*Please provide the transparency throughout the manuscript how many people switched treatment regimens (that point needs to be very transparent)

Any attachments provided with reviews can be seen via the following link:

[LINK]

# Author comment: Author response to Decision Letter 4

**DOI:** 10.1371/journal.pmed.1005154.r007

1 Jun 2026

Attachment Submitted filename: Response to reviewers.docx

# Editor report: Decision Letter 4

**DOI:** 10.1371/journal.pmed.1005154.r008

**Authors:** Richard Turner, Alexandra Tosun, Alexandra Tosun, Katarzyna Starowicz, Katarzyna Starowicz

8 Jun 2026

Dear Dr Arundel,

On behalf of my colleagues and the Academic Editor, Karim Brohi, I am pleased to inform you that we have agreed to publish your manuscript "Compression therapies for venous leg ulcers: The VENous Ulcer Study 6 (VenUS 6), an open, multicentre, randomised clinical trial " (PMEDICINE-D-25-03650R4) in PLOS Medicine.

Before your manuscript can be formally accepted you will need to complete some formatting changes, which you will receive in a follow up email. Please be aware that it may take several days for you to receive this email; during this time no action is required by you. Once you have received these formatting requests, please note that your manuscript will not be scheduled for publication until you have made the required changes.

In the meantime, please log into Editorial Manager at http://www.editorialmanager.com/pmedicine/, click the "Update My Information" link at the top of the page, and update your user information to ensure an efficient production process.

PRESS

We frequently collaborate with press offices. If your institution or institutions have a press office, please notify them about your upcoming paper at this point, to enable them to help maximise its impact. If the press office is planning to promote your findings, we would be grateful if they could coordinate with medicinepress@plos.org. If you have not yet opted out of the early version process, we ask that you notify us immediately of any press plans so that we may do so on your behalf.

We also ask that you take this opportunity to read our Embargo Policy regarding the discussion, promotion and media coverage of work that is yet to be published by PLOS. As your manuscript is not yet published, it is bound by the conditions of our Embargo Policy. Please be aware that this policy is in place both to ensure that any press coverage of your article is fully substantiated and to provide a direct link between such coverage and the published work. For full details of our Embargo Policy, please visit http://www.plos.org/about/media-inquiries/embargo-policy/.

To enhance the reproducibility of your results, we recommend that you deposit your laboratory protocols in protocols.io, where a protocol can be assigned its own identifier (DOI) such that it can be cited independently in the future. Additionally, PLOS ONE offers an option to publish peer-reviewed clinical study protocols. Read more information on sharing protocols at https://plos.org/protocols?utm_medium=editorial-email&utm_source=authorletters&utm_campaign=protocols

Thank you again for submitting to PLOS Medicine. We look forward to publishing your paper.

Sincerely,

Katarzyna Starowicz, PhD

Associate Editor

PLOS Medicine
